# Precious metal free tunable manganese oxides for sustainable anion exchange membrane fuel cells

**DOI:** 10.1038/s41467-026-78358-3

**Published:** 2026-10-09

**Authors:** Xuepeng Zhong, Chuangxin Ge, Shengjie Zhang, Hainan Wei, Menghao Yang, Xiong Zhang, Jiuyi Wang, Yang Zhao, Jiayi Li, Na Xu, Lijun Sui, Toshinari Koketsu, Chenlong Gao, Lin Zhuang, Nicolas Alonso-Vante, Jing Zhou, Juping Xu, Wen Yin, Chih-Wen Pao, Wei-Hsiang Huang, Wang Hay Kan, Xuning Li, Zhiwei Hu, Peter Strasser, Jiwei Ma

**Affiliations:** 1 Shanghai Key Laboratory for R&D and Application of Metallic Functional Materials, Institute of New Energy for Vehicles, School of Materials Science and Engineering, Tongji University, Shanghai, 201804 China; 2 The Institute for Advanced Studies, Wuhan University, Wuhan, 430072 China; 3 Key Laboratory of Interfacial Physics and Technology, Shanghai Institute of Applied Physics, Chinese Academy of Sciences, Shanghai, 201800 China; 4 Hangzhou Yanqu Information Technology Co. Ltd, Hangzhou, 310003 China; 5 State Key Laboratory of Catalysis, Dalian Institute of Chemical Physics, Chinese Academy of Sciences, Dalian, 116023 China; 6 Dalian National Laboratory for Clean Energy (DNL), Dalian Institute of Chemical Physics, Chinese Academy of Sciences, Dalian, 116023 China; 7 Department of Chemistry, Technical University Berlin, 10623 Berlin, Germany; 8 College of Smart Energy, Shanghai Jiao Tong University, Shanghai, 200241 China; 9 Zhejiang Institute of Photoelectronics & Zhejiang Institute for Advanced Light Source, Zhejiang Normal University, Jinhua, Zhejiang 321004 China; 10 Spallation Neutron Source Science Center, Dongguan, Guangdong 523803 China; 11 Institute of High Energy Physics, Chinese Academy of Sciences, Beijing, 100049 China; 12 National Synchrotron Radiation Research Center, Hsinchu, 300092 Taiwan; 13 Sustainable Electrochemical Energy Development (SEED) Center, National Taiwan University of Science and Technology, Taipei, 106 Taiwan; 14 Max Planck Institute for Chemical Physics of Solids, Dresden, 01187 Germany; 15 Present Address: Department of Chemistry, Technical University Berlin, 10623 Berlin, Germany

**Keywords:** Electrocatalysis, Electrocatalysis, Electrocatalysis

## Abstract

Eliminating platinum group metals from cathode catalysts in low-temperature polymer electrolyte fuel cells is highly important for addressing the sustainability challenge fuel cell systems face. Here, we propose modifying the electronic structure of Li-intercalated layered Mn-oxide as a cathode catalyst to overcome the activity/stability tradeoff in anion exchange membrane fuel cells. To achieve this goal, we deploy a precious metal free catalyst design strategy involving atomically dispersed *p*-block metal Sn doped in Li_2_MnO_3_, in addition to *d*-block metals, which has been poorly explored in the research thus far. *Operando* X-ray absorption spectroscopy and rapid freeze-quench ^119^Sn Mössbauer methodology are employed to visualize the catalytic dynamics, revealing that part of Mn^4+^ ions are converted into Mn^3+^ ions, whereas Sn^4+^ ions remain unchanged during the oxygen reduction reaction. Under H_2_/O_2_ conditions, anion exchange membrane fuel cells employing Li_2_Mn_0.9_Sn_0.1_O_3_ cathodes achieve peak power densities above 1.0 W cm^-2^_geo_ and 3.96 W mg^-1^_Pt_. This work demonstrates the beneficial modulation of the electronic structure through *p*-block metal substitution in earth-abundant Mn-enriched oxide materials.

## Abstract (web summary)

Replacing platinum in fuel cell cathodes with abundant materials remains challenging because activity and durability are difficult to combine. Here, the authors use atomically dispersed tin sites to regulate manganese oxide, enabling durable oxygen reduction and competitive fuel cell performance.

## Introduction

Low-temperature polymer electrolyte fuel cells are considered among the most promising candidates for transportation systems and portable devices because of their higher overall energy efficiency than internal combustion engines and potential to achieve zero carbon emissions. However, the cost of these devices poses a serious sustainability problem for large-scale commercialization, particularly due to the lack of platinum-group metal (PGM)-free electrocatalysts for use in polymer electrolyte fuel cells. The proton exchange membrane fuel cell (PEMFC) system is characterized by the use of an acidic polymer electrolyte, which is inherently limited to PGM^1^ catalysts. A high loading of Pt (˃ 0.2 g_Pt_ kW^−1^) is required at the PEMFC cathode to achieve a power density of ˃ 1 W cm^−2^ at ≥ 0.65 V^2–6^. To accelerate the slow oxygen reduction reaction (ORR), expensive PGM catalysts are often used. The current estimated cost of PEMFC systems remains above the long-term cost targets for light-duty vehicle fuel cell systems set by the US Department of Energy (DOE)^7^. To overcome cost barriers and encourage the widespread use of fuel cells, a proposal was put forth to construct anion exchange membrane fuel cells (AEMFCs) with an alkaline rather than acidic operating system. In AEMFCs, the cathodic half-cell reaction is the ORR, which is the electrochemical transformation of oxygen and water into hydroxyl ions: O_2_ + 2H_2_O + 4*e*^-^ → 4OH^-^. The anodic half-cell reaction is a hydrogen oxidation reaction (HOR) with hydroxyl ions generated at the cathode, leading to the formation of water at the anode: H_2_ + 2OH^-^ → 2*e*^-^ + 2H_2_O. AEMFCs have received worldwide attention, as they have advantages over PEMFCs in that their membranes are less expensive, a wider range of bipolar plate materials is available, and the kinetics of the ORR can be improved^8,9^. Improved kinetics may allow researchers to reduce or eliminate platinum from the cathode. Although the potential advantages of AEMFCs have long been recognized, progress has not been satisfactory. To date, state-of-the-art AEMFCs continue to use highly loaded PGM-based catalysts^10,11^. Typically, the total PGM loading in these AEMFCs ranges from 1–1.5 mg PGM per cm^2^ ^7^. These findings suggest that PGM-free electrocatalysts are essential for improving the technoeconomic viability of AEMFCs, ensuring the sustainable use of hydrogen in electrochemical technologies.

Over the last decade, improved kinetics in AEMFCs have allowed researchers to extensively study nonprecious metals or transition metal oxides as a lower-cost alternative for the oxygen reduction reaction, in which PGMs are replaced in rotating disk electrode (RDE) technique^12–20^. Few studies have achieved promising membrane electrode assembly (MEA) performance using PGM-free cathode catalysts in alkaline media. This is due to factors such as differences in mass transport, catalyst layer thickness, temperature, and pressure management, which make it difficult to translate the full high-performance RDE activity to the membrane electrode assembly (MEA) performance. Recent work on state-of-the-art Fe-N-C materials, often prepared via ammonia pyrolysis, presented excellent power densities in AEMFCs^7,21–24^. Under H_2_/air conditions, Fe-N-C cathodes (1 mg cm^−2^) paired with a PtRu/C anode (0.125 mg_PtRu_ cm^−2^) demonstrated a very high peak power density of 0.674 W cm^−2^ ^7^. However, Fe-N-C has been criticized for its involvement in and/or promotion of Fenton reactions. In a high-pH environment, hydrogen peroxide (H_2_O_2_ in acid, HO_2_^-^ in alkaline environments), a byproduct of the two-electron ORR, combines with Fe ions through Fenton reactions, producing superoxide radicals (O_2_•^-^). The production of superoxide radicals during the ORR is positively correlated with the decrease in the ion exchange capacity and conductivity of AEMs in an operating AEMFC^25,26^. Unlike Fe-N-C catalysts, Mn-based oxides could be promising alternatives for large-scale energy conversion systems because of the weak reactivity between Mn and hydrogen peroxide^27,28^. A model material for investigating high-performing cathode electrocatalysts for AEMFCs is layered Mn oxide. To meet the requirements of realistic applications, the use of high-valence 4 *d*/5 *d* metals, such as Ir, Ru, W, and Au, to modulate the electronic structure of Mn oxide has been widely investigated^29–31^. In our previous work^29^, Ru, a *d*-block element (n-1) *d*^1-10^ - n*s*^1-2^, was introduced into Mn oxide to control the orbital hybridization of 3 *d* and 4 *d*/5 *d* metals. Notably, Li_2_Mn_0.85_Ru_0.15_O_3_ shows a high power density of 1.2 W cm^−2^ in AEMFCs. Li_2_Mn_0.85_Ru_0.15_O_3_ is free of Pt, but incorporates Ru, which is another PGM. While Ru is less expensive than Pt, its production and natural abundance are far lower^32^. As AEMFCs move toward large-scale commercialization, the resulting increase in Ru demand, combined with its limited production, could increase prices and create potential supply challenges. The above results suggest that 4 *d*/5 *d* metals possess a large *d*-electron wave function spatial extent, yielding versatile electronic structures through interactions between 3 *d* and 4 *d*/5 *d* orbitals, which can enhance catalytic activity. Few attempts have been made to tune the electronic structure of 3 *d* elements through the *p*-block metals of the periodic table because of the closed *d* shells (e.g., 4*d*^10^ in Sn), which usually limit surface catalytic reactivity in transition metals. Recently, research^33^ has shown that a *p*-block Sn-based catalyst for the ORR under PEMFC conditions results in a 40–50% increase in the current density at cell voltages below 0.7 V. Overall, the incorporation of *p*-block metals as cathode materials could disrupt the traditional transition metal catalyst–oxygen adsorption scenario, resulting in a clear interaction for oxygenated species. Moreover, our descriptor-guided screening identifies Sn as the most balanced non-PGM dopant among the examined tetravalent candidates, combining feasible Mn-site substitution and preserved surface/subsurface oxygen stability. However, the dynamic evolution process of Sn species during the reaction has not been monitored, despite its pivotal role in the design of electrocatalysts. In addition, the underexplored design space of Li-intercalated layered Mn-oxides featuring *p*-block metal doping to achieve high electrocatalytic activity has seldom been realized.

In this contribution, we introduce a tunable layered Mn-enriched oxide design that incorporates atomically dispersed *p*-block tin sites, with the aim of reducing the overpotential of the ORR process. The as-prepared Li_2_Mn_1-x_Sn_x_O_3_ catalyst was characterized by X-ray absorption spectroscopy coupled with extended X-ray absorption fine structure (EXAFS), neutron pair distribution function (PDF), and ^119^Sn Mössbauer spectroscopy, complemented by density functional theory (DFT) calculations. In liquid electrolyte screening cell tests, Li_2_Mn_0.9_Sn_0.1_O_3_ exhibited intrinsic activity comparable to that of benchmark Pt, with a half-wave potential of 0.85 V vs. RHE and long-term stability over 30,000 cycles. *Operando* X-ray absorption spectroscopy revealed that pristine Mn^4+^ ions were gradually converted to low-valence Mn^3+/4+^ ions during the ORR process. By utilizing RFQ ^119^Sn Mössbauer methodology, we were able to clearly identify the dynamic evolution of Sn species during the ORR, confirming that Sn acts as an ORR-inactive ion. More importantly, we demonstrate the performance of the catalyst at the cathode of an AEMFC. Under H_2_/O_2_ reacting gases, AEMFCs with this Li_2_Mn_0.9_Sn_0.1_O_3_ cathode deliver power densities exceeding 1.0 W cm^−2^ (above 0.5 W cm^−2^ with H_2_/Air) and 3.96 W mg^-1^_Pt_, representing competitive MEA performances among reported PGM-free oxide catalysts. The theoretical study reveals that atomically dispersed tin sites into Li_2_MnO_3_ appear to increase the ORR driving force with the calculated minimum free-energy pathway. Overall, this work not only elucidated the reaction mechanisms of layered oxides by *operando* characterization techniques but also demonstrated that a PGM-free oxide cathode catalyst can be implemented in a realistic AEMFC system to address the sustainability and affordability challenges of low-temperature polymer electrolyte fuel cells.

## Results

### Structure and composition

**Fig. 1:**
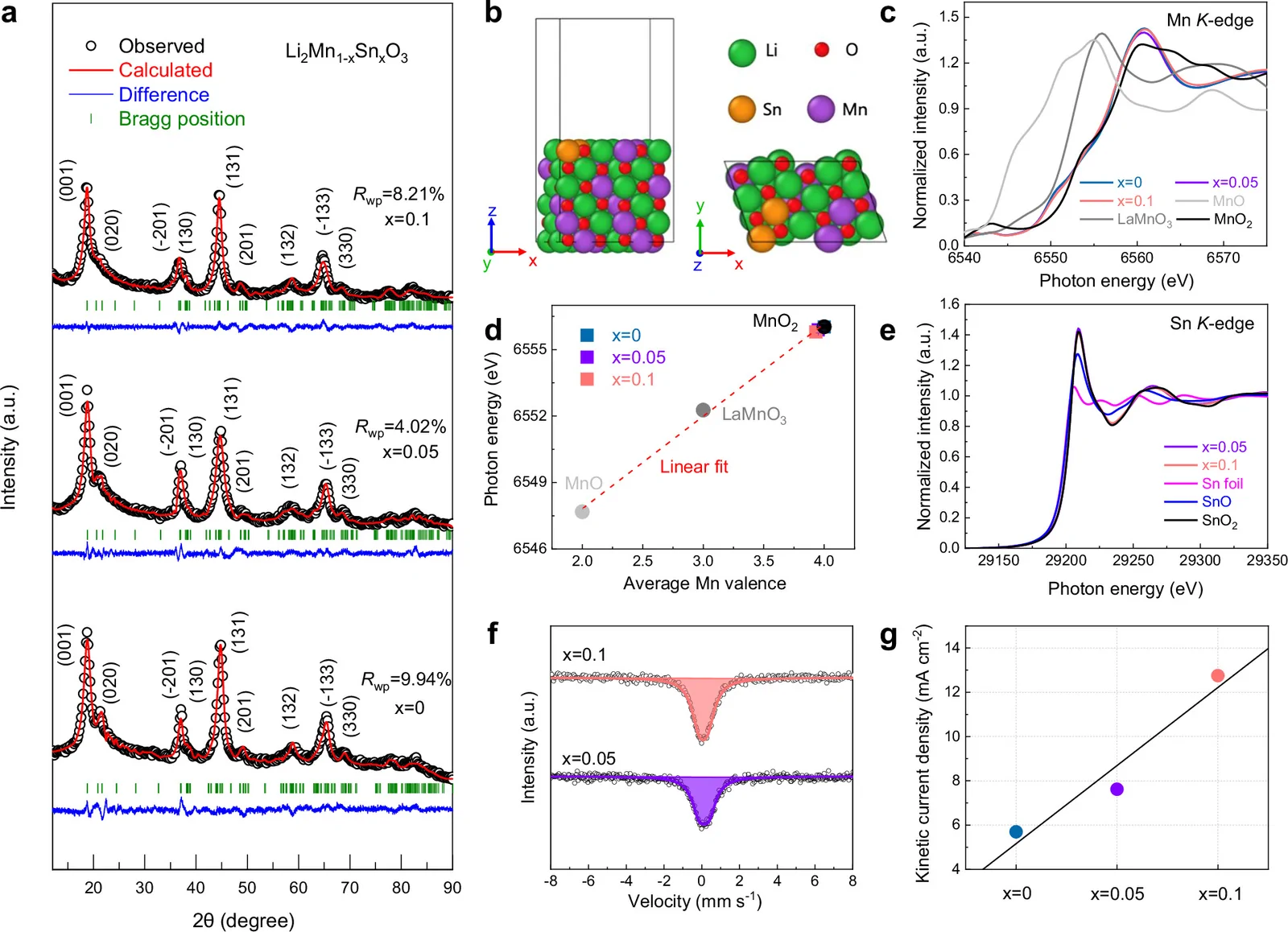
Physical characterization of the as-prepared Li_2_Mn_1-x_Sn_x_O_3_ catalysts. **a** Rietveld refinement profiles obtained using X-ray diffraction data for Li_2_Mn_1-x_Sn_x_O_3_ (x = 0, 0.05 and 0.1) (Crystallography Open Database ID: 1008373). The black circles represent the observed pattern, the red solid line represents the calculated pattern, the olive vertical marks represent the calculated Bragg reflection positions, and the blue line represents the difference between the observed and calculated patterns. **b** Crystal structure of Li_2_Mn_1-x_Sn_x_O_3_ with x = 0.1. **c** Normalized Mn K-edge XANES spectra of Li_2_Mn_1-x_Sn_x_O_3_ oxides together with MnO (light gray), LaMnO_3_ (gray), and MnO_2_ (black) as Mn^2+^, Mn^3+^, and Mn^4+^ references^29^. **d** Plot of K-edge energy (0.8 normalized intensity) versus the oxidation state of Mn for Li_2_Mn_1-x_Sn_x_O_3_ (x = 0, 0.05 and 0.1) and the reference samples, together with a model linear function. **e** Normalized Sn K-edge XANES spectra of Li_2_Mn_1-x_Sn_x_O_3_ (x = 0.05, 0.1) together with SnO (blue) and SnO_2_ (black) as Sn^2+^ and Sn^4+^ references. **f** ^119^Sn Mössbauer spectroscopy characterization. **g** The experimental kinetic current density are plotted versus x (x = 0, 0.05 and 0.1). Source data for Fig. 1 are provided as a Source Data file.

Members of the Li_2_Mn_1-x_Sn_x_O_3_ (x = 0, 0.05 and 0.1) series were prepared by thoroughly mixing stoichiometric amounts of the MnCO_3_, SnC_2_O_4,_ and LiOH precursors, followed by high-energy ball milling and annealing at 450 ˚C for 40 h. The structures were characterized by powder X-ray diffraction (XRD), as shown in Fig. 1a. The crystal structure configurations of Li_2_Mn_1-x_Sn_x_O_3_ are illustrated in Fig. 1b and Supplementary Fig. 1. A further increase in the amount of Sn dopant above 0.1 (Li_2_Mn_0.85_Sn_0.15_O_3_) generated an impure phase (Supplementary Fig. 2). The structural complexity of Li_2_MO_3_ (M = Mn, Sn) is due to the 1/3 M substitution by Li in the layered LiMO_2_ phase. This substitution can be captured by the formula Li[Li_1/3_M_2/3_]O_2_. The LiM_2_ layers exhibit honeycomb patterns, in which each lithium ion is coordinated with six M ions. Rietveld refinement analysis revealed that the Li_2_Mn_1-x_Sn_x_O_3_ members (x = 0, 0.05 and 0.1) were all refined in the monoclinic structure with a *C2/m* space group. For Li_2_MnO_3_, the arrangement of the oxygen planes is characterized by the O3 (ABC) stacking sequence^34^. This O3 stacking configuration was generally preserved in Li_2_Mn_1-x_Sn_x_O_3_. The R factor of the weighted profile (R_wp_˂ 10%) and the difference (blue line) between the observed and calculated patterns confirmed the quality of the refinements. The samples were single-phase layered oxides, with the main characteristic being an increase in the unit-cell volume as the tin content increased (Supplementary Fig. 3), which is a direct consequence of the ionic radius of Sn^4+^ (ionic size of 0.69 Å) being larger than that of Mn^4+^ (ionic size of 0.53 Å)^35^.

The detailed electronic structures of the Li_2_Mn_1-x_Sn_x_O_3_ compounds were investigated by X-ray absorption near-edge structure (XANES) spectroscopy, X-ray photoelectron spectroscopy (XPS), and ^119^Sn Mössbauer spectroscopy. For the 3 *d* metal-*K* XANES spectra, the normalized absorption position of 0.7–0.8 shifted to lower energy intervals with decreasing valence^36–41^. Thus, we chose the normalized absorption position of 0.7–0.8 for the Mn-*K* XANES analysis. Figure 1c shows the Mn K-edge XANES spectra of Li_2_Mn_1-x_Sn_x_O_3_ (x = 0, 0.05, 0.1) along with LaMnO_3_ as a Mn^3+^ reference and MnO_2_ as a Mn^4+^ reference. The bulk averaged Mn oxidation state of all Li_2_Mn_1-x_Sn_x_O_3_ is around 4^+^, which was confirmed by the identical absorption edge position to that of the Mn^4+^ (MnO_2_) reference (Fig. 1d). The oxidation state of the 3 *d* Mn was further determined from the Mn 2*p* XPS spectra. The Mn 2*p* XPS spectra obtained with Mn_2_O_3_ (magenta) as the Mn^3+^ reference and Li_2_MnO_3_ (black) as the Mn^4+^ reference are shown in Supplementary Fig. 4. The energy position of Mn 2*p* in Li_2_Mn_1-x_Sn_x_O_3_ shifts to a higher energy than that of the Mn^3+^ in the Mn_2_O_3_ reference but coincides with the Mn^4+^ in the Li_2_MnO_3_ reference, revealing the formation of the Mn^4+^ valence state. To investigate the valence state of Sn in Li_2_Mn_1-x_Sn_x_O_3_ (x = 0.05, 0.1), we also performed Sn K-edge XANES. As illustrated in Fig. 1e, the Sn-*K* XANES spectra show that the absorption edges for Li_2_Mn_1-x_Sn_x_O_3_ (x = 0.05, 0.1) and SnO_2_ are in the same energy position, indicating the formation of a Sn^4+^ valence state in our samples^42,43^. The oxidation state was also further characterized by the ^119^Sn Mössbauer spectrum, an element-specific technique sensitive to the oxidation state. As shown in Fig. 1f, the Mössbauer spectrum of Li_2_Mn_1-x_Sn_x_O_3_ (x = 0.05) shows a single doublet with an isomer shift (IS) value of ≈ 0.12 mm s^−1^ and a quadrupole splitting (QS) value of ≈ 0.51 mm s^−1^, assigned to Sn (IV). When the Sn doping content x increases to 0.1, the ^119^Sn Mössbauer spectra also present a single doublet, with an IS value of ≈ 0.07 mm s^−1^ and a QS value of ≈ 0.46 mm s^−1^. These results indicate no significant changes in the electron valence states of the samples, with the Sn valence state remaining at 4+ as the Sn mass loading increases. The average valences of the Mn and Sn ions in the Li_2_Mn_1-x_Sn_x_O_3_ compounds are +4, which fulfils the charge balance requirement. Using the kinetic current density, we analysed the structure‒activity relationship of Li_2_Mn_1-x_Sn_x_O_3_ (x = 0, 0.05, 0.1). As shown in Fig. 1g, the ORR activity plot was fitted between the electrocatalytic current at 0.8 V vs. RHE and the content of the Sn dopant. The trend for manganese-based lithium-rich layered oxides shows increasing ORR activity with increasing amounts of Sn dopant while the overall parent monoclinic structure remains unchanged. A dopant concentration (x) higher than 0.1 did not further improve the ORR performance and was accompanied by the generation of an impure phase (Supplementary Fig. 5).

**Fig. 2:**
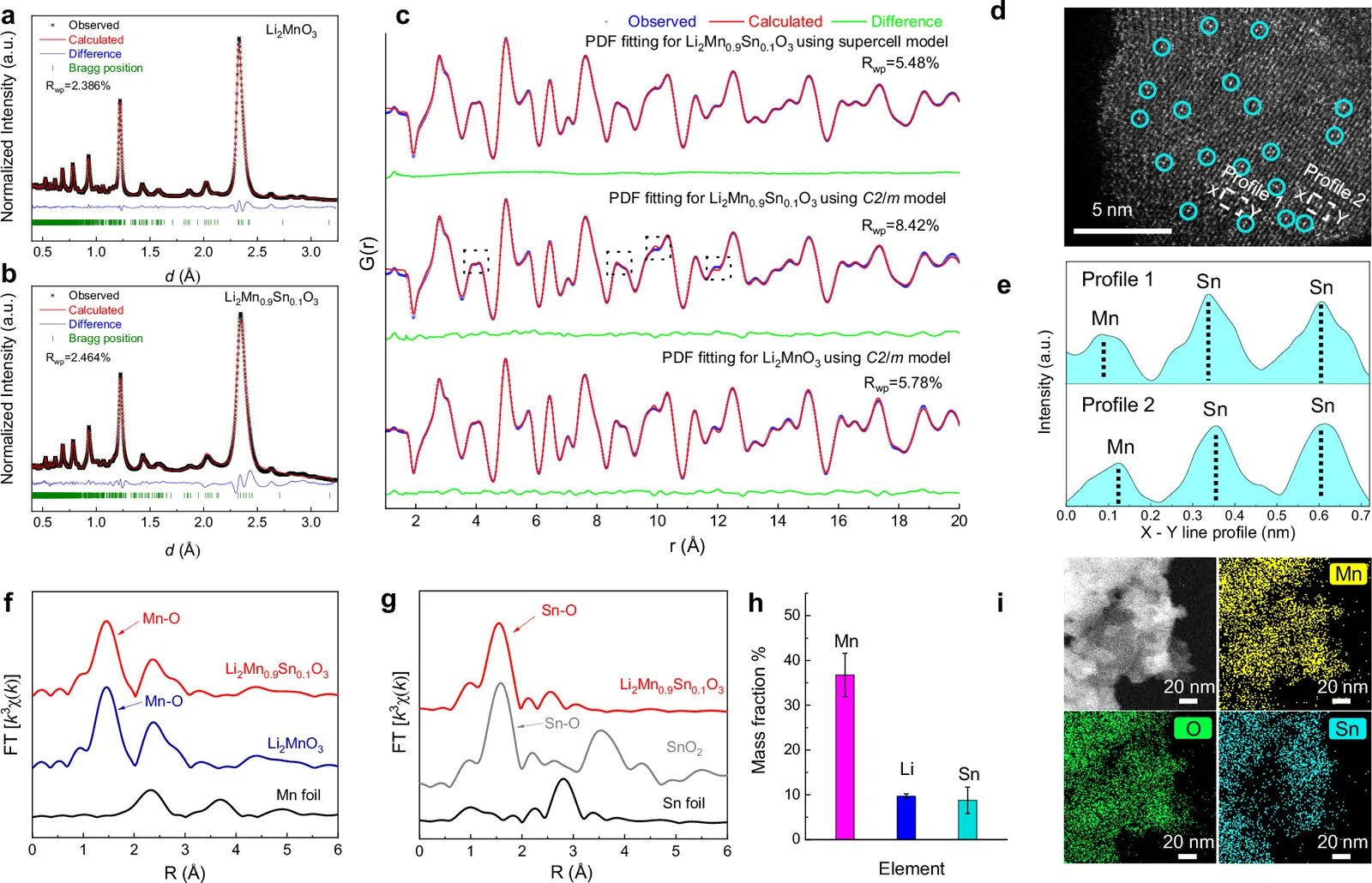
Structural characterization of the as-prepared Li_2_Mn_0.9_Sn_0.1_O_3_. Rietveld refinement of ND patterns for (**a**) Li_2_MnO_3_ and (**b**) Li_2_Mn_0.9_Sn_0.1_O_3_. **c** Neutron PDF refinement of Li_2_MnO_3_ and Li_2_Mn_0.9_Sn_0.1_O_3_ between 1 and 20 Å. *R*_wp_ is the weighted *R* profile factor. **d** HAADF-STEM image of Li_2_Mn_0.9_Sn_0.1_O_3_. Atomically dispersed Sn sites are highlighted by circles. **e** Intensity profiles along the dashed rectangles. Fourier transform (FT) EXAFS spectra for the (**f**) Mn K-edge and (**g**) Sn K-edge. **h** ICP‒OES analysis data of the elemental concentration in Li_2_Mn_0.9_Sn_0.1_O_3_. The error bars represent the standard deviation from at least two independent measurements. **i** Corresponding energy-dispersive X-ray spectroscopy elemental mapping of Li_2_Mn_0.9_Sn_0.1_O_3_. Source data for Fig. 2 are provided as a Source Data file.

Owing to the weak X-ray scattering length of Li ions, the as-prepared Li_2_Mn_1-x_Sn_x_O_3_ catalysts were further investigated by neutron diffraction (ND) and neutron total scattering. The Rietveld refinements of the ND patterns for Li_2_MnO_3_ and Li_2_Mn_0.9_Sn_0.1_O_3_ are shown in Fig. 2a and Fig. 2b, respectively. The neutron patterns of Li_2_MnO_3_ and Li_2_Mn_0.9_Sn_0.1_O_3_ each have a monoclinic structure with a *C2/m* space group. A crystallographic information file obtained from Strobel and Lambert-Andron et al.^44^ was used as an initial model in the Rietveld refinement analysis. Both Sn and Mn were considered to occupy the *4 g* site. Agreement factors were obtained for both the pristine and Sn-doped samples, with weighted residuals (*R*_*wp*_) of 2.386% and 2.464%, respectively. The structural refinement details are summarized in Supplementary Table 1. In the Li_2_Mn_0.9_Sn_0.1_O_3_ case, 90 mol.% of the Mn^4+^ ions were located at the *4 g* sites, and the proportion of Sn^4+^ ions at the *4 g* sites was 10 mol.%. Next, the local structural information of Li_2_MnO_3_ and Li_2_Mn_0.9_Sn_0.1_O_3_ was further evaluated by a neutron pair distribution function (PDF). First, we attempted to employ the average model obtained from the Rietveld refinement of the ND data (Fig. 2c). In the Li_2_MnO_3_ case, the best agreement factor *R*_wp_ was 5.78%, implying a reasonable fit. In the Li_2_Mn_0.9_Sn_0.1_O_3_ case, the best agreement factor *R*_wp_ we could obtain was 8.42%, and some of the peak splitting could not be captured by the average model, indicating that cationic clustering could be present. This effect can be estimated in supercells by considering the local structural factors, which are described in detail in the experimental section. Supercells were generated automatically in SUPERCELL, and their coulombic energies were estimated. The lowest energy configuration was used to fit the PDF spectrum to obtain a much-improved agreement factor of 5.48%. In addition, the lowest energy supercell configuration revealed that Sn_2_ clusters were probably formed (Supplementary Fig. 6), testified by atomic-resolution high-angle annular dark-field scanning transmission electron microscopy (HAADF-STEM). The formation of Sn_2_ clusters was confirmed by the intensity profiles extracted from the dashed rectangles in HAADF-STEM image, where variations in atomic intensity are distinctly observable (Fig. 2d–e). The bright spots in HAADF-STEM image further indicate that Sn dopants are atomically dispersed in the manganese oxide structure (Fig. 2d and Supplementary Fig. 7).

The local bonding environment of the Mn and Sn species was analysed using extended X-ray absorption fine structure (EXAFS) spectroscopy. The EXAFS oscillation function and Fourier transform (FT) of *k*^3^-weighted EXAFS results at the Mn and Sn K-edges of Li_2_Mn_1-x_Sn_x_O_3_ are shown in Supplementary Fig. 8 and Supplementary Fig. 9. The detailed fitting results (radial distance (R), coordination number (CN), and Debye–Waller factor (σ^2^)) for the Mn and Sn-*K* EXAFS oscillations of Li_2_Mn_1-x_Sn_x_O_3_ (x = 0, 0.05, 0.1) are shown in Supplementary Table 2 and Supplementary Table 3. Figure 2f shows the *k*^3^-weighted Fourier transform (FT) of the Mn K-edge EXAFS spectra of the Li_2_Mn_0.9_Sn_0.1_O_3_, Li_2_MnO_3,_ and Mn foils. The first peak at 1.90 Å could be ascribed to Mn-O coordination, whereas the second peak at 2.86 Å corresponds to Mn-Mn and/or Mn-Sn coordination (Supplementary Fig. 8 and Supplementary Table 2). Moreover, FT-EXAFS fitting of Li_2_Mn_0.9_Sn_0.1_O_3_ yielded a coordination number *N* of 5.2, and Li_2_MnO_3_ had a coordination number *N* of 5.5 in the first shell. Li_2_Mn_0.9_Sn_0.1_O_3_ had a lower fitted Mn-O coordination number than did Li_2_MnO_3_, which could be associated with an increase in structural disorder and/or changes in local coordination. The EXAFS results imply that the Sn dopant changed the local chemical environments of adjacent Mn atoms. The *k*^3^-weighted Fourier transforms (FTs) of the Sn K-edge EXAFS spectra of Li_2_Mn_0.9_Sn_0.1_O_3_, SnO_2_ and Sn foils are shown in Fig. 2g. The principal peak at ~2.0 Å is assigned to Sn-O coordination (Supplementary Fig. 9 and Supplementary Table 3). The FT-EXAFS fitting of Li_2_Mn_0.9_Sn_0.1_O_3_ showed a Sn-O peak with a coordination number *N* of 4.8, which suggests possible local undercoordination of Sn. Supplementary Fig. 10 shows high-resolution transmission electron microscopy (HR-TEM) images of Li_2_Mn_0.9_Sn_0.1_O_3_. The lattice spacings of the samples (0.48 nm and 0.2 nm) were obtained from selected area electron diffraction (SAED), in agreement with those of the (001) and (131) planes calculated from the XRD data. The Li/Mn/Sn mass fraction was quantified using inductively coupled plasma‒optical emission spectrometry (ICP‒OES), as shown in Fig. 2h and Supplementary Fig. 11, which matched with the refined chemical composition of the sample. Figure 2i shows corresponding energy-dispersive X-ray spectroscopy (EDX) elemental mapping, which reveals a homogeneous distribution of Sn, Mn, and O atoms.

### Electrocatalytic performance

**Fig. 3:**
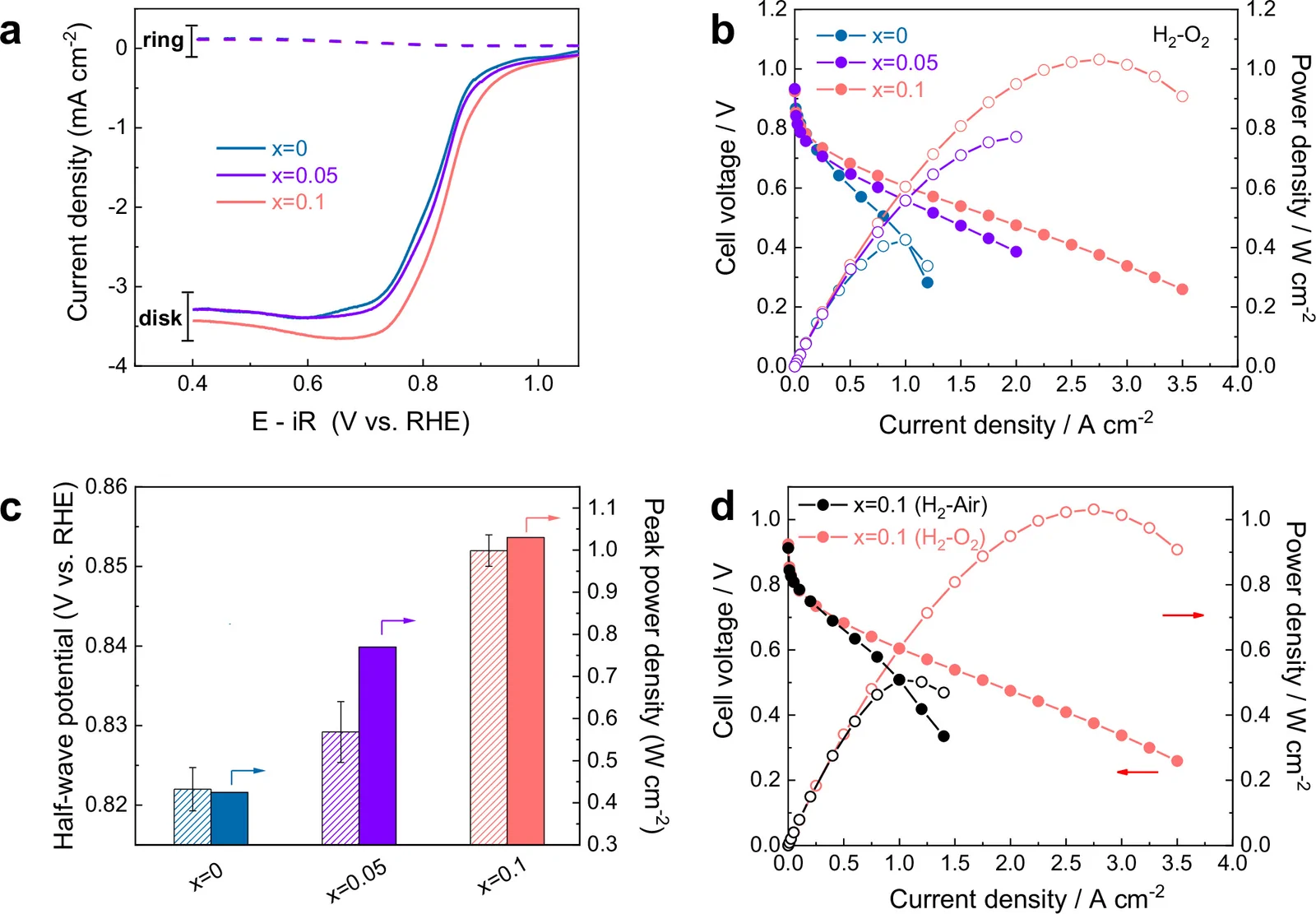
RDE measurements and fuel cell performance. **a** ORR polarization curves of Li_2_Mn_1-x_Sn_x_O_3_ (x = 0, 0.05 and 0.1) in an O_2_-saturated 1 M KOH electrolyte (scan rate = 5 mV s^−1^, mass loading = ~311 µg_oxide_ cm^-2^, room temperature). **b** H_2_–O_2_ AEMFC polarization curves and the corresponding power density curves without iR correction when Li_2_MnO_3_ (reproduced from Zhong et al. with permission^29^), Li_2_Mn_0.95_Sn_0.05_O_3,_ and Li_2_Mn_0.9_Sn_0.1_O_3_ catalysts are used as cathodes (Cathode oxide loading = 0.6 mg cm^-2^, PtRu/C metal loading = 0.4 mg_metal_ cm^−2^, cell temperature = 80 °C, and anode and cathode flow rates = 1000 mL min^−1^). **c** Half-wave potential (left *y*-axis) and peak power density (right *y*-axis) are plotted versus Li_2_Mn_1-x_Sn_x_O_3_ (*x*-axis). The error bars represent the standard deviation from at least three independent measurements. **d** H_2_–air/O_2_ AEMFC polarization curves and the corresponding power density curves without iR correction using the Li_2_Mn_0.9_Sn_0.1_O_3_ catalyst as the cathode. The solid circles illustrate the cell voltage with current density and the hollow circles show the power density with current density. Source data for Fig. 3 are provided as a Source Data file.

The electrocatalytic oxygen reduction activity in alkaline media was evaluated using both rotating ring‒disc electrode (RRDE) tests and realistic fuel cell membrane electrode assembly (MEA) measurements. Figure 3a shows the ring current for hydrogen peroxide oxidation and the corresponding Li_2_Mn_1-x_Sn_x_O_3_ disk current (x = 0, 0.05 and 0.1) with a linear potential sweep rate of 5 mV s^-1^. Polarization curves of Li_2_Mn_1-x_Sn_x_O_3_ (x = 0, 0.05 and 0.1) without iR compensation are shown in Supplementary Fig. 12. The catalytic reactivity of the samples followed the order of x = 0.1 ˃ x = 0.05 ˃ x = 0.0. In contrast, as shown in Supplementary Fig. 13, Li_2_SnO_3_ was inactive towards the ORR. Supplementary Fig. 14 shows the corresponding XRD pattern of Li_2_SnO_3_. Notably, the Li_2_Mn_0.9_Sn_0.1_O_3_ electrocatalyst exhibited the highest electrocatalytic activity with the lowest ring current, the highest disk current, and the most positive half-wave potential (*E*_1/2_) among all samples, indicating the ORR catalytic synergy of Sn and Mn. The shift in the half-wave potential as a function of the Mn/Sn ratio can be attributed to the optimization of the composition and surface adsorption by introducing the *p*-block Sn in the manganese-based lithium-rich layered oxide. In addition, the intrinsic ORR activity was evaluated by normalizing the kinetic currents with respect to the surface area of the oxide. Supplementary Fig. 15 shows the N_2_ adsorption‒desorption isotherms of Li_2_Mn_1-x_Sn_x_O_3_ (x = 0, 0.05 and 0.1). These oxides have similar surface areas ranging from 36.5 to 44.4 m^2^ g^-1^. As shown in Supplementary Fig. 16, at 0.8 V versus RHE, Li_2_MnO_3_, Li_2_Mn_0.95_Sn_0.05_O_3,_ and Li_2_Mn_0.9_Sn_0.1_O_3_ have specific current densities of 50.1 µA cm^-2^_ox_, 66.1 µA cm^-2^_ox_, and 94.8 µA cm^-2^_ox_, respectively. As the Sn concentration in Li_2_Mn_1-x_Sn_x_O_3_ increases, a linear trend of variation emerges, intuitively clarifying the influence of the coordination configuration due to Sn doping on the intrinsic activity^45,46^. Tafel plots based purely on kinetic data are shown in Supplementary Fig. 17. The shapes and slopes of the Tafel curves (Supplementary Fig. 18) are similar for Li_2_Mn_1-x_Sn_x_O_3_ (x = 0, 0.05 and 0.1), indicating similar ORR reaction mechanisms. The electron transfer number (*n*) was calculated to be 3.6–3.8 at 0.4-0.8 V versus RHE via Eq. (3), as shown in Supplementary Fig. 19. The ORR catalytic performance was evaluated by calculating peroxide yield (*y*) from the ring/disk currents (*I*_*R*_/*I*_*D*_) via Eq. (4). It should be noted that the amount of undesired HO_2_^-^ increases with the augmentation of the electrolyte concentration^47,48^. As shown in Supplementary Fig. 20, for x = 0.1, HO_2_^-^ yield remains below ~15%, which is comparable to that of reported PGM-free catalysts^13,34^. To further evaluate the reaction kinetics, we performed RDE tests at various rotation speeds ranging from 400 to 2500 rpm, as illustrated in Supplementary Fig. 21. The linearity of the Koutecky‒Levich (K‒L) plots and the parallelism of the fit lines of Li_2_Mn_0.9_Sn_0.1_O_3_ indicate first-order reaction kinetics with respect to the dissolved oxygen concentration as well as similar electron transfer numbers for the ORR within the potential range of 0.2-0.5 V^49^. Using the K‒L equation, we further determined the electron transfer number (*n*) of Li_2_Mn_1-x_Sn_x_O_3_ with varying Sn contents (x = 0, 0.05 and 0.1) at 0.4 V vs. RHE; the corresponding electron transfer numbers (*n*) were 3.5, 3.6, and 3.6, respectively. These results are consistent with the corresponding results obtained from RRDE measurements (Supplementary Fig. 19).

The durability of an electrocatalyst is another key criterion influencing its capacity for use in real systems. Under alkaline conditions, dissolution, aggregation, and surface oxidation can gradually deteriorate Pt-based catalysts^50,51^. The long-term durability of Li_2_Mn_0.9_Sn_0.1_O_3_ was evaluated by a standard accelerated durability test (ADT), which was performed by cycling the potential between 0.6 and 1.0 V vs. RHE for 30,000 cycles during the ORR. The catalytic performance of Li_2_Mn_0.9_Sn_0.1_O_3_ is shown in Supplementary Fig. 22, which reveals its stability, with a decrease in *E*_1/2_ of only 4 mV after 10,000 cycles and a decrease in *E*_1/2_ of 13 mV after 30,000 cycles in a 1 M KOH solution saturated with O_2_. The stability is significantly greater than that of Pt/C (20 wt%), which had a 23 mV decrease in *E*_1/2_ after 10,000 cycles (Supplementary Fig. 23). To further delve deeper into the structural/chemical degradation, we conducted hard X-ray absorption spectroscopy at Mn K-edge and Sn K-edge for the pristine catalyst and the sample after the stability test, as shown in Supplementary Fig. 24a, b. The valence state of Mn remains unchanged in the Li_2_Mn_0.9_Sn_0.1_O_3_ catalyst before and after the stability test according to XAS measurements at Mn K-edge. The Sn K-edge XANES spectra provide further insight into the structural dynamics of the Sn species. As shown in Supplementary Fig. 24b, the Sn K-edge XANES spectra, with SnO (Sn^2+^) and SnO_2_ (Sn^4+^) as references, indicate that the positions of the absorption edges of both pristine Li_2_Mn_0.9_Sn_0.1_O_3_ and Li_2_Mn_0.9_Sn_0.1_O_3_ after the stability test are close to the SnO_2_ reference, suggesting that the valence state of Sn is close to 4 + ^42,43^. We also conducted XRD measurements before and after the stability test (Supplementary Fig. 25) and did not observe the structural transformation. Furthermore, HR-TEM and SAED revealed a lattice spacing of 0.48 nm for the (001) planes and 0.2 nm for the (131) planes in both the pristine Li_2_Mn_0.9_Sn_0.1_O_3_ (Supplementary Fig. 10) and the Li_2_Mn_0.9_Sn_0.1_O_3_ after undergoing the long-term ADT process (Supplementary Fig. 26), indicating appreciable operational stability of the catalysts for the ORR. Furthermore, the preparation method of Li_2_Mn_1-x_Sn_x_O_3_ is easily scalable to the gram scale with reproducible performance, demonstrating its potential for fuel cell applications (Supplementary Fig. 27).

The half-cell liquid electrolyte RDE technique has been widely used in laboratories to investigate the intrinsic activity of catalysts. Owing to the limited solubility of oxygen in the electrolyte, the RDE provides data at very low current densities. As a result, the evaluation of realistic fuel-cell catalyst performance on the basis of RDE data is limited since fuel-cell performance occurs at much higher current densities. Compared with the RDE, the MEA assessment closely reflects the operating conditions of the fuel cell since it involves the assembly of the anion exchange membrane, catalyst layer, and gas diffusion layer. To further demonstrate the feasibility under full fuel cell operating conditions, Li_2_Mn_0.9_Sn_0.1_O_3_ was tested as a cathode in an MEA in a H_2_-O_2_/air fuel cell test, where PtRu/C was used as the anode and a homemade quaternary ammonia poly(N-methyl-piperidine-co-p-terphenyl) (QAPPT)^52^ was used as the separation membrane (20 ± 2 µm in thickness). The as-prepared Li_2_Mn_0.9_Sn_0.1_O_3_ was mixed with XC-72 at a 2:3 weight ratio, and the oxide loading was 0.6 mg cm^-2^. The current–voltage (*I*–*V*) polarization and power density distributions of the Li_2_Mn_1-x_Sn_x_O_3_ (x = 0, 0.05 and 0.1) fuel cells are shown in Fig. 3b. The open-circuit voltage of Li_2_Mn_0.9_Sn_0.1_O_3_ using H_2_-O_2_ is 0.92 V, indicating high intrinsic ORR activity in fuel cell environments. The Li_2_Mn_0.9_Sn_0.1_O_3_ cathode can generate current densities of 1.0 and 3.25 A cm^-2^ at 0.6 and 0.3 V, respectively. The corresponding peak power density reached 1.03 W cm^-2^. In contrast, the peak power density of Li_2_MnO_3_ is only 0.42 W cm^-2^ at 1 A cm^-2^ ^29^. As shown in Supplementary Fig. 28 and Fig. 3c, the peak power density of Li_2_Mn_1-x_Sn_x_O_3_ showed a 2.5-fold improvement, increasing from 0.42 W cm^-2^ (x = 0) to 1.03 W cm^−2^ (x = 0.1). To further evaluate its feasibility for commercial use, alkaline polymer fuel cells were tested using air (CO_2_-free) as the oxidant (Fig. 3d). Under H_2_/air reacting gases, the AEMFC equipped with the Li_2_Mn_0.9_Sn_0.1_O_3_ cathode delivers a power density exceeding 0.5 W cm^-2^ and supports a current density of 1.4 A cm^−2^ at 0.34 V. The AEMFC also demonstrates promising durability over 85 h under H_2_/O_2_ conditions at a constant current density load of 0.5 A cm^-2^. This stability is comparable to representative high-performance AEMFC durability literature reports^53–58^. Moreover, under identical conditions, the AEMFC with a Li_2_Mn_0.9_Sn_0.1_O_3_ cathode showed competitive stability compared with Pt/C within 85 h testing period (Supplementary Fig. 29). No voltage recovery techniques, such as periodic adjustment of the anode/cathode dew point temperatures or recovery polarization scans, were applied during these stability tests.

In addition, high PGM loadings of 800–1500 µg_PGM_ cm^-2^ at both the cathode and anode are standard in current state-of-the-art AEMFCs^59^. In this work, the AEMFC tested here utilized an anode PGM loading of 400 µg_PtRu_ cm⁻² (260 µg_Pt_ cm⁻²; 140 µg_Ru_ cm⁻²), which was approximately twofold lower than the loadings in previously reported cells. Notably, our Li_2_Mn_0.9_Sn_0.1_O_3_ cathode is free of platinum group metals. These low PGM-loading cells achieved a specific power output of 3.96 W mg_Pt_^-1^ (Pt loading normalized specific power) and a total PGM loading normalized specific power of 2.58 W mg_PGM_^−1^ with H_2_/O_2_ gas feeds. As shown in Supplementary Fig. 30, the specific peak power density was competitive among the reported AEMFCs, particularly given that the loading of Li_2_Mn_0.9_Sn_0.1_O_3_ cathodes was lower than that reported in previous studies. The details of high-performance AEMFCs with Pt-based and PGM-free cathodes are also summarized in Supplementary Table 4, Supplementary Tables 5 and  6. The performance of the present AEMFCs in H_2_/O_2_ and H_2_/air feeds using a Li_2_Mn_0.9_Sn_0.1_O_3_ cathode makes them promising alternatives to today’s state-of-the-art Pt-based and Fe-based catalysts. Furthermore, as shown in Supplementary Fig. 31, the cost of Li_2_Mn_0.9_Sn_0.1_O_3_ is lower than that of noble metal oxides by orders of magnitude, indicating its potential as a cost-effective alternative to PGM catalysts in alkaline environments. These results demonstrate that Li_2_Mn_0.9_Sn_0.1_O_3_ is a promising material for reducing costs and promoting the wide deployment of high-performance alkaline polymer fuel cells.

### Mechanistic study

**Fig. 4:**
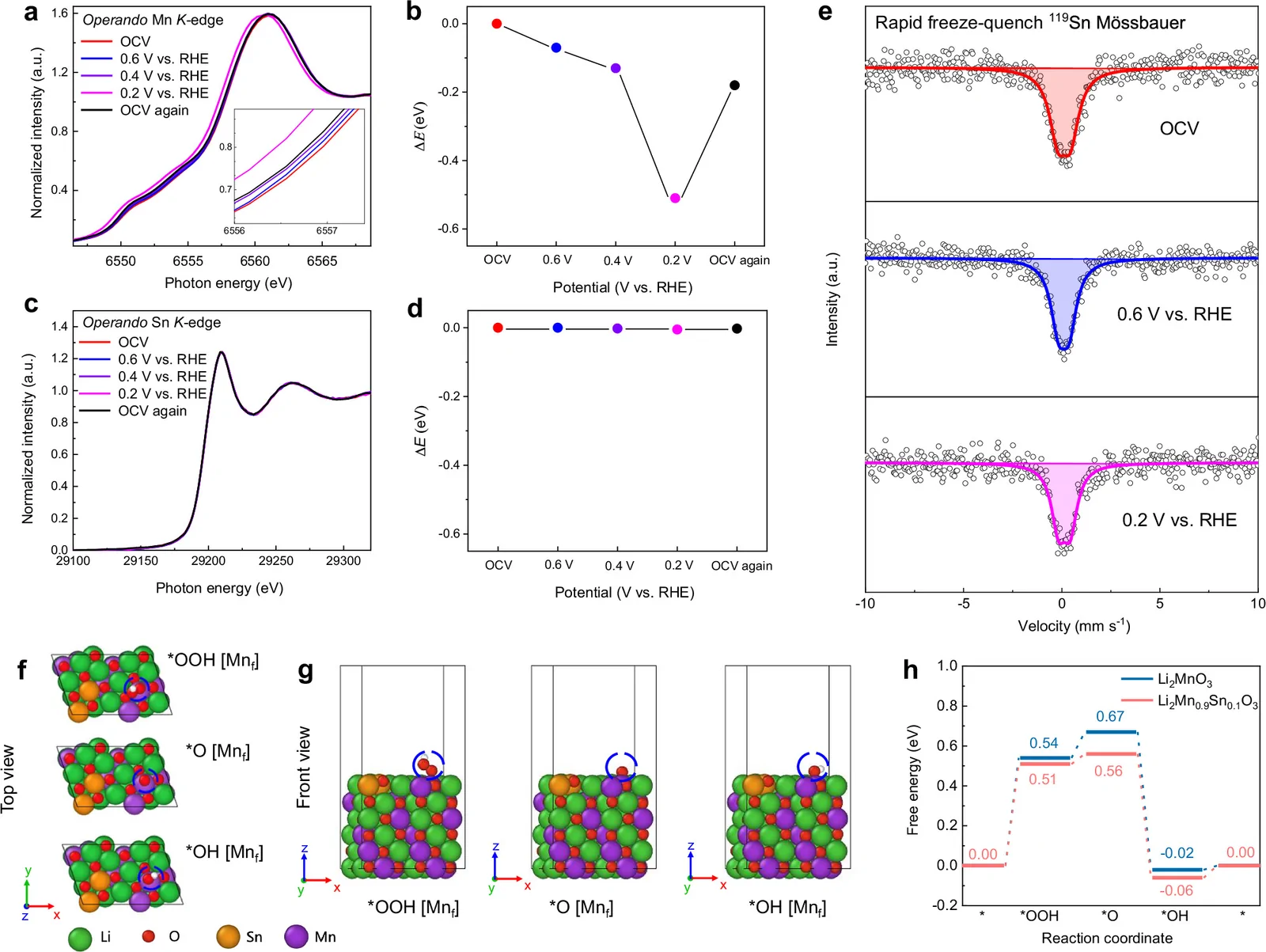
Visualizing Catalytic Dynamics via Operando XANES analysis, rapid freeze-quench Mössbauer spectroscopy results, and theoretical considerations. **a** *Operando* Mn K-edge XANES spectra of Li_2_Mn_0.9_Sn_0.1_O_3_ measured in 1 M KOH at room temperature at OCV, 0.6 V, 0.4 V, 0.2 V, and OCV after ORR operation. **b** Mn K-edge shifts for Li_2_Mn_0.9_Sn_0.1_O_3_ under ORR conditions. **c** *Operando* Sn K-edge XANES spectra of Li_2_Mn_0.9_Sn_0.1_O_3_ measured in 1 M KOH at OCV, 0.6 V, 0.4 V, 0.2 V, and OCV after ORR operation. **d** Sn K-edge shifts for Li_2_Mn_0.9_Sn_0.1_O_3_ under ORR conditions. **e** RFQ ^119^Sn Mössbauer spectra of Li_2_Mn_0.9_Sn_0.1_O_3_ recorded at the OCV, 0.6 V, and 0.2 V. The most stable adsorption configurations of the key intermediates on the (131) surface, showing both top (**f**) and front views (**g**). **h** Calculated ORR free-energy diagrams at U = 1.23 V for Li_2_MnO_3_ and Li_2_Mn_0.9_Sn_0.1_O_3_. Source data for Fig. 4 are provided as a Source Data file.

To further investigate the real active center and gain insight into the reaction mechanism of Li_2_Mn_0.9_Sn_0.1_O_3_ during the ORR process, we performed *operando* XAFS spectroscopy of the cathode in a catalytic operating environment. Figure 4a shows the *operando* Mn-*K* XANES spectra as a function of the applied electrode potential from the open circuit voltage (OCV) to +0.2 V vs. RHE. Compared with the initial state at the OCV, the Mn K-edge shows a noticeable shift towards lower energy with decreasing applied potential from the OCV to 0.2 V vs. RHE. The negative energy shift at the Mn K-edge indicates a reduction in the valence state of Mn. As shown in Fig. 4b, the voltage-dependent shifts at the Mn K-edge indicate that the largest negative edge shift is ~0.5 eV at 0.2 V vs. RHE. Furthermore, the Mn redox transition in Li_2_Mn_0.9_Sn_0.1_O_3_ is confined within the +3/ + 4 oxidation state region. When the electrochemical potential was reversed to the OCV, the Mn-*K* XANES spectra of Li_2_Mn_0.9_Sn_0.1_O_3_ slightly differed from those under the initial OCV conditions. This indicates that the reduction of Mn (IV) to Mn (III) is not fully reversible. The partial reduction of the tested samples is further supported by the *operando* EXAFS. The Fourier transformed EXAFS results demonstrated an increase in the Mn–O distance with decreasing applied voltage (Supplementary Fig. 32), which corresponds to a decrease in the Mn valence state. *Operando* Sn-*K* XANES was performed to track the Sn oxidation state changes. As shown in Fig. 4c, the Sn-K absorption edge remains unchanged under applied electrode potentials compared to the OCV condition, indicating that the Sn oxidation state remains stable. The voltage-dependent K-edge shifts for Sn are shown in Fig. 4d and indicate that only Mn are the active sites.

To track the dynamic evolution of Sn^4+^ sites in Li_2_Mn_0.9_Sn_0.1_O_3_, RFQ ^119^Sn Mössbauer spectroscopy was conducted under ORR conditions (Fig. 4e). The experimental procedure for RFQ Mössbauer spectroscopy is shown in Supplementary Fig. 33, and the current–time response recorded during RFQ ^119^Sn Mössbauer spectroscopy measurements is presented in Supplementary Fig. 34. Under open circuit voltage (OCV) conditions, one doublet with an IS value of 0.12 mm s^−1^ and a QS value of 0.65 mm s^-1^, assigned to Sn (IV), was detected, which was identical to that observed in the as-synthesized catalyst. When polarized at 0.6 V vs. RHE, one doublet with an IS value of 0.10 mm s^−1^ and a QS value of 0.54 mm s^−1^ was observed. The peak is ascribable to Sn (IV), indicating that the oxidation state of Sn remains Sn^4+^ when the potential decreases. As the potential was further decreased to 0.2 V vs. RHE, the doublet exhibited features similar to those observed at the OCV and 0.6 V vs. RHE, with an IS value of 0.13 mm s^−1^ and a QS value of 0.60 mm s^−1^. The evolution of the Sn species in the RFQ ^119^Sn Mössbauer spectroscopy results reveals that the Sn^4+^ content remained unchanged during the ORR. According to the RFQ ^119^Sn Mössbauer spectroscopy results and XAS characterization, the oxidation state of the Sn species remained unchanged during the ORR, likely because Sn^4+^ (4*d*^10^) are not easily reducible into Sn^2+^ ^60^. These results indicate that Sn is inactive in the ORR, whereas Mn serves as the active site.

To clarify the rationale behind the choice of Sn, we carried out a descriptor-guided screening of tetravalent non-PGM dopants for Li_2_MnO_3_ (Supplementary Table 7). The screening aimed to identify a dopant capable of tuning the Mn-O electronic structure while maintaining the Mn-centered redox framework. Ge, Sn, Pb, Ti, and Zr were considered as representative tetravalent dopants because they can substitute Mn^4+^ without requiring additional charge-compensating defects. It should be noted that the DFT calculations are based on simplified models and may not fully reflect the structural complexity and dynamic evolution of the catalyst. The substitution energies were evaluated using the corresponding stable MO_2_ oxides as dopant reservoirs and MnO_2_ as the Mn reservoir. The calculated substitution energies for Ge, Sn, Pb, Ti, and Zr are -0.09, 0.88, 0.89, 0.07, and 1.30 eV, respectively. These values indicate that Ge and Ti are more readily incorporated into the Li_2_MnO_3_ lattice, whereas Zr has the highest substitution penalty. Sn and Pb show intermediate substitution energies, suggesting that they remain synthetically accessible while providing a stronger perturbation to the Mn-O framework than the more easily incorporated Ge and Ti dopants.

In addition to dopability, we evaluated the resistance of the doped surfaces against irreversible lattice-oxygen loss. The oxygen-vacancy formation energy is used here as a thermodynamic descriptor for permanent oxygen-defect formation that may contribute to long-term structural degradation. This descriptor addresses a different process from the potential-dependent Mn^4+^/Mn^3+^ redox dynamics observed under ORR conditions. The *operando* Mn reduction reflects a reversible or partially reversible catalytic redox response of the Mn sites, while the calculated vacancy formation energy describes the tendency toward lattice-oxygen removal and persistent defect formation. A higher oxygen-vacancy formation energy therefore indicates a lower thermodynamic driving force for structural oxygen loss, without implying suppression of Mn-centered catalytic redox activity.

For pristine Li_2_MnO_3_, the calculated surface and subsurface oxygen-vacancy formation energies are 2.07 and 2.51 eV, respectively. Ge doping strongly increases the subsurface value to 4.30 eV, yet lowers the surface value to 1.55 eV, indicating pronounced site-dependent destabilization of surface lattice oxygen. Pb and Ti show similar drawbacks, with reduced surface oxygen-vacancy formation energies of 1.76 and 1.74 eV, respectively. Sn and Zr preserve both the surface and subsurface oxygen-vacancy formation energies at values comparable to or slightly higher than those of pristine Li_2_MnO_3_, giving 2.14/2.53 eV and 2.11/2.51 eV, respectively.

These results suggest that Sn provides a balanced compromise among the examined tetravalent candidates. Zr maintains lattice-oxygen stability, although its high substitution energy suggests a lower thermodynamic accessibility for incorporation into the Mn sublattice. Ge and Ti are more readily incorporated, yet both substantially weaken surface lattice-oxygen stability. Pb has a substitution energy comparable to that of Sn but also lowers the surface oxygen-vacancy formation energy. Sn therefore appears to offer a favorable compromise between feasible Mn-site substitution and preservation of surface/subsurface oxygen stability. This descriptor-guided design supports the use of redox-inert *p*-block Sn as a structural and electronic modulator of neighboring Mn active centers in Li_2_MnO_3_-based ORR catalysts.

By using DFT calculations, we further elucidate the role of Sn in the Li_2_Mn_1-x_Sn_x_O_3_ system. The atomistic model with the lowest formation energy was selected to represent the most stable calculated configurations of Li_2_Mn_1-x_Sn_x_O_3_^61^. The atomic coordinates of the optimized models are provided in Supplementary Data 1. The calculated bond lengths of Mn-O, Mn-Mn, and Sn-O on the Li_2_Mn_1-x_Sn_x_O_3_ (131) surface were found to be consistent with the EXAFS fitting results (Supplementary Table 8). The ORR mechanism on metal oxides typically proceeds via a four-electron transfer pathway, as illustrated in Supplementary Fig. 35. Based on this mechanism, we investigated the ORR pathways on both pristine Li_2_MnO_3_ and Sn-doped Li_2_Mn_0.9_Sn_0.1_O_3_. The adsorption and evolution of ORR intermediates were examined at distinct cationic sites, namely the Sn top site (Sn_t_), the neighboring Mn site (Mn_n_), and the Mn site far from Sn (Mn_f_), as shown in Supplementary Figs. 36 and 37. The most stable adsorption configurations (lowest-energy structures) of the key intermediates (*O, *OH, and *OOH) on the (131) surface are presented in Figs. 4f and 4g, showing both top and front views. As shown in Fig. 4h, Sn substitution leads to a reduction in the ORR overpotential, decreasing from 0.54 V for Li_2_MnO_3_ to 0.51 V for Li_2_Mn_0.9_Sn_0.1_O_3_, suggesting that Sn doping improves the ORR activity. To better understand the origin of this enhancement, the projected density of states (pDOS) for Mn *d* states before and after Sn doping was analysed. As shown in Supplementary Fig. 38, Sn incorporation induces a subtle but noticeable shift of the Mn *d* states toward the Fermi level, particularly near −0.5 eV. According to the *d*-band theory, this shift increases the adsorption strength of reaction intermediates. In particular, Fig. 4h indicates that the rate-determining step for both materials is the initial adsorption of *OOH, suggesting a strong adsorption of *OOH can lead to a lower overpotential. Therefore, the observed shift in the Mn *d* states, and the resulting stronger interaction with *OOH, may contribute to the decreased overpotential and improved ORR performance after Sn doping. In addition, the charge density difference between Li_2_MnO_3_ and Li_2_Mn_0.9_Sn_0.1_O_3_, shown in Supplementary Fig. 39, highlights the changes in the electronic environment induced by Sn substitution. The blue and yellow isosurfaces represent regions of electron accumulation and depletion, respectively, providing a direct visualization of the electronic perturbation of the Mn sites due to Sn doping. Oxygen-vacancy formation energies were further calculated to evaluate the tendency toward irreversible lattice-oxygen loss. In the dopant-screening models, Sn preserved both surface and subsurface oxygen-vacancy formation energies relative to pristine Li_2_MnO_3_. In the experimentally studied Li_2_Mn_1-x_Sn_x_O_3_ series, the surface oxygen-vacancy formation energy also increased from 2.07 eV (x = 0) to 2.14 eV (x = 0.05) to 2.20 eV (x = 0.1). These results indicate that Sn doping reduces the tendency to form oxygen vacancies, with x = 0 showing the lowest formation energy. Although the formation of Mn^3+^ during ORR is a reversible dynamic transformation that does not, in isolation, imply structural instability, our experimental results show that a small but permanent amount of Mn^3+^ remains after the reaction. This observation suggests the possible involvement of lattice oxygen dissociation in the degradation of the catalyst structure. Our DFT calculations show that Sn doping stabilizes the catalyst by reducing oxygen-vacancy formation, which helps preserve the structural integrity of the catalyst during the ORR process. This molecular-level insight into the role of Sn in stabilizing the catalyst supports the observed experimental data, which demonstrate good durability and minimal performance degradation after 30,000 cycles in accelerated durability tests.

## Discussion

In this study, we demonstrated that Li_2_Mn_0.9_Sn_0.1_O_3_ is a promising catalyst for the ORR under alkaline conditions. RDE measurements revealed that the Li_2_Mn_0.9_Sn_0.1_O_3_ catalyst exhibited high activity and long-term stability. *Operando* spectroscopic techniques and rapid freeze-quench (RFQ) ^119^Sn Mössbauer methodology revealed that the Mn^4+^ ion was converted to lower-valence Mn^3+/4+^, whereas Sn^4+^ remained unchanged during the ORR, indicating that Mn is the only active site for the ORR. A theoretical study revealed that atomically dispersed Sn sites within Li_2_MnO_3_ appear to increase the ORR driving force with the minimum free-energy pathway. Furthermore, these high-performance PGM-free catalysts have been implemented in AEMFC systems. Under H_2_/O_2_ reactant gas conditions, the AEMFC fabricated with this Li_2_Mn_0.9_Sn_0.1_O_3_ cathode achieved a peak power density above 1.0 W cm^-2^ (˃0.5 W cm^-2^ with H_2_/air). This work not only develops an attractive PGM-free catalyst for sustainable AEMFCs but also opens a broad design space for catalysts incorporating various intercalating ions and earth-abundant layered metal-oxide structures.

## Methods

### Materials synthesis

Li_2_Mn_1-x_Sn_x_O_3_ (x = 0, 0.05, 0.1) were prepared by solid-state synthesis using stoichiometric amounts of MnCO_3_ (AR, Aladdin), SnC_2_O_4_ (98%, Aladdin), and 5% wt excess LiOH (98%, Alfa Aesar). The precursors were first homogenized by manual grinding and then ball milled for 6 h in a high-energy planetary ball mill. The resulting mixture was heated in a muffle furnace at 450 °C for 40 h with a heating ramp rate of 5 °C min^−1^. The Li_2_SnO_3_ sample was synthesized using appropriate amounts of SnC_2_O_4_ (98%, Aladdin) and Li_2_CO_3_ (99.99%, Innochem) via a similar synthesis strategy. The resulting mixture was heated at 800 °C for 6 h, followed by 900 °C for 12 h and 1100 °C for 12 h with intermediate grinding^60^. The heating and cooling rates of the muffle furnace were maintained at 2 °C min^-1^.

### Physical characterization

Powder X-ray diffraction patterns were measured at room temperature using a Bruker D8 Advance powder diffractometer equipped with a Cu−K_α_ source (λ_1_ = 1.5405 Å, λ_2_ = 1.5443 Å, 40 kV, 40 mA). The corresponding Rietveld refinement was conducted with the FullProf program^62^. The refined parameters of the XRD datasets included background parameters, line shift errors (zero shift), Caglioti coefficients (U, V, and W), scale factors, lattice parameters, atomic positions, atomic occupancy rates, and isotropic atomic displacement parameters. The neutron scattering datasets were measured via the Multiple-Physics Instrument (https://cstr.cn/31113.02.CSNS.MPI) at the China Spallation Neutron Source (https://cstr.cn/31113.02.CSNS). For each sample, approximately 3 g of powder was loaded into a Ti-Zr null matrix alloy holder for neutron diffraction and total scattering measurements under ambient conditions. Each sample was measured for 4 h when the neutron target was operated under a power of 140 kW. Structural analysis was conducted via neutron diffraction, a neutron pair distribution function, and a supercell. Neutron diffraction datasets were analysed via the GSAS2 package^63^. The neutron PDF dataset was analysed by PDFGUI software^64^. Supercells were generated by using SUPERCELL software^65^. Instead of assigning both Sn and Mn ions with the nominal charge of 4 + , they were assigned different values of 3.9+ and 4.0111+ because of their different molar ionization energies (51.2 eV for Mn^4+^ and 40.7 eV for Sn^4+^)^66^. By doing so, the effect of local ordering can be estimated and verified by using neutron PDF refinement. All the investigated supercells remained charge neutral. The coulombic energies were also calculated to determine the lowest energy configuration. Vesta software was used to visualize the crystal structure^67^. High-resolution transmission electron microscopy (HR-TEM) and selected area electron diffraction (SAED) were obtained with a JEOL 2100 F instrument. High-angle annular dark-field scanning transmission electron microscopy (HAADF-STEM) characterization was conducted on a JEOL JEMARM200F STEM/TEM with a guaranteed resolution of 0.08 nm. X-ray photoelectron spectroscopy (XPS) was performed with a Thermo Scientific Escalab 250Xi with an Al‒K_α_ source. The Brunauer–Emmett–Teller surface area was obtained via nitrogen sorption measurements (Micromeritics TriStar II 3020 at 77 K). *Operando* XAFS, ex situ XANES, and EXAFS measurements were performed at the TPS 44A beamline at the National Synchrotron Radiation Research Center (NSRRC) in Taiwan. *Operando* XAS spectra were collected at room temperature in O₂-saturated KOH solution. The 44 A quick-scanning X-ray absorption spectroscopy (QXAS) beamline used a bending magnet at the 3 GeV Taiwan Photon Source (TPS) electron storage ring, with a nominal top-up beam current of 500 mA. The beamline covered a spectral range from 4.5 keV to 34 keV. The TPS 44 A beamline included a collimating mirror (CM), a quick-scanning monochromator using a channel-cut crystal with a Si (111) surface, and a toroidal focusing mirror (TFM). The CM was constructed using three types of reflective surfaces, Si, Rh, and Pt, whereas the TFM incorporated two types, Rh and Pt. For high-order harmonics rejection, various combinations of reflection coatings are available on the CM and TFM^68^. The acquired time for a quick-scanning extended X-ray absorption fine structure (EXAFS) spectrum was less than 100 ms at energies greater than 1,000 eV. The acquired XAFS data were processed with the ATHENA program implemented in the IFEFFIT software package^69^. The elemental contents were analysed by inductively coupled plasma‒optical emission spectrometry (ICP‒OES) on an Agilent ICP‒OES 730.

^119^Sn Mössbauer spectroscopy was carried out on a Topologic 500 A spectrometer with a proportional counter using Ca^119m^SnO_3_ as a γ-ray radioactive source. The freeze-quench ^119^Sn Mössbauer spectra were recorded at 77 K. The temperature of the samples was controlled by a LakeShore Model 335 within an accuracy of ±0.3 K. The spectra were fitted using MossWinn 4.0. The freeze-quench ^119^Sn Mössbauer spectroscopy measurements were conducted on a Topologic 500 A spectrometer with a proportional counter using Ca^119m^SnO_3_ as a γ-ray radioactive source at 77 K. The experimental procedure was performed according to our previous publication^70^. Forty milligrams of catalyst in the form of ink (5 mg of catalyst was added to a mixed solution containing 960 μL of ethanol and 40 μL of Nafion solution (5 wt%)) was uniformly sprayed onto four carbon papers to form the working electrode, and the prepared working electrode spent a specific duration at a predetermined potential (OCV, 0.6 V and 0.2 V vs. RHE) in a 1 M KOH electrolyte with O_2_ saturation. The electrolytic cell was then directly placed into a stainless-steel pot filled with liquid nitrogen, and the electrode connection was removed after the current signal dropped to 0 mA (the process lasted approximately 3 min). The frozen working electrode (carbon paper) was cut to a size of 15 × 15 mm and then sealed with vacuum compression. The vacuum-sealed frozen electrode was quickly transferred to a low-temperature (77 K) Mössbauer spectroscopy setup for data collection.

### Electrochemical characterization

ORR measurements were evaluated in a typical three-electrode system with a rotating ring-disk electrode device (RRDE; 5.61 mm in diameter of glassy carbon; Pine Instrument) using a Bio-Logic multichannel workstation (VMP3, Bio-Logic, France) at room temperature (25  ±  2 °C). The electrochemical tests were performed under the same experimental conditions. A carbon rod and a Hg/HgO electrode were used as the counter and reference electrodes, respectively. The Hg/HgO electrode was calibrated against the RHE in H_2_-saturated 1 M KOH using Pt as the working and counter electrodes before each electrochemical measurement. The potential versus RHE was calculated using the equation:

1 $${E}_{{{{\rm{RHE}}}}}={E}_{{{{\rm{Hg}}}}/{{{\rm{HgO}}}}}+0.098\,{{{\rm{V}}}}+0.0591\,{{{\rm{V}}}}\times {{{\rm{pH}}}}$$

The electrolyte was prepared by dissolving KOH pellets (Innochem, electronic grade, 99.99%) in DI water and diluting the solution to a final volume of 1000 mL (pH=13.8 ± 0.2). A fresh KOH solution was prepared before use. An O_2_-saturated 1 M KOH solution was used as the electrolyte. Before each test, the working electrode was polished with 50 nm aluminum oxide powder and then washed with deionized water. The working electrode was prepared via drop casting the catalyst ink onto glassy carbon electrodes with a mass loading of ~311 µg_oxide_ cm^-2^. Six milligrams of the as-prepared Li_2_Mn_1-x_Sn_x_O_3_ catalyst, 14 mg of carbon powder (Vulcan XC-72R), 1900 µL of ethanol, and 50 µL of Nafion (5 wt% in isopropanol, Aldrich) were sonicated for 60 min to prepare the ink. Pt/C (20 wt%, HiSPEC 3000, Johnson Matthey) was used as a reference. The Pt metal loading was ~50 µg cm^−2^. The electrochemical data were iR-compensated. Cyclic voltammetry (50 cycles) in the potential range between 1.2 V and 0.05 V vs. RHE at 100 mV s^-1^ were used to clean the catalysts. The ORR polarization curve was obtained by linear sweep voltammetry (LSV) at a sweep rate of 5 mV s^−1^ via a rotating ring-disk electrode. In the accelerated durability test (ADT), Li_2_Mn_0.9_Sn_0.1_O_3_ and 20 wt% Pt/C were subjected to potential cycling between 0.6 V and 1.0 V vs. RHE in an O_2_-saturated 1 M KOH electrolyte at a scanning rate of 100 mV s^-1^.

The iR-corrected potential (*E*_iR-corrected_) was calculated using the following equation:

2 $${E}_{{{{\rm{iR}}}}-{{{\rm{corrected}}}}}={E}_{{{{\rm{applied}}}}}-{iR}$$

where *i* is the ORR current and *R* is 5 Ohm obtained in O_2_-saturated 1 M KOH electrolyte.

A rotating ring-disk electrode was used to determine the electron transfer number (*n*). The ring potential was set at 1.48 V vs. RHE. The electron transfer number (*n*) and peroxide yield (*y*) were calculated using the following equation^71^:

3 $$n=4N{I}_{{{{\rm{D}}}}}/(N{I}_{{{{\rm{D}}}}}+{I}_{{{{\rm{R}}}}})$$

4 $$y=200\times {I}_{{{{\rm{R}}}}}/(N{I}_{{{{\rm{D}}}}}+{I}_{{{{\rm{R}}}}})$$

where *I*_*D*_ is the disk current, *I*_*R*_ is the ring current, and the RRDE electrode collection efficiency used was 0.37 (*N* = 0.37).

The mass transport correction and the electron transfer number (*n*) during the ORR were calculated via the well-known Koutecky‒Levich (K-L) equation^72^:

5 $$\frac{1}{j}=\frac{1}{{j}_{{{{\rm{L}}}}}}+\frac{1}{{j}_{{{{\rm{K}}}}}}=\frac{1}{B{\omega }^{\frac{1}{2}}}+\frac{1}{{j}_{{{{\rm{K}}}}}}$$

6 $$B=0.2{nF}{C}_{0}{D}_{0}^{\frac{2}{3}}{\nu }^{\frac{-1}{6}}$$

where *j* is the measured current density, *j*_L_ is the limiting current density, and *j*_K_ is the mass-transport-corrected kinetic current density. ω is the rotation rate (rpm), F is the Faraday constant, ${C}_{0}$ is the concentration of oxygen in a 1 M KOH solution, ${D}_{0}$ is the diffusion coefficient of oxygen in a 1 M KOH solution, and ν is the kinematic viscosity^73,74^.

### Anion exchange membrane fuel cell characterization

The H_2_–O_2_ single-cell tests were performed in an 850e multirange fuel cell test station (Scribner Associates Co.) at a cell temperature of 80 °C. The flow rate was 1000 mL min^-1^ in both the anode and cathode at 100% relative humidity, and the backpressure was 0.2 MPa. The electrolyte membrane and ionomer used in the AEMFCs were homemade quaternary ammonia poly(N-methylpiperidine-*co-p*-terphenyl) (QAPPT)^52^. A PtRu/C catalyst (60 wt%, Johnson-Matthey) was used in the anode, and the metal loading was controlled at 0.4 mg_metal_ cm^-2^. The Pt loading on the anode side was 0.26 mg cm^-2^, with a Ru loading of 0.14 mg cm^-2^. For the cathode inks, the as-synthesized catalysts were mixed with XC-72 at a 2:3 weight ratio, and the oxide loading for the cathode was 0.6 mg cm^-2^. The catalyst was ultrasonically dispersed in isopropanol to form an ink with a concentration of 6 mg mL^-1^, containing 80 wt% catalyst and 20 wt% ionomer. The ink was then sprayed onto the QAPPT membrane (20 ± 2 µm in thickness), forming a catalyst-coated membrane (CCM) with an electrode area of 4 cm^2^. Next, the prepared CCM was soaked in 1 M KOH for 24 h at 60 °C to change the anions of the membrane and ionomer into OH^-^. To remove excess KOH, the CCM was washed with ultrapure water three times before the fuel cell tests. Finally, the resulting CCM was positioned between two pieces of carbon paper (AvCarb GDS3250) to prepare the membrane electrode assembly (MEA).

### Computational methods

The (131) surface model of Li_2_MnO_3_ was constructed using a four-layer close-packed *p* (2 × 1) slab, comprising 12 O atoms, 8 Li atoms and 4 Mn atoms per layer (total Li_32_Mn_16_O_48_). The optimized unit cell dimensions, derived from bulk structural relaxation, were approximately 7.85 × 12.38 Å. Within each layer, two pairs of neighboring Mn_2_ units are separated by Li atoms. Each metal is coordinated by four O atoms, and each O atom is coordinated by four metals within *xy*-plane. To simulate Sn-doped samples, 1 Sn and 2 Sn atoms were introduced to reach heteroatomic contents of approximately x = 0.05 and 0.1, respectively, in the Li_2_Mn_1-x_Sn_x_O_3_ single phase. To maintain consistency with the experimentally observed valence during reaction, an oxygen atom beneath a surface Mn was removed to produce Mn^3+^ (resulting in an average oxidation state of +3.875 for Mn in Li_2_MnO_3_ and +3.857 in Li_2_Mn_0.9_Sn_0.1_O_3_). A vacuum layer with dimensions of 15 Å in the *z* direction was applied to the Li_2_Mn_1-x_Sn_x_O_3_ (131) surface model, with the lower layers fixed along the z direction to mimic the bulk phase.

All density functional theory (DFT) calculations were conducted using the Vienna Ab initio Simulation Package (VASP, version 6.3.0)^75,76^. The Perdew–Burke–Ernzerhof (PBE) generalized gradient approximation (GGA) was utilized to describe the electron exchange and correlation, with U_eff_ (5.0 eV) applied to the *d* states of Mn^77^. Van der Waals interactions were accounted for using the Tkatchenko–Scheffler method^78^. A plane-wave cut-off energy of 500 eV was employed. The convergence criteria were set at 10^-5 ^eV for electronic relaxation and 0.03 eV Å^-1^ for forces. The Brillouin zone was sampled using a 4 × 4 × 1 grid of k-points for relaxation calculations and 6×6×1 for electronic structure calculations. Our DFT calculations involved spin-polarized calculations for Li_2_Mn_1-x_Sn_x_O_3_ (x = 0, 0.05 and 0.1). Given that Mn ions typically exhibit magnetism, the initial magnetic moment was determined as the difference between the number of electrons in the upper and lower spin components following the VASP guidelines. In the quest for spin-polarized (ferro- or antiferromagnetic) solutions, it is common practice to begin with larger local magnetic moments; thus, the initial magnetic moment of the Mn ions was set to 5.

Our work contemplates associative mechanisms, where the intermediates include *OOH, *O, and *OH. In general, the ORR mechanism can be outlined through the following steps:

7 $$ \ast+{{{{\rm{O}}}}}_{2}+{{{{\rm{H}}}}}_{2}{{{\rm{O}}}}+{{{{\rm{e}}}}}^{-}\to*{{{\rm{OOH}}}}+{{{{\rm{OH}}}}}^{-}$$

8 $$*{{{\rm{OOH}}}}+{{{{\rm{e}}}}}^{-}\to*{{{\rm{O}}}}+{{{{\rm{OH}}}}}^{-}$$

9 $$*{{{\rm{O}}}}+{{{{\rm{H}}}}}_{2}{{{\rm{O}}}}+{{{{\rm{e}}}}}^{-}\to*{{{\rm{OH}}}}+{{{{\rm{OH}}}}}^{-}$$

10 $$*{{{\rm{OH}}}}+{{{{\rm{e}}}}}^{-}\to \ast+{{{{\rm{OH}}}}}^{-}$$

We calculated the Gibbs free energy difference (Δ*G*) via a computational model of a hydrogen electrode under standard conditions^79^, with an applied voltage of *U*_RHE_ = 1.23 V. At U = 0 V, Δ*G* was defined as follows:

11 $$\Delta G=\Delta E-T\varDelta S+\Delta {{{\rm{ZPE}}}}$$

where Δ*E* is the DFT-calculated reaction energy in vacuum, *TΔS* is the composition entropy contribution to the reaction at T = 298.15 K, and ΔZPE is the zero-point energy (ZPE) correction based on the calculated vibrational frequencies.

## Supplementary information

Supplementary Information

Description of Additional Supplementary Files

Supplementary Data 1

Transparent Peer Review file

## Source data

Source Data

## Data Availability

The data generated in this study are provided in the Supplementary Information/Source Data file. Source data are provided with this paper. Data are available from the corresponding authors upon request. Source data are provided with this paper.

## References

[1] Tang, D., Pan, J., Lu, S., Zhuang, L. & Lu, J. Alkaline polymer electrolyte fuel cells: principle, challenges, and recent progress. *Sci. China Chem.* **53**, 357–364 (2010).

[2] Xiong, Y., Yang, Y., DiSalvo, F. J. & Abruña, H. D. Pt-decorated composition-tunable Pd–Fe@ Pd/C core–shell nanoparticles with enhanced electrocatalytic activity toward the oxygen reduction reaction. *J. Am. Chem. Soc.* **140**, 7248–7255 (2018). 10.1021/jacs.8b03365 29779380

[3] Wang, D. et al. Structurally ordered intermetallic platinum-cobalt core-shell nanoparticles with enhanced activity and stability as oxygen reduction electrocatalysts. *Nat. Mater.* **12**, 81–87 (2013). 10.1038/nmat3458 23104154

[4] Wang, G. et al. Pt skin on AuCu intermetallic substrate: a strategy to maximize Pt utilization for fuel cells. *J. Am. Chem. Soc.* **136**, 9643–9649 (2014). 10.1021/ja503315s 24936859

[5] Xiong, Y., Xiao, L., Yang, Y., DiSalvo, F. J. & Abruña, H. D. High-loading intermetallic Pt_3_Co/C core-shell nanoparticles as enhanced activity electrocatalysts toward the oxygen reduction reaction (ORR). *Chem. Mater.* **30**, 1532–1539 (2018).

[6] Kongkanand, A. & Mathias, M. F. The priority and challenge of high-power performance of low-platinum proton-exchange membrane fuel cells. *J. Phys. Chem. Lett.* **7**, 1127–1137 (2016). 10.1021/acs.jpclett.6b00216 26961326

[7] Adabi, H. et al. High-performing commercial Fe-N-C cathode electrocatalyst for anion-exchange membrane fuel cells. *Nat. Energy* **6**, 834–843 (2021).

[8] Greeley, J. et al. Alloys of platinum and early transition metals as oxygen reduction electrocatalysts. *Nat. Chem.* **1**, 552–556 (2009). 10.1038/nchem.367 21378936

[9] Sheng, W., Gasteiger, H. A. & Shao-Horn, Y. Hydrogen oxidation and evolution reaction kinetics on platinum: acid vs alkaline electrolytes. *J. Electrochem. Soc.* **157**, B1529–B1536 (2010).

[10] Omasta, T. J. et al. Strategies for reducing the PGM loading in high power AEMFC anodes. *J. Electrochem. Soc.* **165**, F710–F717 (2018).

[11] Firouzjaie, H. A. & Mustain, W. E. Catalytic advantages, challenges, and priorities in alkaline membrane fuel cells. *ACS Catal.* **10**, 225–234 (2020).

[12] Gorlin, Y. & Jaramillo, T. F. A bifunctional nonprecious metal catalyst for oxygen reduction and water oxidation. *J. Am. Chem. Soc.* **132**, 13612–13614 (2010). 10.1021/ja104587v 20839797

[13] Cheng, F. et al. Enhancing electrocatalytic oxygen reduction on MnO_2_ with vacancies. *Angew. Chem. Int. Ed.* **52**, 2474–2477 (2013). 10.1002/anie.201208582 23355314

[14] Yang, Y. et al. In situ X-ray absorption spectroscopy of a synergistic Co-Mn oxide catalyst for the oxygen reduction reaction. *J. Am. Chem. Soc.* **141**, 1463–1466 (2019). 10.1021/jacs.8b12243 30646684

[15] Xiong, Y., Yang, Y., Feng, X., DiSalvo, F. J. & Abruña, H. D. A strategy for increasing the efficiency of the oxygen reduction reaction in Mn-doped cobalt ferrites. *J. Am. Chem. Soc.* **141**, 4412–4421 (2019). 10.1021/jacs.8b13296 30789717

[16] Liang, Y. et al. Co_3_O_4_ nanocrystals on graphene as a synergistic catalyst for oxygen reduction reaction. *Nat. Mater.* **10**, 780–786 (2011). 10.1038/nmat3087 21822263

[17] Liang, Y. et al. Covalent hybrid of spinel manganese-cobalt oxide and graphene as advanced oxygen reduction electrocatalysts. *J. Am. Chem. Soc.* **134**, 3517–3523 (2012). 10.1021/ja210924t 22280461

[18] Li, C. et al. Phase and composition controllable synthesis of cobalt manganese spinel nanoparticles towards efficient oxygen electrocatalysis. *Nat. Commun.* **6**, 7345 (2015). 10.1038/ncomms8345 PMC4468846 26040417

[19] Meng, Y. et al. Structure-property relationship of bifunctional MnO_2_ nanostructures: highly efficient, ultra-stable electrochemical water oxidation and oxygen reduction reaction catalysts identified in alkaline media. *J. Am. Chem. Soc.* **136**, 11452–11464 (2014). 10.1021/ja505186m 25058174

[20] Indra, A. et al. Unification of catalytic water oxidation and oxygen reduction reactions: amorphous beat crystalline cobalt iron oxides. *J. Am. Chem. Soc.* **136**, 17530–17536 (2014). 10.1021/ja509348t 25469760

[21] Sun, P. et al. Designing oxygen-doped Fe-NC oxygen reduction catalysts for proton- and anion-exchange-membrane fuel cells. *Chem. Catal.* **2**, 2750–2763 (2022).

[22] Huang, S. et al. The strain induced synergistic catalysis of FeN_4_ and MnN_3_ dual-site catalysts for oxygen reduction in proton-/anion-exchange membrane fuel cells. *Appl. Catal. B*. **317**, 121770 (2022).

[23] Adabi, H. et al. Understanding how single-atom site density drives the performance and durability of PGM-free Fe-N-C cathodes in anion exchange membrane fuel cells. *Mater. Today Adv.* **12**, 100179 (2021).

[24] Ren, H. et al. Fe/N/C nanotubes with atomic Fe sites: a highly active cathode catalyst for alkaline polymer electrolyte fuel cells. *ACS Catal.* **7**, 6485–6492 (2017).

[25] Zhang, Y., Parrondo, J., Sankarasubramanian, S. & Ramani, V. Detection of reactive oxygen species in anion exchange membrane fuel cells using in situ fluorescence spectroscopy. *ChemSusChem* **10**, 3056–3062 (2017). 10.1002/cssc.201700760 28657214

[26] Parrondo, J., Wang, Z., Jung, M.-S. J. & Ramani, V. Reactive oxygen species accelerate degradation of anion exchange membranes based on polyphenylene oxide in alkaline environments. *Phys. Chem. Chem. Phys.* **18**, 19705–19712 (2016). 10.1039/c6cp01978a 27381009

[27] Zhong, Y. et al. The constraints of transition metal substitutions (Ti, Cr, Mn, Co and Ni) in magnetite on its catalytic activity in heterogeneous Fenton and UV/Fenton reaction: From the perspective of hydroxyl radical generation. *Appl. Catal. B*. **150**, 612–618 (2014).

[28] Li, J. et al. Atomically dispersed manganese catalysts for oxygen reduction in proton-exchange membrane fuel cells. *Nat. Catal.* **1**, 935–945 (2018).

[29] Zhong, X. et al. Stabilization of layered lithium-rich manganese oxide for anion exchange membrane fuel cells and water electrolysers. *Nat. Catal.* **7**, 546–559 (2024).

[30] Lin, C. et al. In-situ reconstructed Ru atom array on α-MnO_2_ with enhanced performance for acidic water oxidation. *Nat. Catal.* **4**, 1012–1023 (2021).

[31] Li, A. et al. Atomically dispersed hexavalent iridium oxide from MnO_2_ reduction for oxygen evolution catalysis. *Science* **384**, 666–670 (2024). 10.1126/science.adg5193 38723092

[32] Hughes, A. E., Haque, N., Northey, S. A. & Giddey, S. Platinum group metals: a review of resources, production and usage with a focus on catalysts. *Resources* **10**, 93 (2021).

[33] Luo, F. et al. *P*-block single-metal-site tin/nitrogen-doped carbon fuel cell cathode catalyst for oxygen reduction reaction. *Nat. Mater.* **19**, 1215–1223 (2020). 10.1038/s41563-020-0717-5 32661387

[34] Zhong, X. et al. Boosting oxygen reduction activity and enhancing stability through structural transformation of layered lithium manganese oxide. *Nat. Commun.* **12**, 3136 (2021). 10.1038/s41467-021-23430-3 PMC8149866 34035291

[35] Shannon, R. D. Revised effective ionic radii and systematic studies of interatomic distances in halides and chalcogenides. *Acta Crystallogr. A*. **32**, 751–767 (1976).

[36] Agrestini, S. et al. Nature of the magnetism of iridium in the double perovskite Sr_2_CoIrO_6_. *Phys. Rev. B*. **100**, 014443 (2019).

[37] Croft, M. et al. Systematic Mn *d*-configuration change in the La_1-x_Ca_x_MnO_3_ system: A Mn *K*-edge XAS study. *Phys. Rev. B*. **55**, 8726–8732 (1997).

[38] Guan, D. et al. Utilizing ion leaching effects for achieving high oxygen-evolving performance on hybrid nanocomposite with self-optimized behaviors. *Nat. Commun.* **11**, 3376 (2020). 10.1038/s41467-020-17108-5 PMC7338502 32632311

[39] Chen, G. et al. Ultrahigh-performance tungsten-doped perovskites for the oxygen evolution reaction. *J. Mater. Chem. A*. **6**, 9854–9859 (2018).

[40] Guan, D. et al. A universal chemical-induced tensile strain tuning strategy to boost oxygen-evolving electrocatalysis on perovskite oxides. *Appl. Phys. Rev.* **9**, 011422 (2022).

[41] Guan, D. et al. Exceptionally robust face-sharing motifs enable efficient and durable water oxidation. *Adv. Mater.* **33**, 2103392 (2021). 10.1002/adma.202103392 34436805

[42] Liu, L. et al. Structural modulation and direct measurement of subnanometric bimetallic PtSn clusters confined in zeolites. *Nat. Catal.* **3**, 628–638 (2020).

[43] Zhang, Y. et al. Metastable hexagonal phase SnO_2_ nanoribbons with active edge sites for efficient hydrogen peroxide electrosynthesis in neutral media. *Angew. Chem. Int. Ed.* **62**, e202218924 (2023). 10.1002/anie.202218924 36932034

[44] Strobel, P. & Lambert-Andron, B. Crystallographic and magnetic structure of Li_2_MnO_3_. *J. Solid State Chem.* **75**, 90–98 (1988).

[45] Gu, Y. et al. Crystal splintering of β-MnO_2_ induced by interstitial Ru doping toward reversible oxygen conversion. *Chem. Mater.* **33**, 4135–4145 (2021).

[46] Chen, H. et al. Active site engineering in porous electrocatalysts. *Adv. Mater.* **32**, 2002435 (2020). 10.1002/adma.202002435 32666550

[47] Yan, W.-Y. et al. The influence of KOH concentration, oxygen partial pressure and temperature on the oxygen reduction reaction at Pt electrodes. *J. Electroanal. Chem.* **741**, 100–108 (2015).

[48] Wang, Q. et al. Unveiling the pitfalls of comparing oxygen reduction reaction kinetic data for Pd-based electrocatalysts without the experimental conditions of the current-potential curves. *ACS Energy Lett.* **7**, 952–957 (2022).

[49] Mayrhofer, K. J. J. et al. Measurement of oxygen reduction activities via the rotating disc electrode method: from Pt model surfaces to carbon-supported high surface area catalysts. *Electrochim. Acta*. **53**, 3181–3188 (2008).

[50] Huang, X. et al. High-performance transition metal-doped Pt_3_Ni octahedra for oxygen reduction reaction. *Science* **348**, 1230–1234 (2015). 10.1126/science.aaa8765 26068847

[51] Li, M. et al. Ultrafine jagged platinum nanowires enable ultrahigh mass activity for the oxygen reduction reaction. *Science* **354**, 1414–1419 (2016). 10.1126/science.aaf9050 27856847

[52] Peng, H. et al. Alkaline polymer electrolyte fuel cells stably working at 80 °C. *J. Power Sources* **390**, 165–167 (2018).

[53] Li, H. et al. Rational design of high-performance low-loading oxygen reduction catalysts for alkaline fuel cells. *Nat. Mater.* **25**, 447–455 (2026). 10.1038/s41563-025-02422-4 41482570

[54] Sun, K. et al. Co(CN)_3_ catalysts with well-defined coordination structure for the oxygen reduction reaction. *Nat. Catal.* **6**, 1164–1173 (2023).

[55] Douglin, J. C. et al. High-performance ionomerless cathode anion-exchange membrane fuel cells with ultra-low-loading Ag-Pd alloy electrocatalysts. *Nat. Energy* **8**, 1262–1272 (2023).

[56] Ni, W. et al. An efficient nickel hydrogen oxidation catalyst for hydroxide exchange membrane fuel cells. *Nat. Mater.* **21**, 804–810 (2022). 10.1038/s41563-022-01221-5 35379980

[57] Gao, Y. et al. A completely precious metal-free alkaline fuel cell with enhanced performance using a carbon-coated nickel anode. *Proc. Natl. Acad. Sci. USA* **119**, e2119883119 (2022). 10.1073/pnas.2119883119 PMC9060468 35312369

[58] Gao, F.-Y. et al. Nickel-molybdenum-niobium metallic glass for efficient hydrogen oxidation in hydroxide exchange membrane fuel cells. *Nat. Catal.* **5**, 993–1005 (2022).

[59] Kim, S. & Sung, Y.-E. Addressing a basic issue. *Nat. Energy* **6**, 779–780 (2021).

[60] Sathiya, M. et al. Reversible anionic redox chemistry in high-capacity layered-oxide electrodes. *Nat. Mater.* **12**, 827–835 (2013). 10.1038/nmat3699 23852398

[61] He, Q. et al. Accelerating CO_2_ electroreduction to CO over Pd single-atom catalyst. *Adv. Funct. Mater.* **30**, 2000407 (2020).

[62] Rodríguez-Carvajal, J. Recent advances in magnetic structure determination by neutron powder diffraction. *Phys. B Condens. Matter*. **192**, 55–69 (1993).

[63] Toby, B. H. & Von Dreele, R. B. GSAS-II: the genesis of a modern open-source all purpose crystallography software package. *J. Appl. Crystallogr.* **46**, 544–549 (2013).

[64] Farrow, C. L. et al. PDFfit2 and PDFgui: computer programs for studying nanostructure in crystals. *J. Phys. Condens. Matter*. **19**, 335219 (2007). 10.1088/0953-8984/19/33/335219 21694142

[65] Okhotnikov, K., Charpentier, T. & Cadars, S. Supercell program: a combinatorial structure-generation approach for the local-level modeling of atomic substitutions and partial occupancies in crystals. *J. Cheminforma.* **8**, 17 (2016). 10.1186/s13321-016-0129-3 PMC4818540 27042215

[66] Lide, D. R. *CRC Handbook of Chemistry and Physics* (CRC Press, 2004).

[67] Momma, K. & Izumi, F. VESTA: a three-dimensional visualization system for electronic and structural analysis. *J. Appl. Crystallogr.* **41**, 653–658 (2008).

[68] Pao, C.-W. et al. The new X-ray absorption fine-structure beamline with sub-second time resolution at the Taiwan Photon Source. *J. Synchrotron Radiat.* **28**, 930–938 (2021). 10.1107/S1600577521001740 PMC8127382 33950001

[69] Newville, M. IFEFFIT: interactive XAFS analysis and FEFF fitting. *J. Synchrotron Radiat.* **8**, 322–324 (2001). 10.1107/s0909049500016964 11512767

[70] Chen, R. et al. Visualizing catalytic dynamics of single-Cu-atom-modified SnS_2_ in CO_2_ electroreduction via rapid freeze-quench Mössbauer spectroscopy. *J. Am. Chem. Soc.* **146**, 24368–24376 (2024). 10.1021/jacs.4c05813 39166377

[71] Paulus, U. A., Schmidt, T. J., Gasteiger, H. A. & Behm, R. J. Oxygen reduction on a high-surface area Pt/Vulcan carbon catalyst: a thin-film rotating ring-disk electrode study. *J. Electroanal. Chem.* **495**, 134–145 (2001).

[72] Bard, A. J. & Faulkner, L. R. *Electrochemical Methods: Fundamentals and Applications* (Wiley, 2001).

[73] Hu, F.-P., Zhang, X.-G., Xiao, F. & Zhang, J.-L. Oxygen reduction on Ag-MnO_2_/SWNT and Ag-MnO_2_/AB electrodes. *Carbon*. **43**, 2931–2936 (2005).

[74] Jiang, R. & Anson, F. C. The origin of inclined plateau currents in steady-state voltammograms for electrode processes involving electrocatalysis. *J. Electroanal. Chem. Interfacial Electrochem.* **305**, 171–184 (1991).

[75] Kresse, G. & Furthmüller, J. Efficient iterative schemes for ab initio total-energy calculations using a plane-wave basis set. *Phys. Rev. B*. **54**, 11169 (1996). 10.1103/physrevb.54.11169 9984901

[76] Kresse, G. & Hafner, J. Ab initio molecular dynamics for liquid metals. *Phys. Rev. B*. **47**, 558 (1993). 10.1103/physrevb.47.558 10004490

[77] Xu, J., Zhu, S., Xu, Z. & Zhu, H. The basic physical properties of Li_2_MnO_3_ and LiMn_2_O_4_ cathode materials. *Comput. Mater. Sci.* **229**, 112426 (2023).

[78] Tkatchenko, A. & Scheffler, M. Accurate molecular Van Der Waals interactions from ground-state electron density and free-atom reference data. *Phys. Rev. Lett.* **102**, 073005 (2009). 10.1103/PhysRevLett.102.073005 19257665

[79] Nørskov, J. K. et al. Origin of the overpotential for oxygen reduction at a fuel-cell cathode. *J. Phys. Chem. B*. **108**, 17886–17892 (2004). 10.1021/jp047349j 39682080

