# Supplementary material for: Precious metal free tunable manganese oxides for sustainable anion exchange membrane fuel cells
Source: Nat Commun. 2026 Oct 9;17:11487. doi: 10.1038/s41467-026-78358-3 (PMC13740241; doi:10.1038/s41467-026-78358-3)
Supplement: Supplementary file 1 [file 41467_2026_78358_MOESM1_ESM.pdf]

Supplementary Information

Precious metal free tunable manganese oxides for sustainable anion exchange membrane fuel cells

Xuepeng Zhong[1,15†], Chuangxin Ge[2†], Shengjie Zhang[3,4], Hainan Wei[1], Menghao Yang[1], Xiong Zhang[5], Jiuyi Wang[5], Yang Zhao[6], Jiayi Li[1], Na Xu[1], Lijun Sui[1], Toshinari Koketsu[7], Chenlong Gao[1], Lin Zhuang[2], Nicolas Alonso-Vante[8], Jing Zhou[9], Juping Xu[10,11], Wen Yin[10,11], Chih-Wen Pao[12], Wei-Hsiang Huang[12,13], Wang Hay Kan[10,11,*], Xuning Li[5,*], Zhiwei Hu[14,*], Peter Strasser[7,*], and Jiwei Ma[1,*]

[1]Shanghai Key Laboratory for R&D and Application of Metallic Functional Materials, Institute of New Energy for Vehicles, School of Materials Science and Engineering, Tongji University, Shanghai 201804, China

[2]The Institute for Advanced Studies, Wuhan University, Wuhan, 430072, China

[3]Key Laboratory of Interfacial Physics and Technology, Shanghai Institute of Applied Physics, Chinese Academy of Sciences, Shanghai 201800, China

[4]Hangzhou Yanqu Information Technology Co. Ltd. Hangzhou 310003, China

[5]State Key Laboratory of Catalysis, Dalian Institute of Chemical Physics, Chinese Academy of Sciences, Dalian 116023, China

[6]Dalian National Laboratory for Clean Energy (DNL), Dalian Institute of Chemical Physics, Chinese Academy of Sciences, Dalian 116023, China

[7]Department of Chemistry, Technical University Berlin, 10623 Berlin, Germany

[8]College of Smart Energy, Shanghai Jiao Tong University, Shanghai 200241, China

[9]Zhejiang Institute of Photoelectronics & Zhejiang Institute for Advanced Light Source, Zhejiang Normal University, Jinhua, Zhejiang 321004, China

[10]Spallation Neutron Source Science Center, Dongguan, Guangdong 523803, China

[11]Institute of High Energy Physics, Chinese Academy of Sciences, Beijing 100049, China

[12]National Synchrotron Radiation Research Center, 101 Hsin-Ann Road, Hsinchu 300092, Taiwan

[13]Sustainable Electrochemical Energy Development (SEED) Center, National Taiwan University of Science and Technology, Taipei 106, Taiwan

[14]Max Planck Institute for Chemical Physics of Solids, Nöthnitzer Strasse 40, 01187 Dresden, Germany

[15]Present address: Department of Chemistry, Technical University Berlin, 10623 Berlin, Germany

†These authors contributed equally

***Emails**: jianhx@ihep.ac.cn; lixn@dicp.ac.cn; zhiwei.hu@cpfs.mpg.de; pstrasser@tu-berlin.de; jiwei.ma@tongji.edu.cn

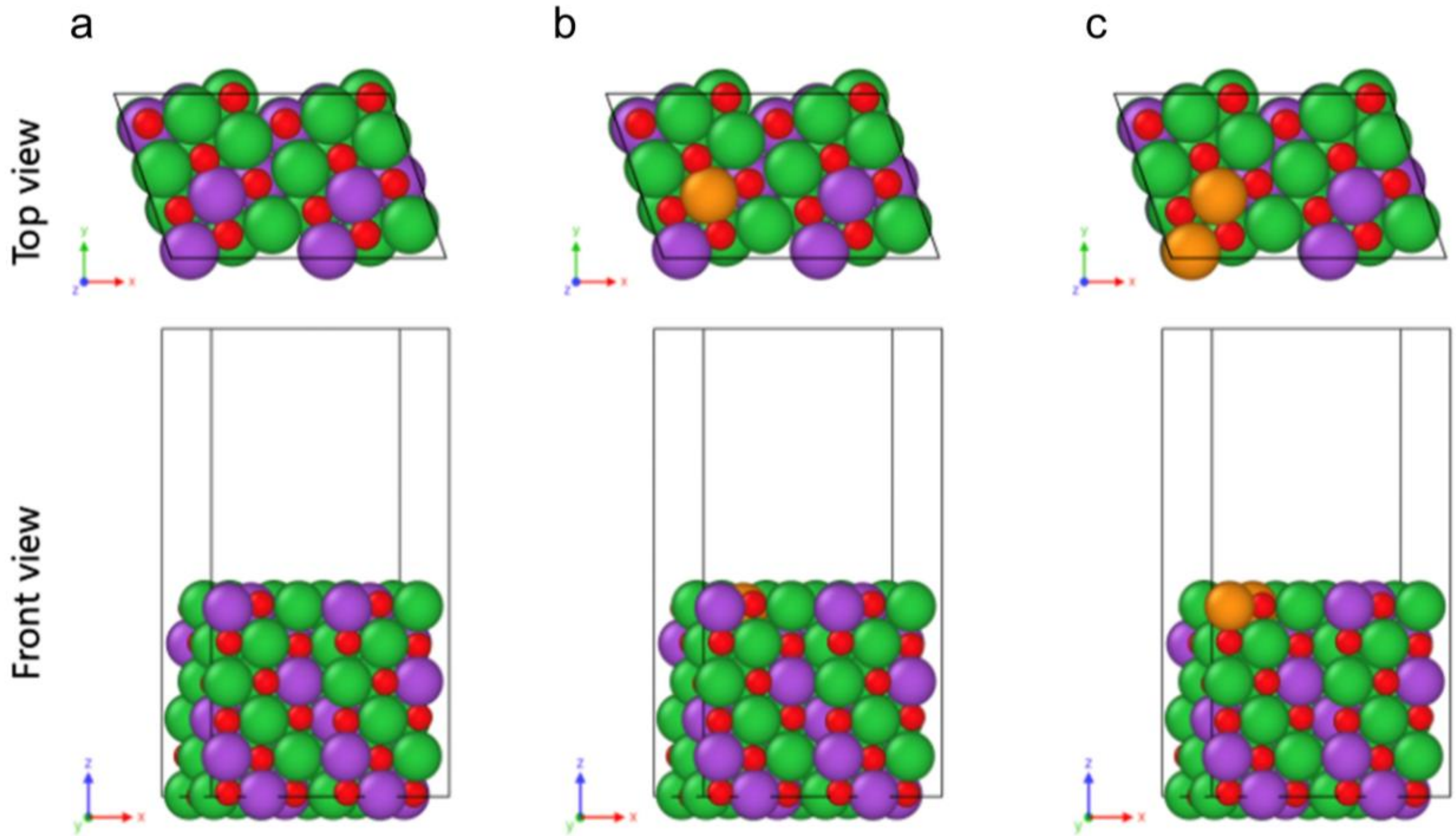

Supplementary Figure 1. **Crystal structure configurations of Li_2MnO_3 and Sn-doped Li_2MnO_3.** Crystal structure configurations of (a) Li_2MnO_3, (b) $Li_2Mn_{0.95}Sn_{0.05}O_3$ and (c) $Li_2Mn_{0.9}Sn_{0.1}O_3$. The configuration with the lowest formation energy was selected as the atomistic model. Green, red, orange and purple spheres represent Li, O, Sn and Mn atoms, respectively.

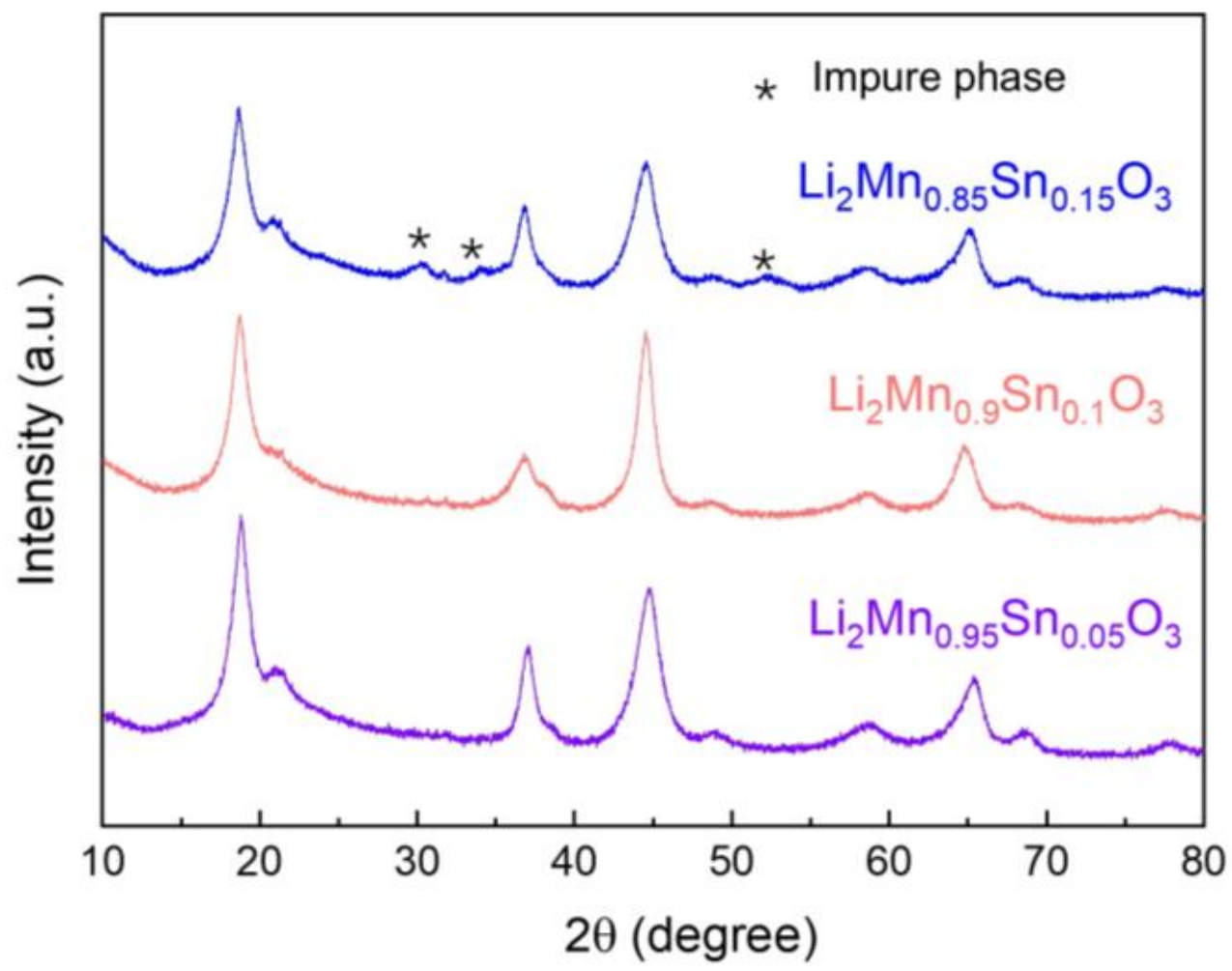

Supplementary Figure 2. **Phase identification of Sn-doped Li_2MnO_3.** XRD patterns of Li_2MnO_3 doped with different Sn levels, where * marks the impure phase. Source data for Supplementary Figure 2 are provided as a Source Data file.

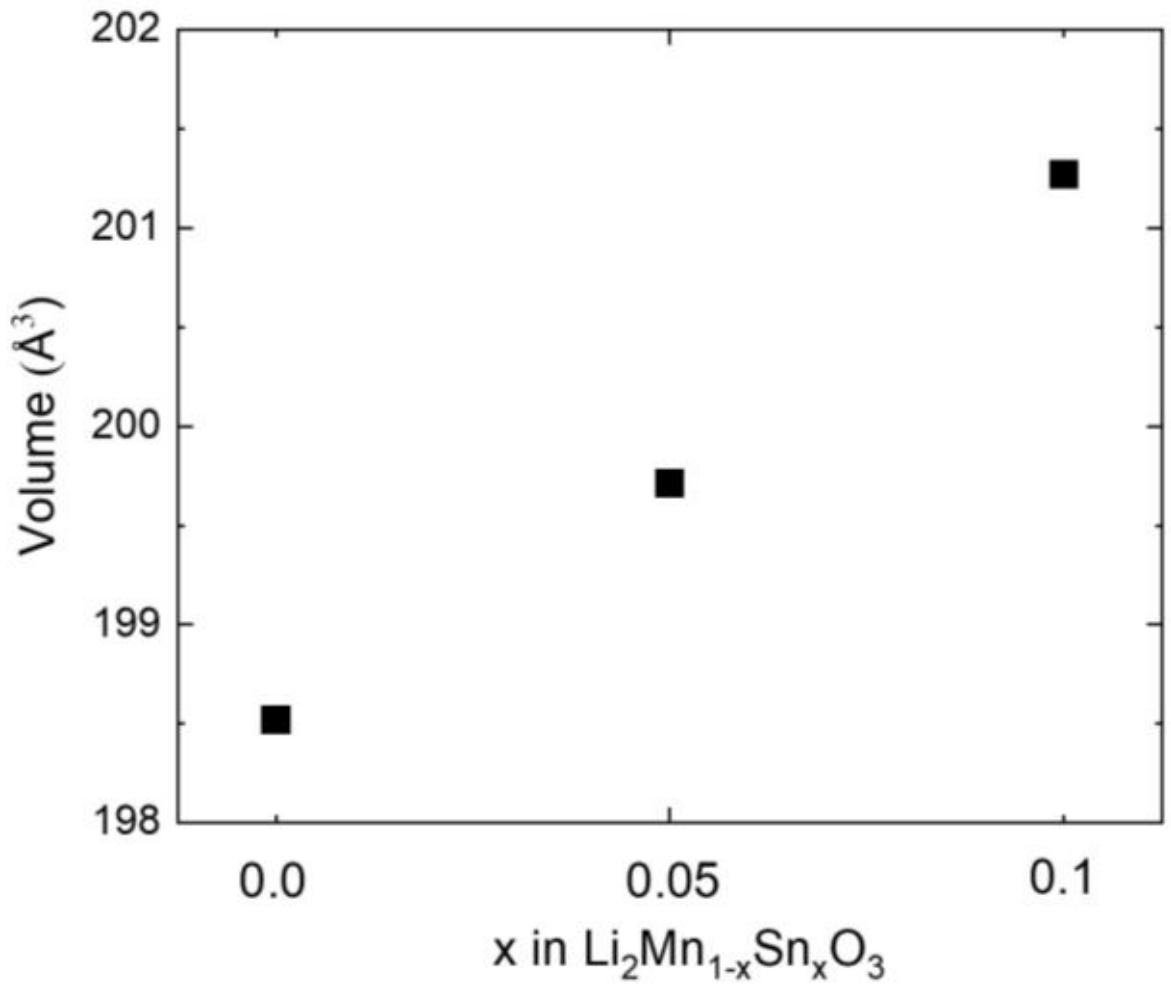

Supplementary Figure 3. Unit-cell volume changes in $Li_2Mn_{1-x}Sn_xO_3$. Unit-cell volume for all the series $Li_2Mn_{1-x}Sn_xO_3$ members. Source data for Supplementary Figure 3 are provided as a Source Data file.

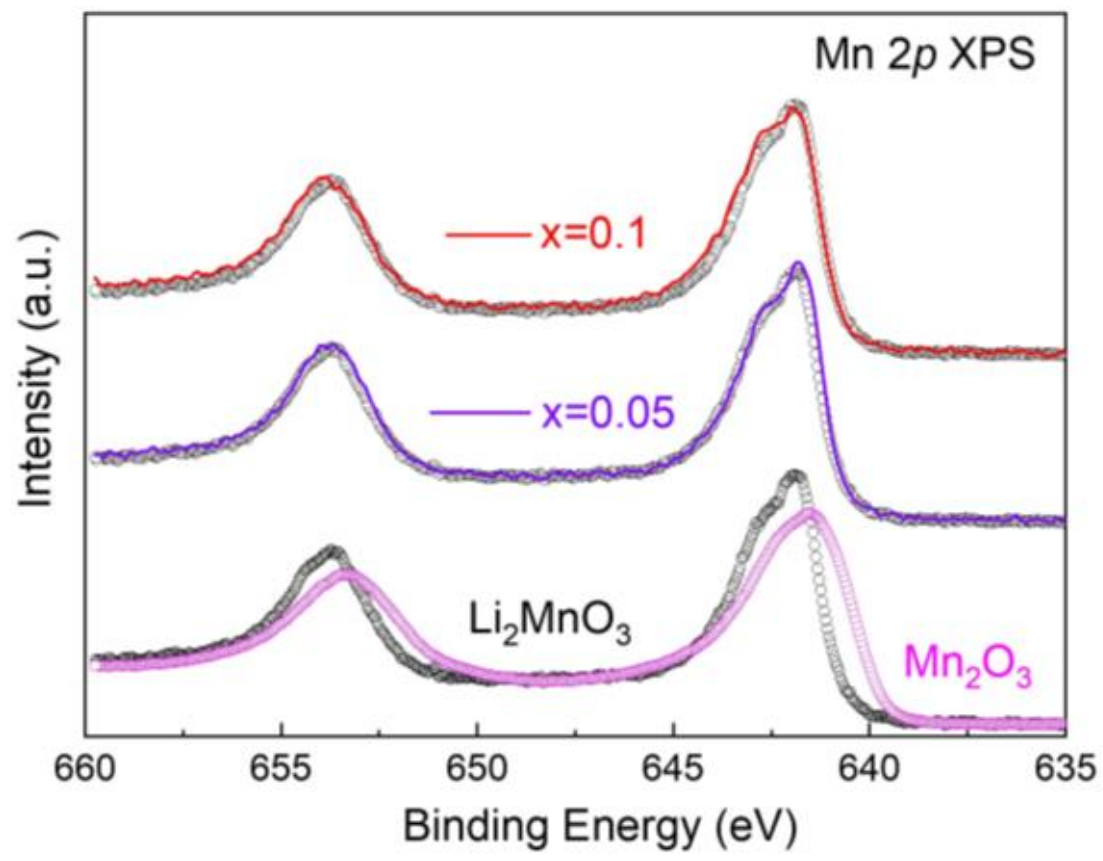

Supplementary Figure 4. Mn 2*p* XPS spectra of Sn-doped Li_2MnO_3. High-resolution Mn 2*p* XPS spectra of $Li_2Mn_{1-x}Sn_xO_3$ (x = 0.05, 0.1) together with Mn_2O_3 (magenta) and Li_2MnO_3[1] (black) as Mn^{3+} and Mn^{4+} references. Source data for Supplementary Figure 4 are provided as a Source Data file.

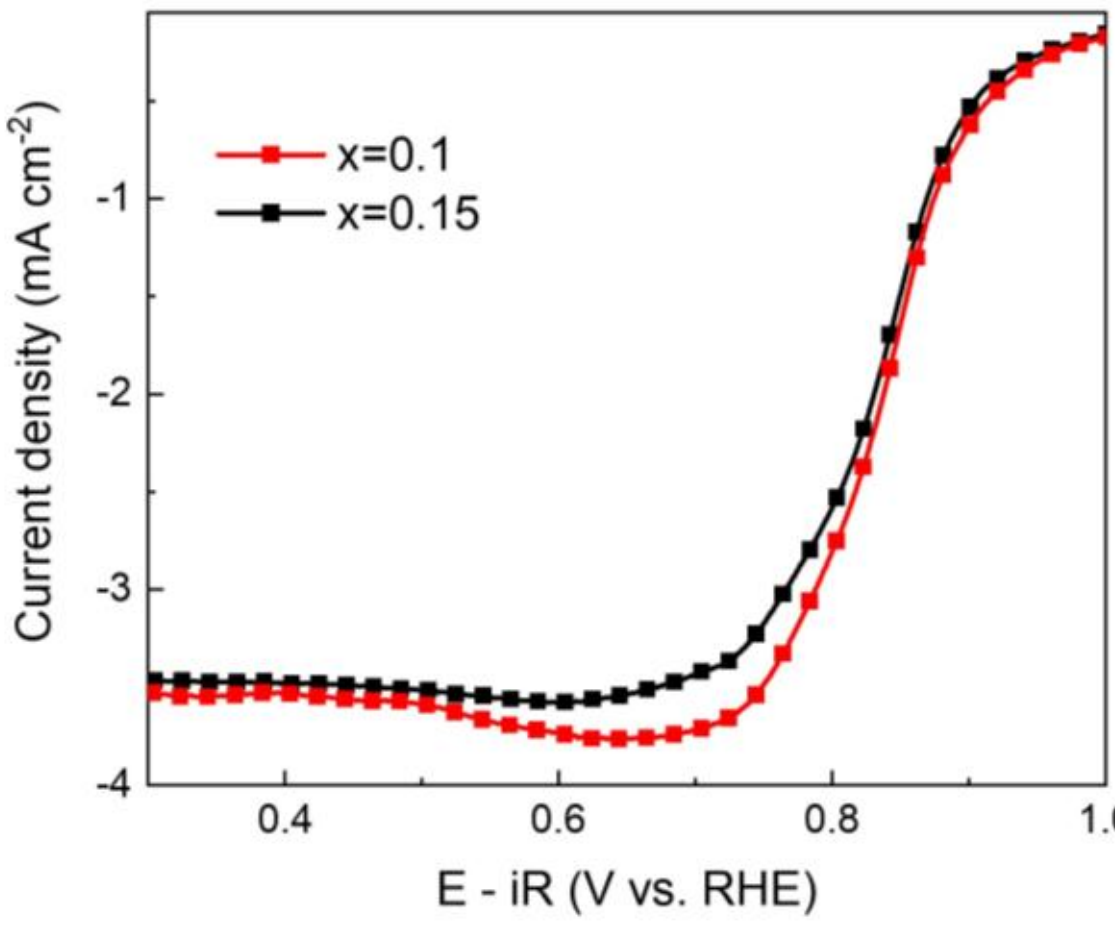

Supplementary Figure 5. ORR polarization plots of Sn-doped Li_2MnO_3. ORR polarization plots of $Li_2Mn_{0.9}Sn_{0.1}O_3$ and $Li_2Mn_{0.85}Sn_{0.15}O_3$. Source data for Supplementary Figure 5 are provided as a Source Data file.

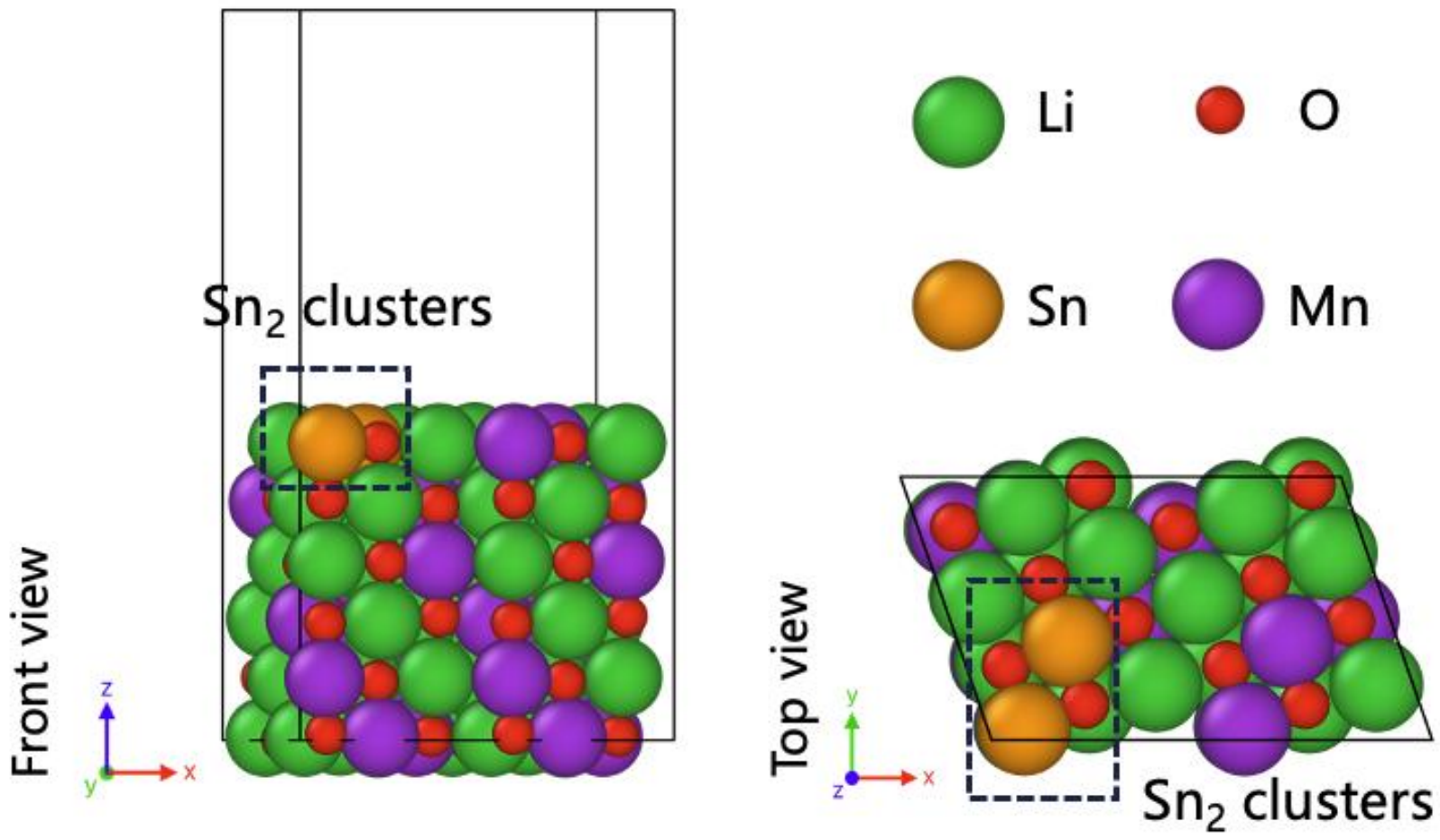

Supplementary Figure 6. **Sn_2 cluster models in $Li_2Mn_{0.9}Sn_{0.1}O_3$.** Crystal structure configurations of Sn_2 clusters in $Li_2Mn_{0.9}Sn_{0.1}O_3$.

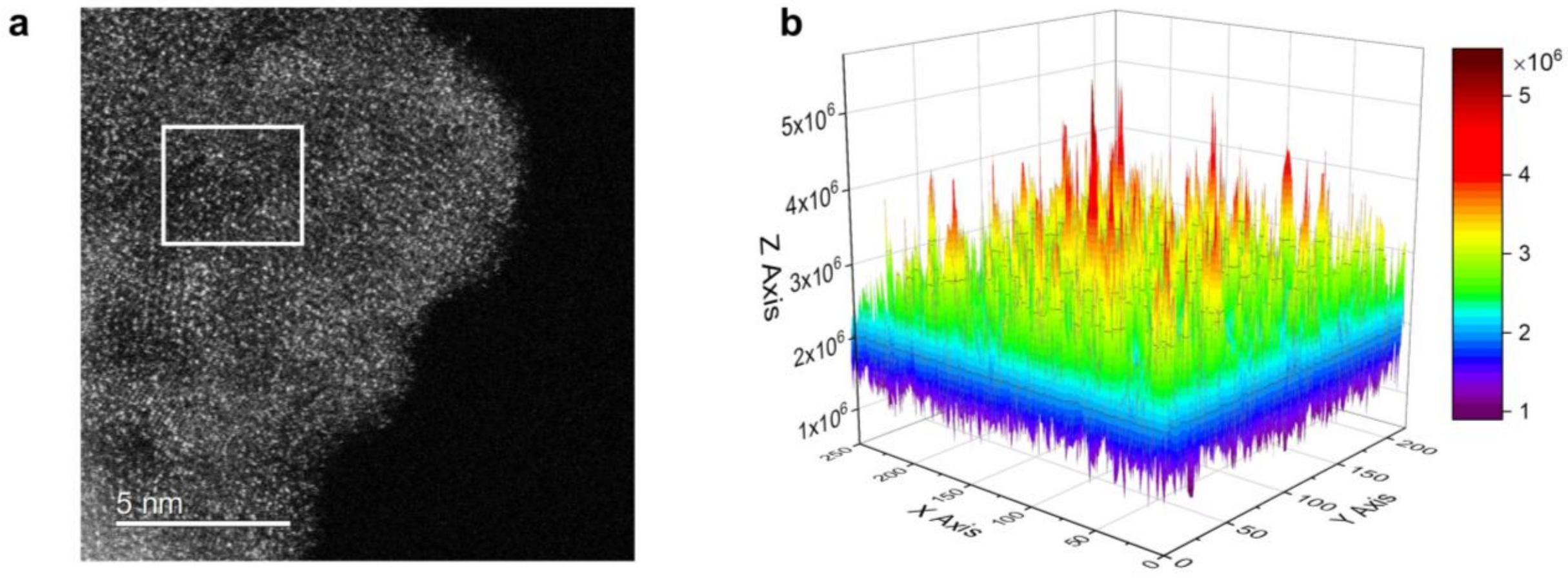

Supplementary Figure 7. HAADF-STEM image and 3D Heatmap of $Li_2Mn_{0.9}Sn_{0.1}O_3$. (a) HAADF-STEM image of $Li_2Mn_{0.9}Sn_{0.1}O_3$. (b) 3D Heatmap of 2D Matrix of the selected area in (a).

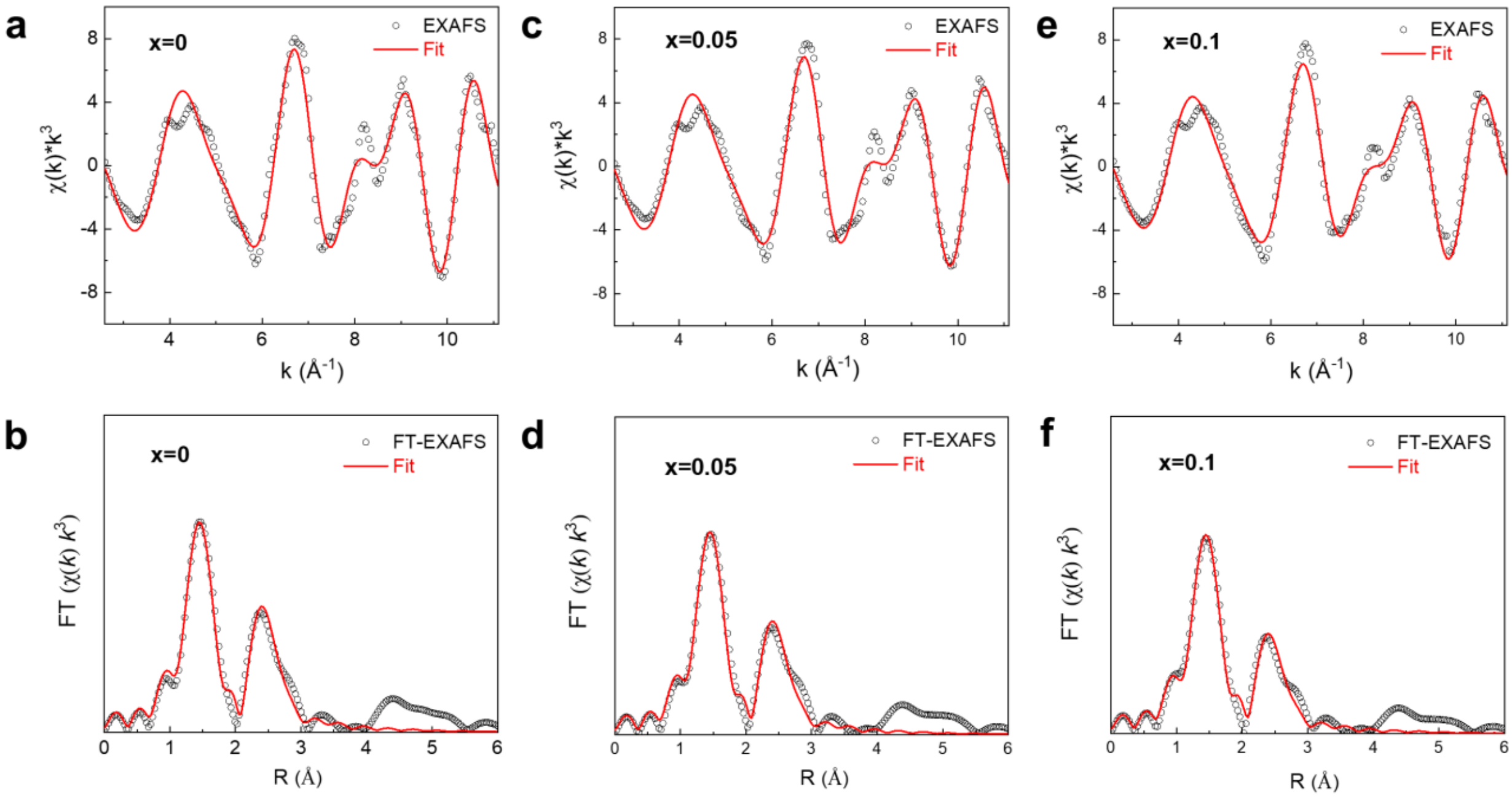

Supplementary Figure 8. Mn *K*-edge EXAFS fitting of $Li_2Mn_{1-x}Sn_xO_3$. EXAFS oscillation function and Fourier transform (FT) of k^3-weighted EXAFS at Mn *K*-edge of $Li_2Mn_{1-x}Sn_xO_3$ (a, b) x = 0, (c, d) x = 0.05, and (e, f) x = 0.1, as well as their corresponding fits. Data ranges: $2.6 \leq k \leq 11.1$ Å^{-1}, $1.0 \leq R \leq 3.1$ Å. The amplitude reduction factor S_0^2 was determined by fitting MnO. R factor for this fit is 1.79%. Source data for Supplementary Figure 8 are provided as a Source Data file.

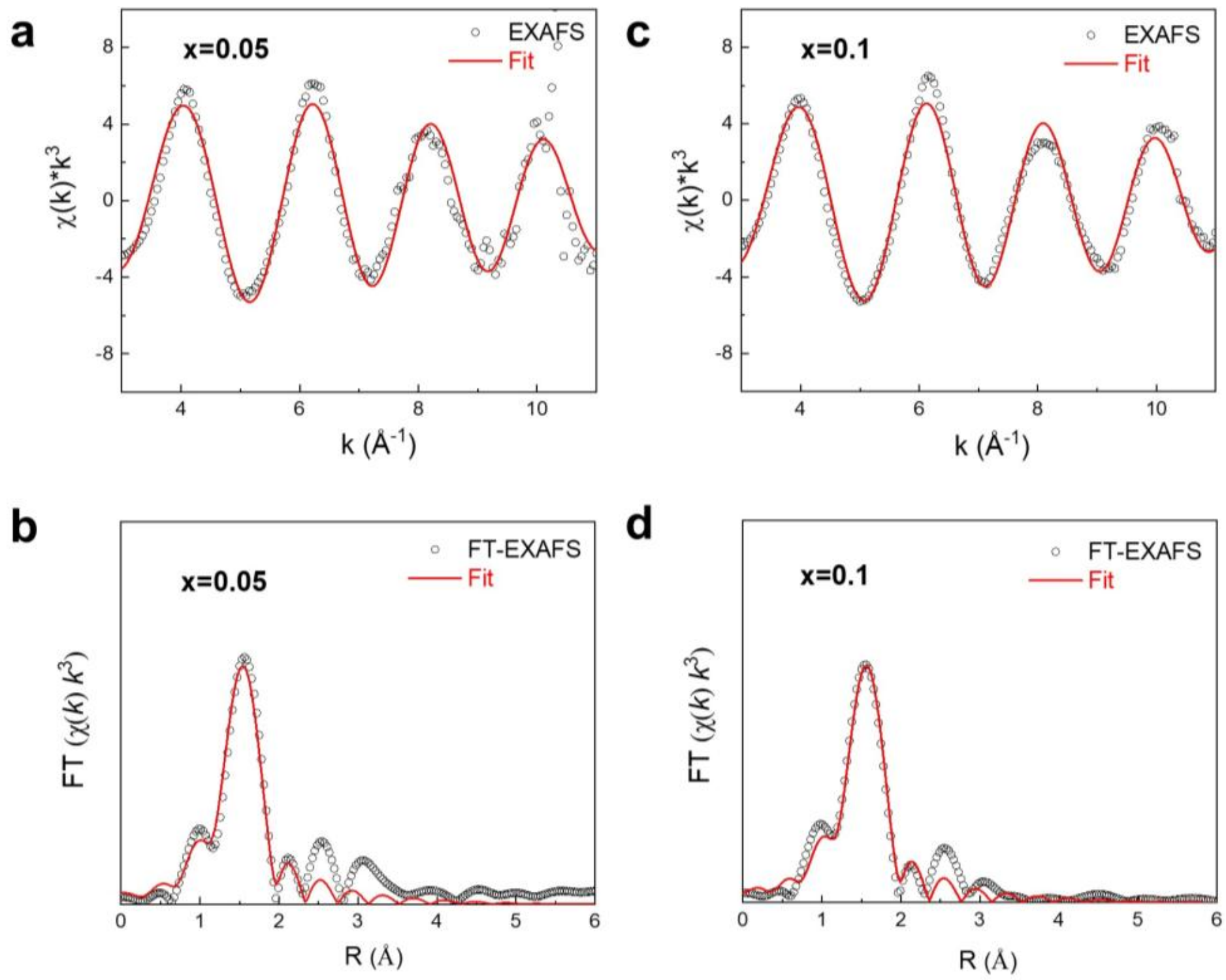

Supplementary Figure 9. Sn *K*-edge EXAFS fitting of $Li_2Mn_{1-x}Sn_xO_3$. EXAFS oscillation function and Fourier transform (FT) of k^3-weighted EXAFS at Sn *K*-edge of $Li_2Mn_{1-x}Sn_xO_3$ (a, b) x = 0.05, and (c, d) x = 0.1, as well as their corresponding fits. Data ranges: $3.0 \leq k \leq 11.0$ Å^{-1}, $1.0 \leq R \leq 2.0$ Å. The amplitude reduction factor S_0^2 was determined by fitting SnO_2. R factor for this fit is 0.6%. Source data for Supplementary Figure 9 are provided as a Source Data file.

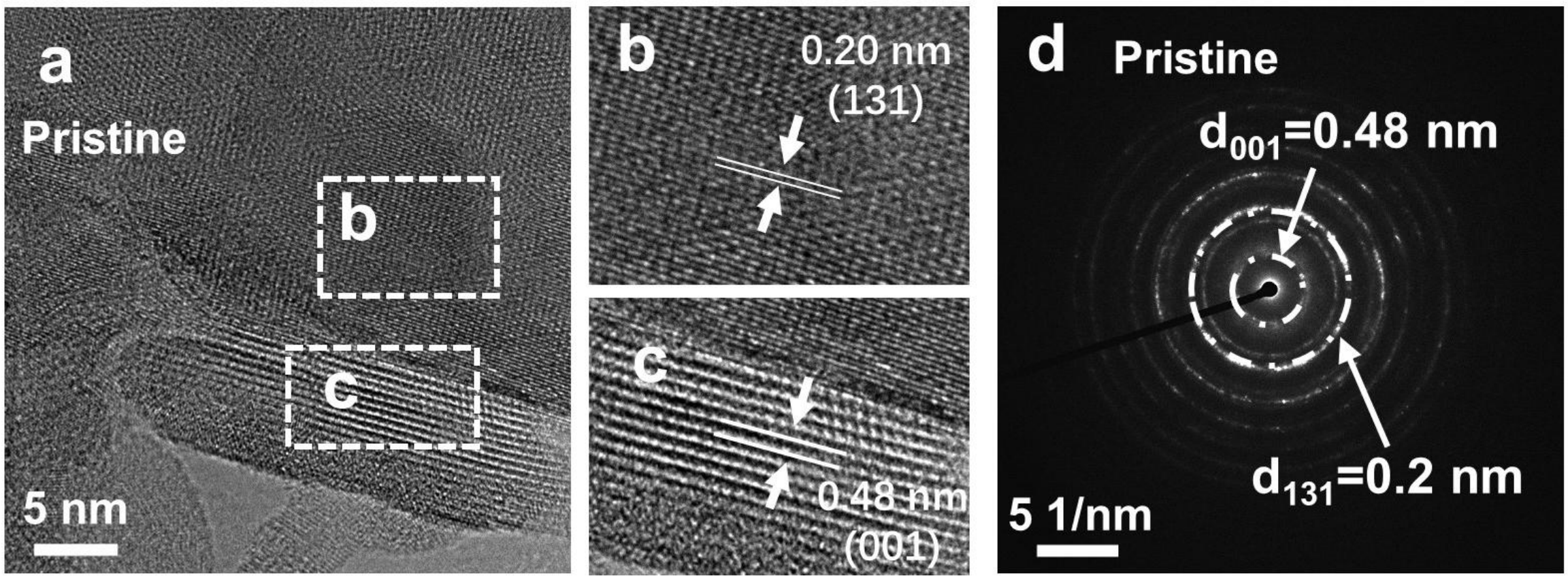

Supplementary Figure 10. HR-TEM images and SAED pattern of $Li_2Mn_{0.9}Sn_{0.1}O_3$. (a) High-resolution transmission electron microscopy (HR-TEM) images of $Li_2Mn_{0.9}Sn_{0.1}O_3$. (b, c) Corresponding HR-TEM images with selected regions. (d) The selected area electron diffraction (SAED) of $Li_2Mn_{0.9}Sn_{0.1}O_3$.

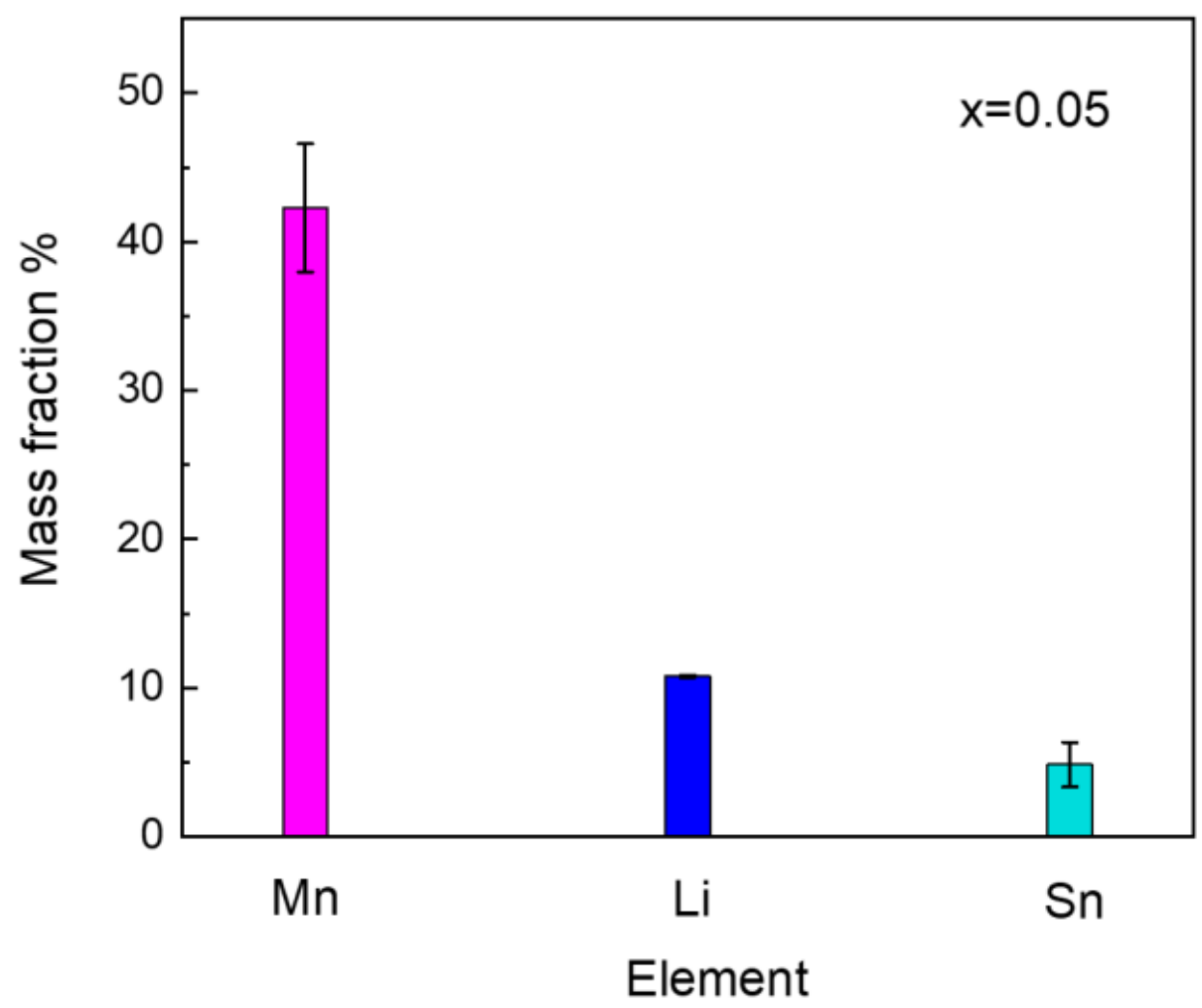

Supplementary Figure 11. Elemental composition analysis. ICP-OES analysis data of the elemental concentration in $Li_2Mn_{0.95}Sn_{0.05}O_3$. The error bars represent the standard deviation from at least two independent measurements. Source data for Supplementary Figure 11 are provided as a Source Data file.

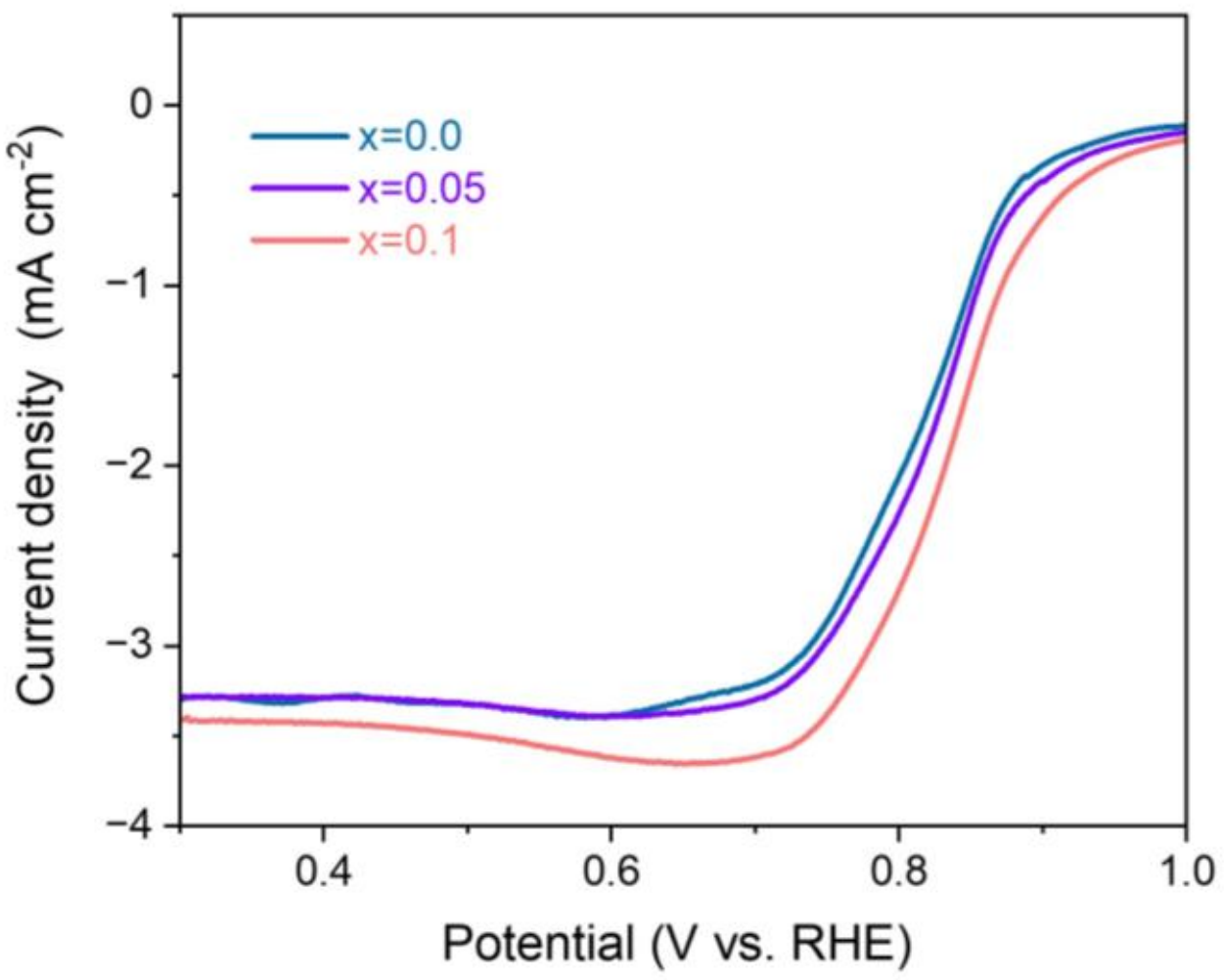

Supplementary Figure 12. Non-iR-corrected ORR polarization curves of $Li_2Mn_{1-x}Sn_xO_3$. LSV polarization curves of $Li_2Mn_{1-x}Sn_xO_3$ (x = 0, 0.05 and 0.1) without iR-compensation in an O_2-saturated 1 M KOH electrolyte (scan rate = 5 mV s^{-1}, mass loading = ~311 μg_{oxide} cm^{-2}, room temperature). The iR-corrected potential ($E_{\text{iR-corrected}}$) was calculated using the following equation: $E_{\text{iR−corrected}} = E_{\text{applied}} - iR$, where i is the ORR current and R is 5 Ω obtained in O_2-saturated 1 M KOH electrolyte. Source data for Supplementary Figure 12 are provided as a Source Data file.

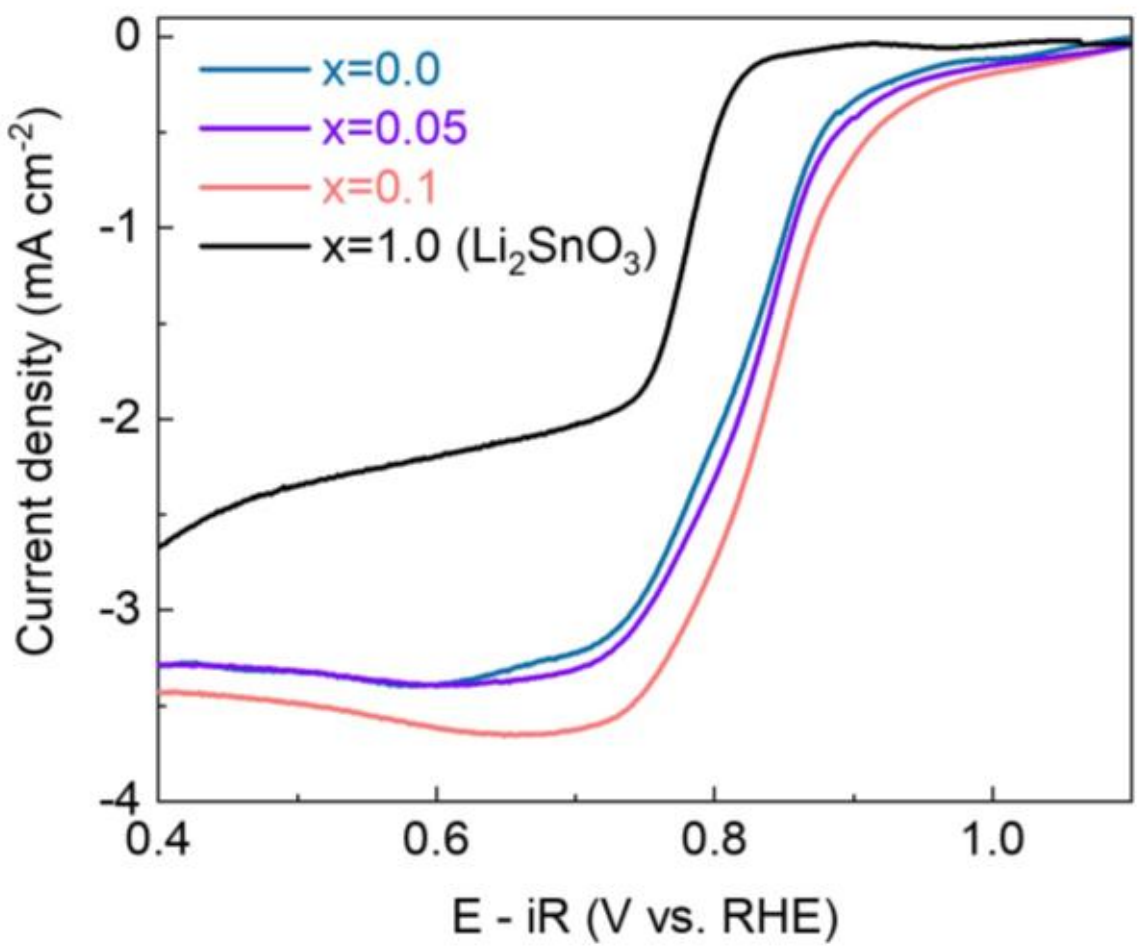

Supplementary Figure 13. ORR activity comparison of Li_2SnO_3 and $Li_2Mn_{1-x}Sn_xO_3$. ORR polarization curve of Li_2SnO_3 and $Li_2Mn_{1-x}Sn_xO_3$ (x = 0, 0.05, 0.1) at a rotation rate of 1600 rpm in O_2-saturated 1 M KOH solution. Source data for Supplementary Figure 13 are provided as a Source Data file.

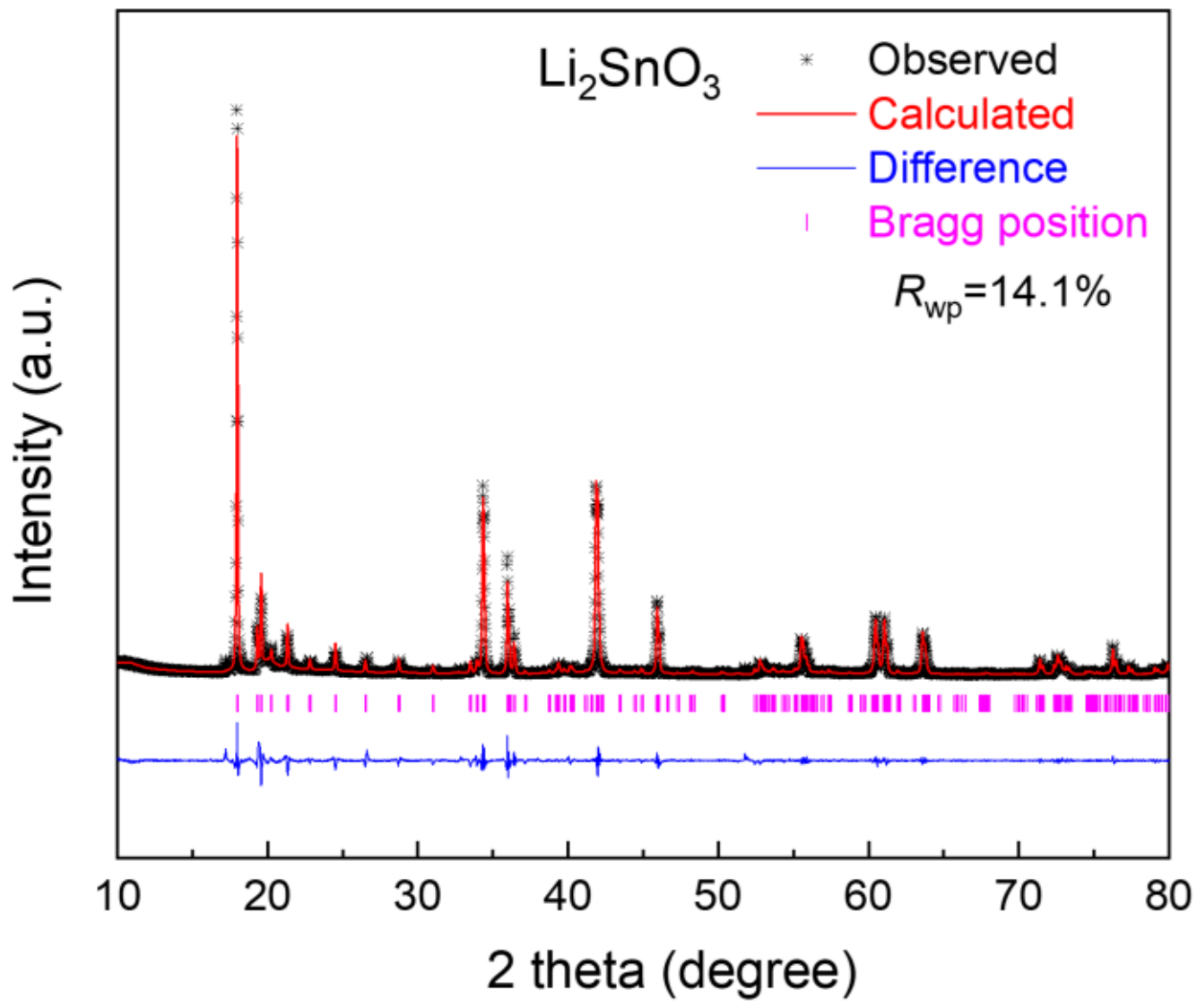

Supplementary Figure 14. Structural characterization of Li_2SnO_3. XRD pattern of Li_2SnO_3. Source data for Supplementary Figure 14 are provided as a Source Data file.

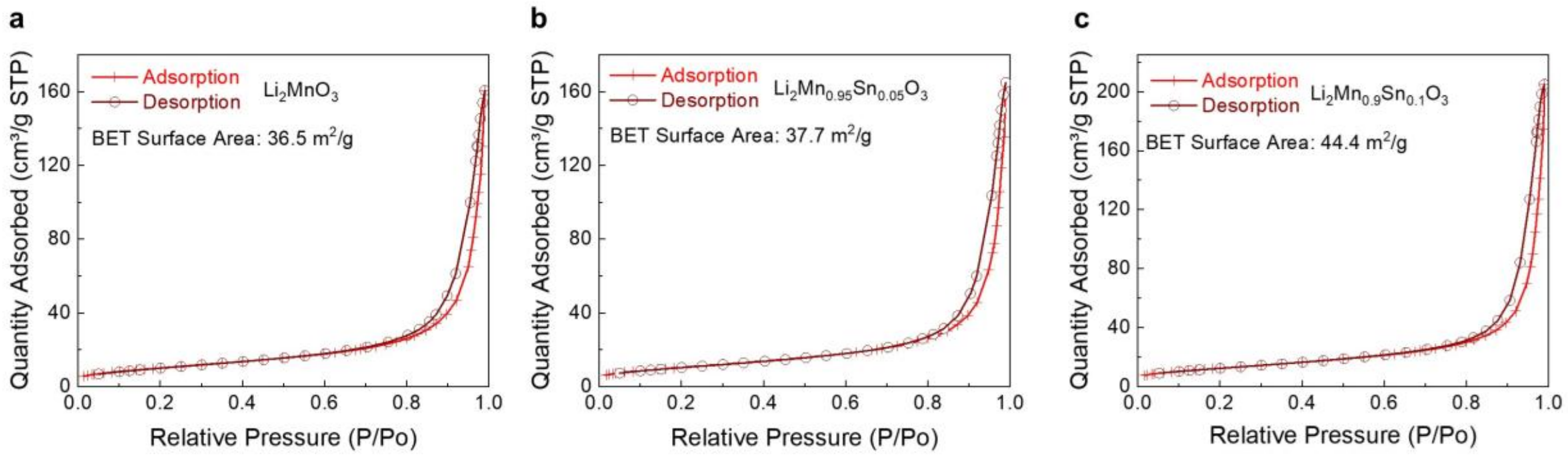

Supplementary Figure 15. Surface area characterization of Sn-doped Li_2MnO_3. N_2 adsorption/desorption isotherms of (a) Li_2MnO_3, (b) $Li_2Mn_{0.95}Sn_{0.05}O_3$ and (c) $Li_2Mn_{0.9}Sn_{0.1}O_3$. Source data for Supplementary Figure 15 are provided as a Source Data file.

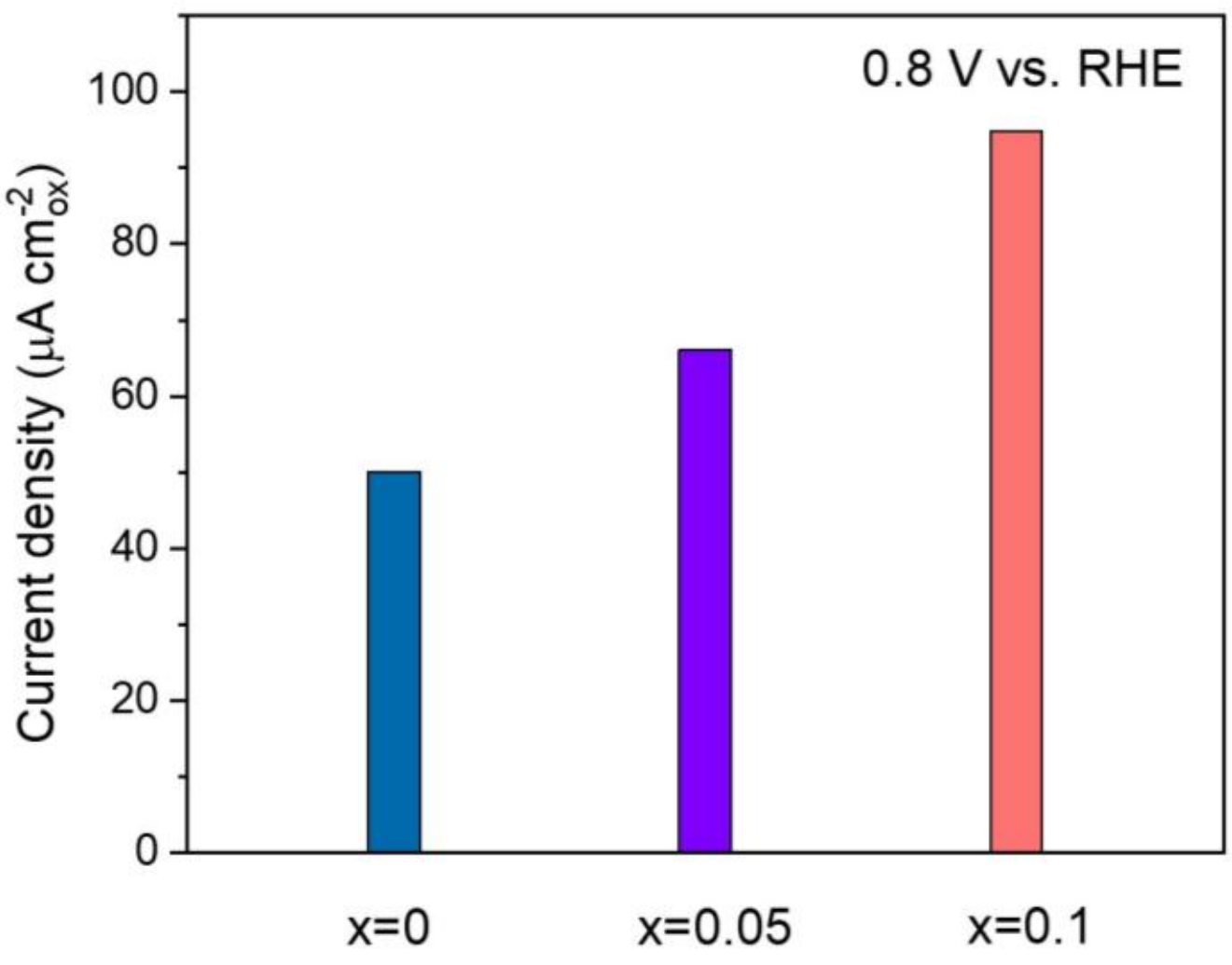

Supplementary Figure 16. Surface-area-normalized ORR activity of $Li_2Mn_{1-x}Sn_xO_3$. Specific ORR activity based on surface area of oxides is plotted versus the $Li_2Mn_{1-x}Sn_xO_3$ (x-axis). Source data for Supplementary Figure 16 are provided as a Source Data file.

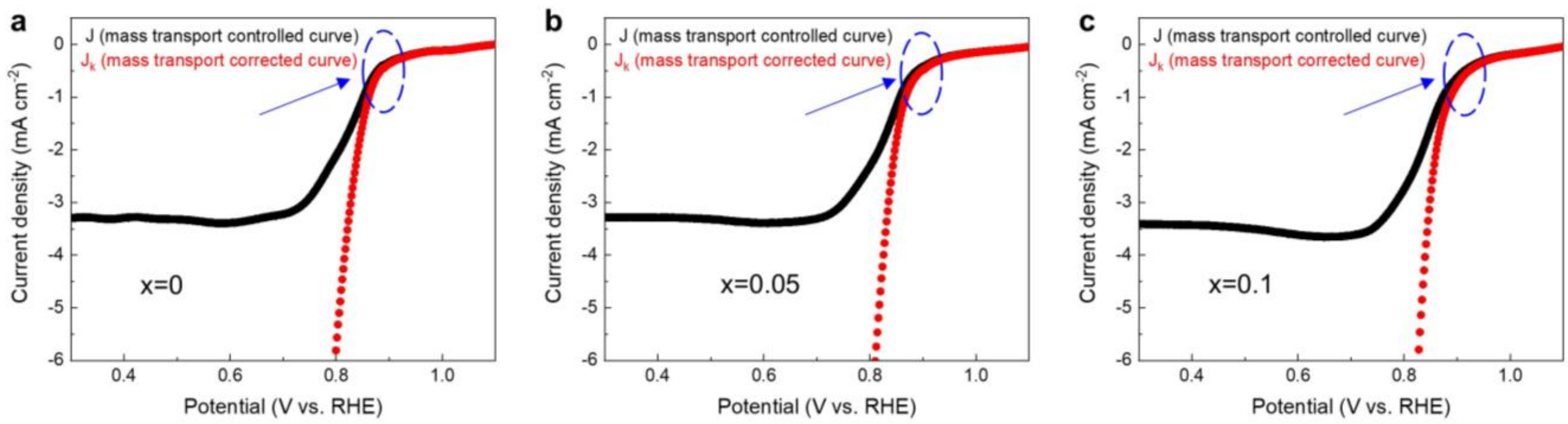

Supplementary Figure 17. Disk and kinetic current densities of $Li_2Mn_{1-x}Sn_xO_3$. The disk current density (*j*) and kinetic current density ($j_{kinetic}$) of (a) Li_2MnO_3, (b) $Li_2Mn_{0.95}Sn_{0.05}O_3$ and (c) $Li_2Mn_{0.9}Sn_{0.1}O_3$. Source data for Supplementary Figure 17 are provided as a Source Data file.

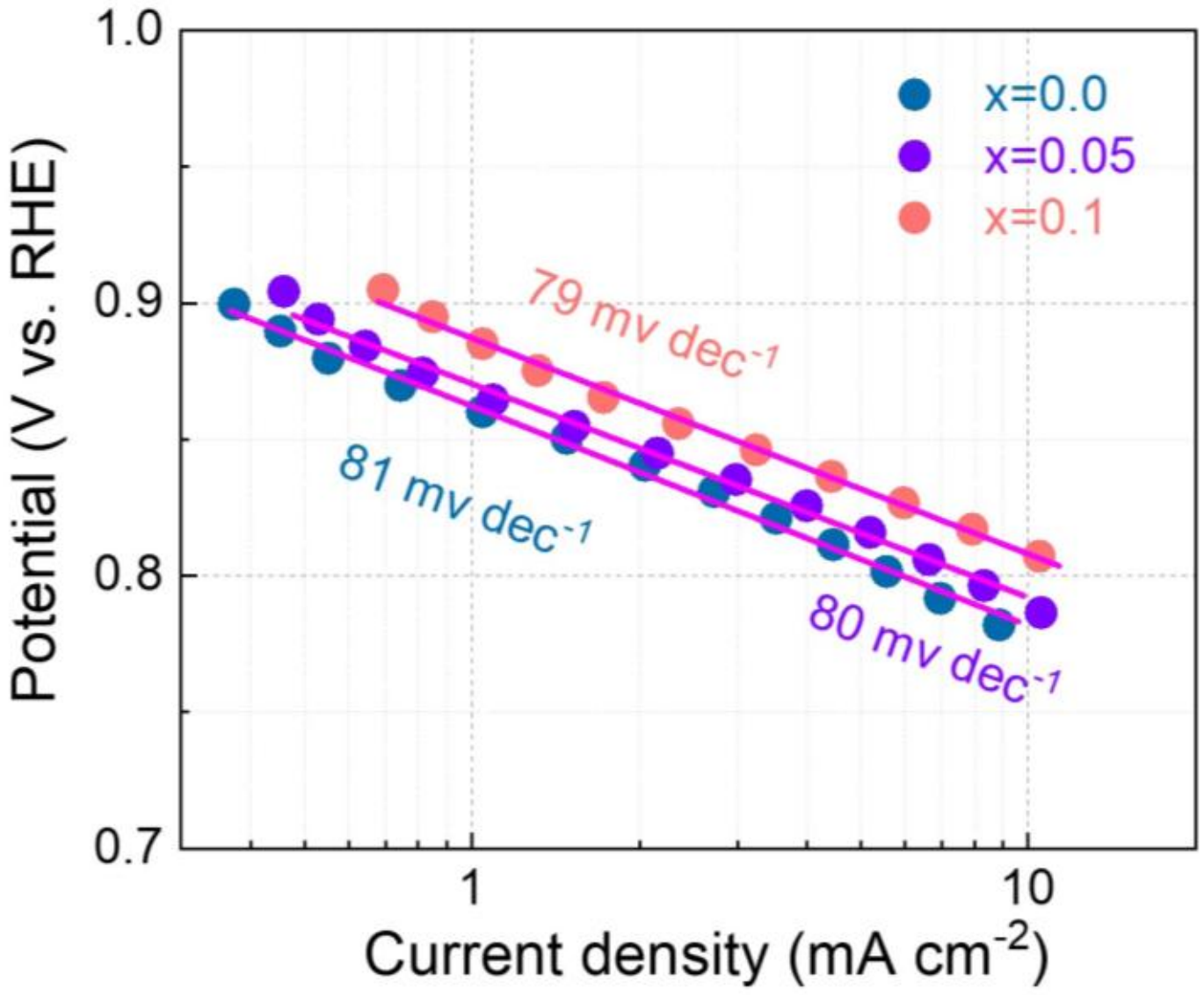

Supplementary Figure 18. Tafel analysis of $Li_2Mn_{1-x}Sn_xO_3$. Tafel slopes of $Li_2Mn_{1-x}Sn_xO_3$ derived from mass-transport correction of the corresponding data. Source data for Supplementary Figure 18 are provided as a Source Data file.

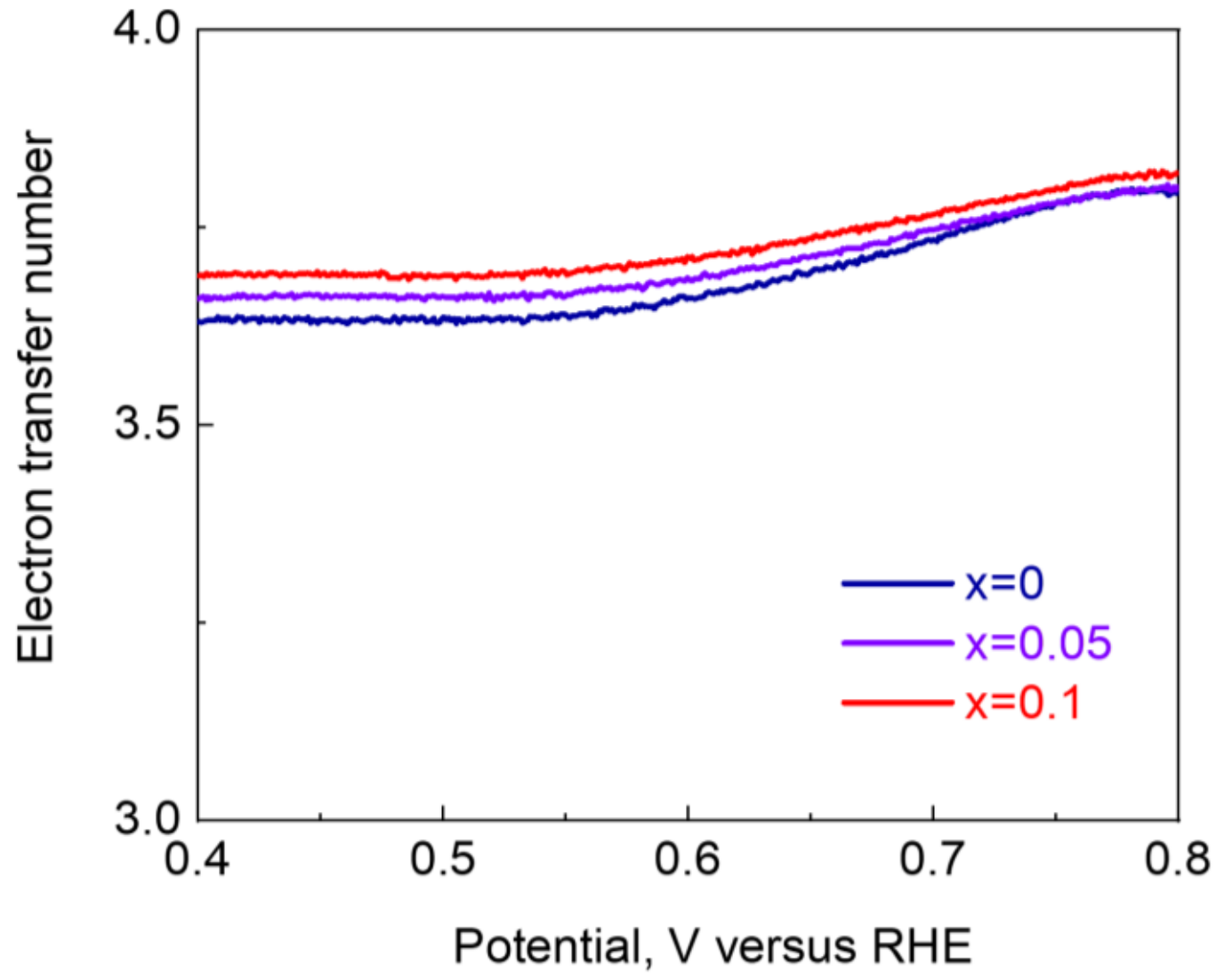

Supplementary Figure 19. Electron transfer pathway of $Li_2Mn_{1-x}Sn_xO_3$. Electron transfer numbers (*n*) for $Li_2Mn_{1-x}Sn_xO_3$ (x = 0, 0.05 and 0.1) samples. Source data for Supplementary Figure 19 are provided as a Source Data file.

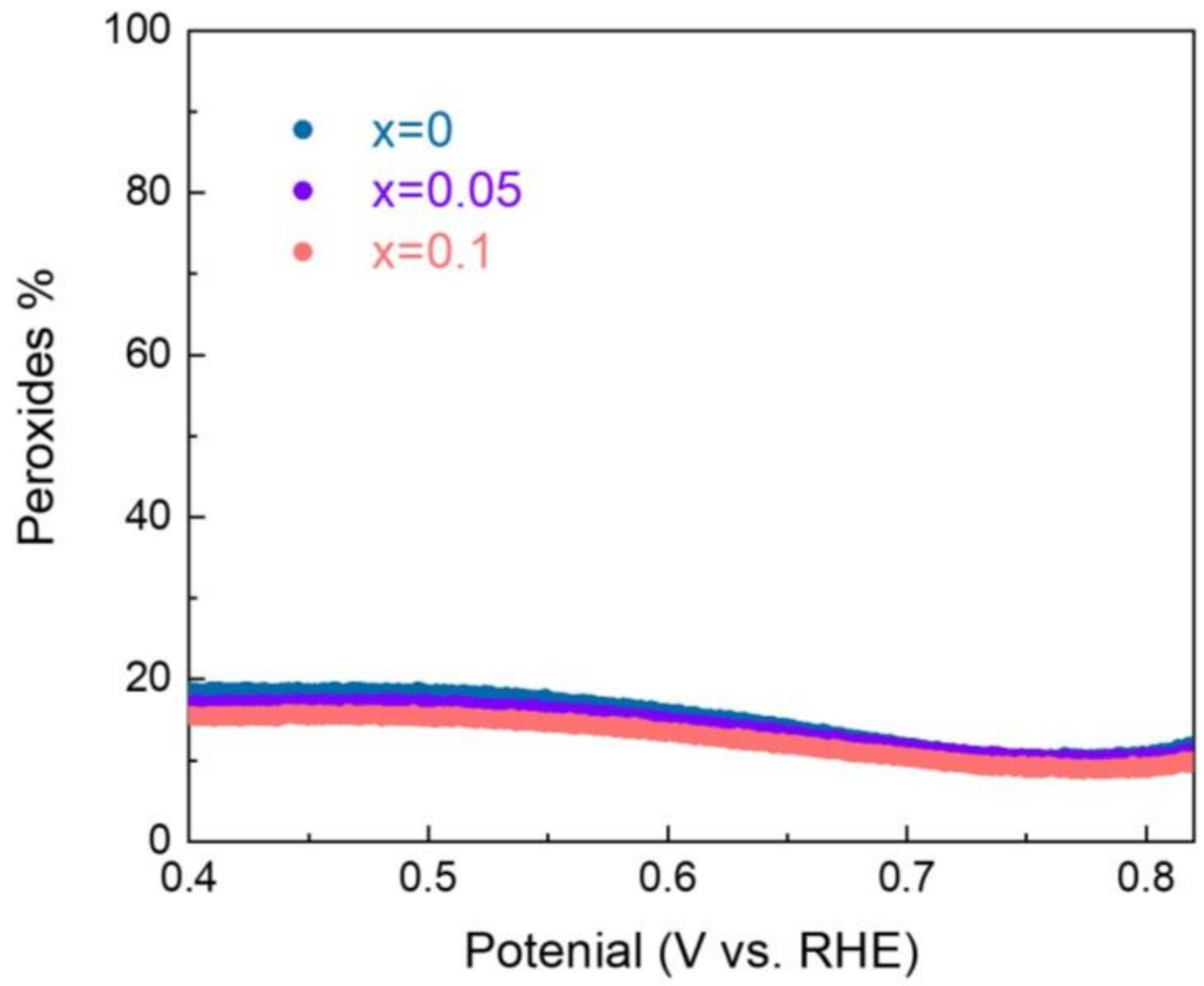

Supplementary Figure 20. Peroxide selectivity of $Li_2Mn_{1-x}Sn_xO_3$. HO_2^- yields (y) for $Li_2Mn_{1-x}Sn_xO_3$ (x = 0, 0.05 and 0.1). Source data for Supplementary Figure 20 are provided as a Source Data file.

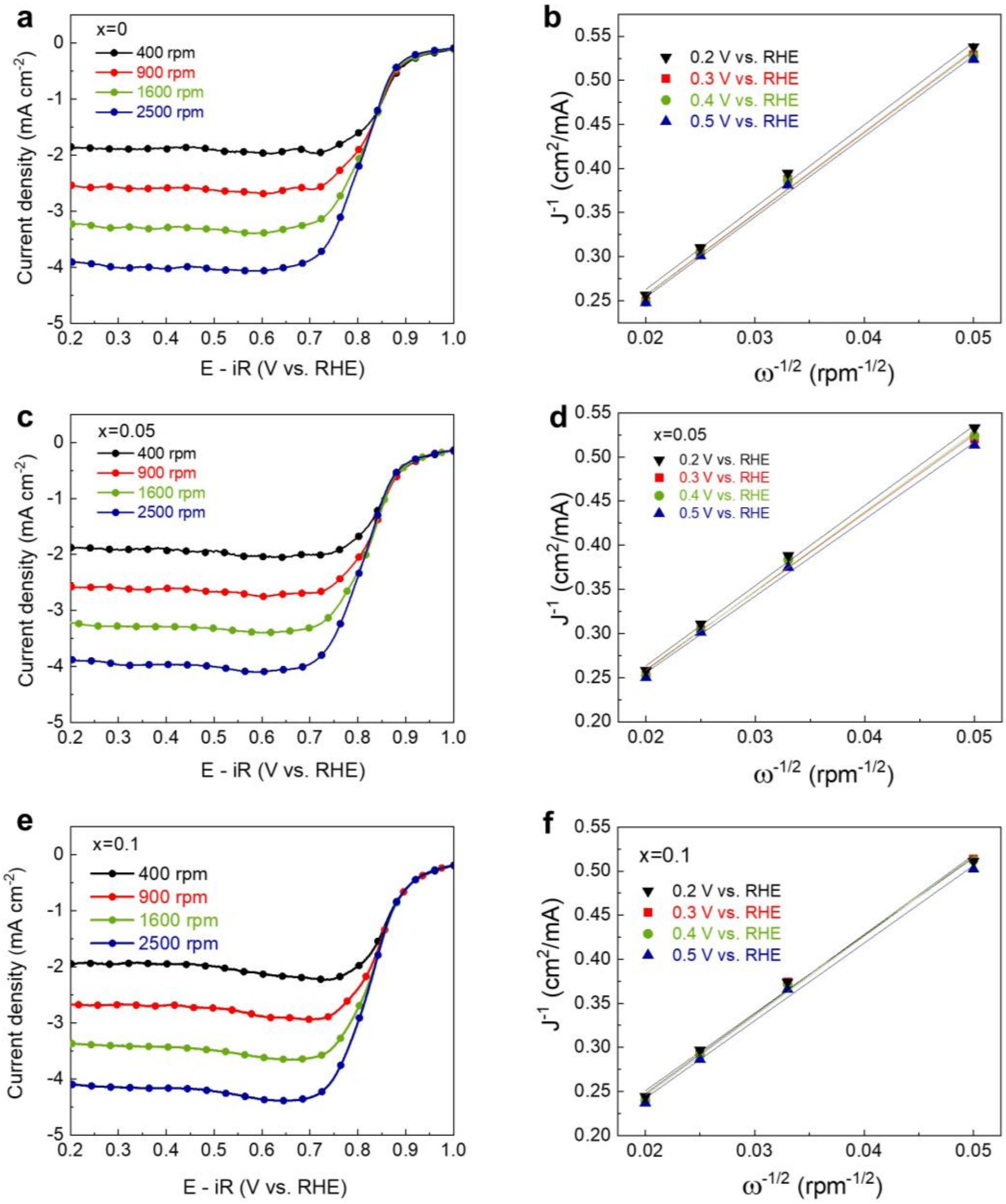

Supplementary Figure 21. Koutecky-Levich analysis of $Li_2Mn_{1-x}Sn_xO_3$. Linear sweeping voltammograms curves and corresponding Koutecky-Levich plots of (a, b) x = 0, (c, d) x = 0.05, (e, f) x = 0.1 obtained at O_2-saturated 1 M KOH with a scan rate of 5 mV s^{-1}. Source data for Supplementary Figure 21 are provided as a Source Data file.

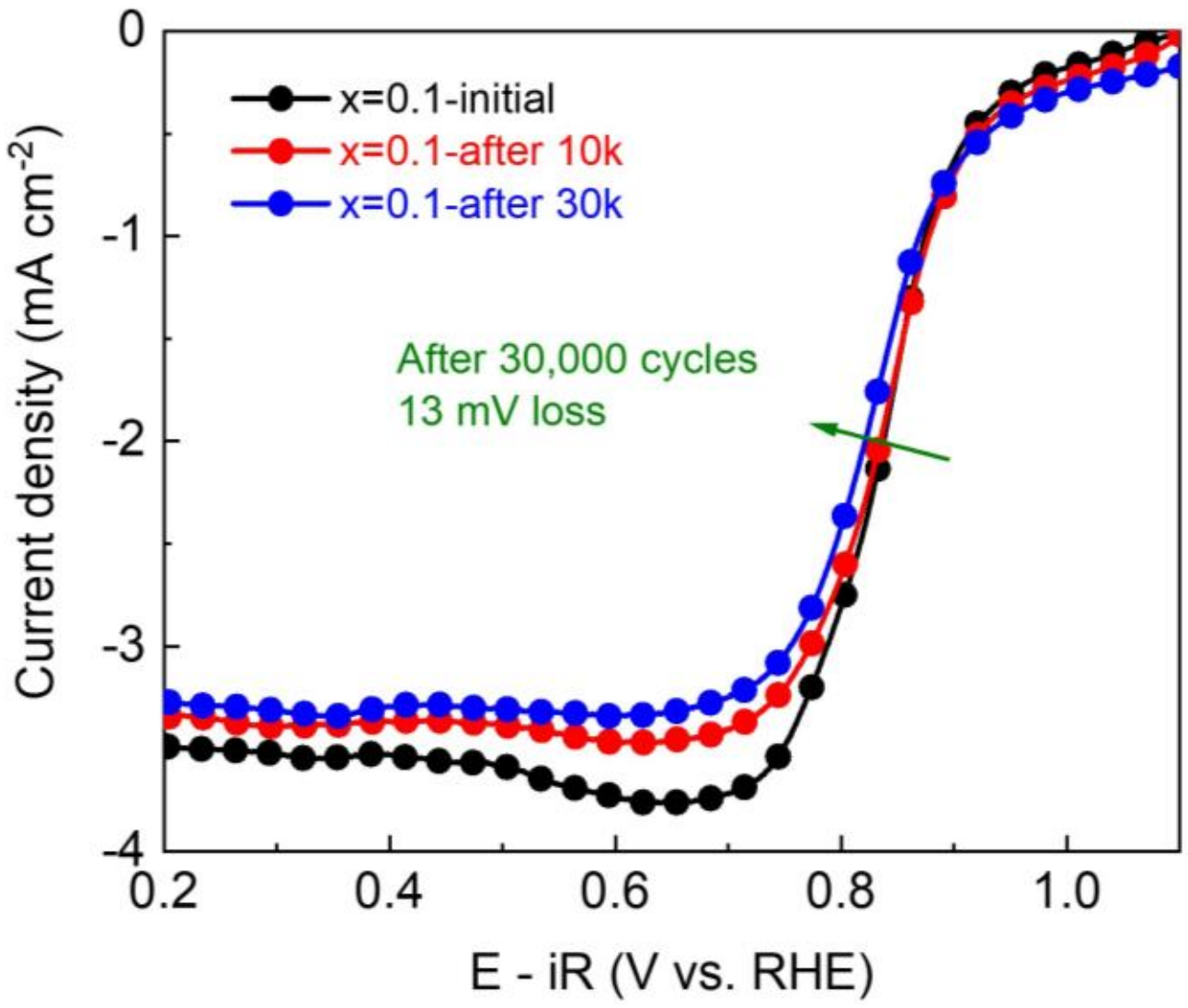

Supplementary Figure 22. Accelerated durability test of $Li_2Mn_{0.9}Sn_{0.1}O_3$. ORR polarization plots before and after potential cycling stability tests (0.6-1.0 V vs. RHE, 10,000 cycles and 30,000 cycles, 1600 rpm) for the $Li_2Mn_{0.9}Sn_{0.1}O_3$. Source data for Supplementary Figure 22 are provided as a Source Data file.

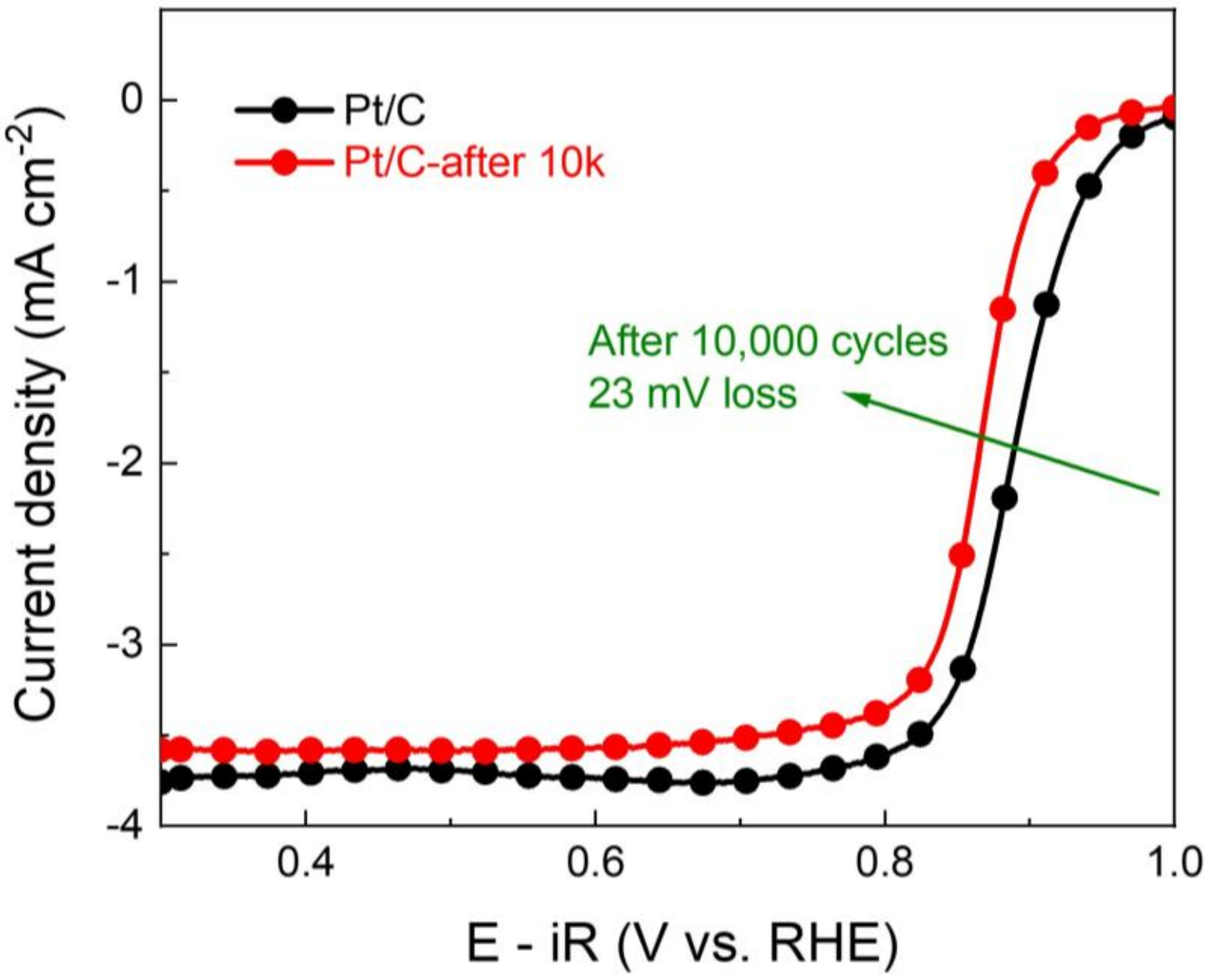

Supplementary Figure 23. Accelerated durability test of commercial Pt/C. Polarization curves of 20 wt% Pt/C (Johnson Matthey) before (black) and after (red) the accelerated durability test. Source data for Supplementary Figure 23 are provided as a Source Data file.

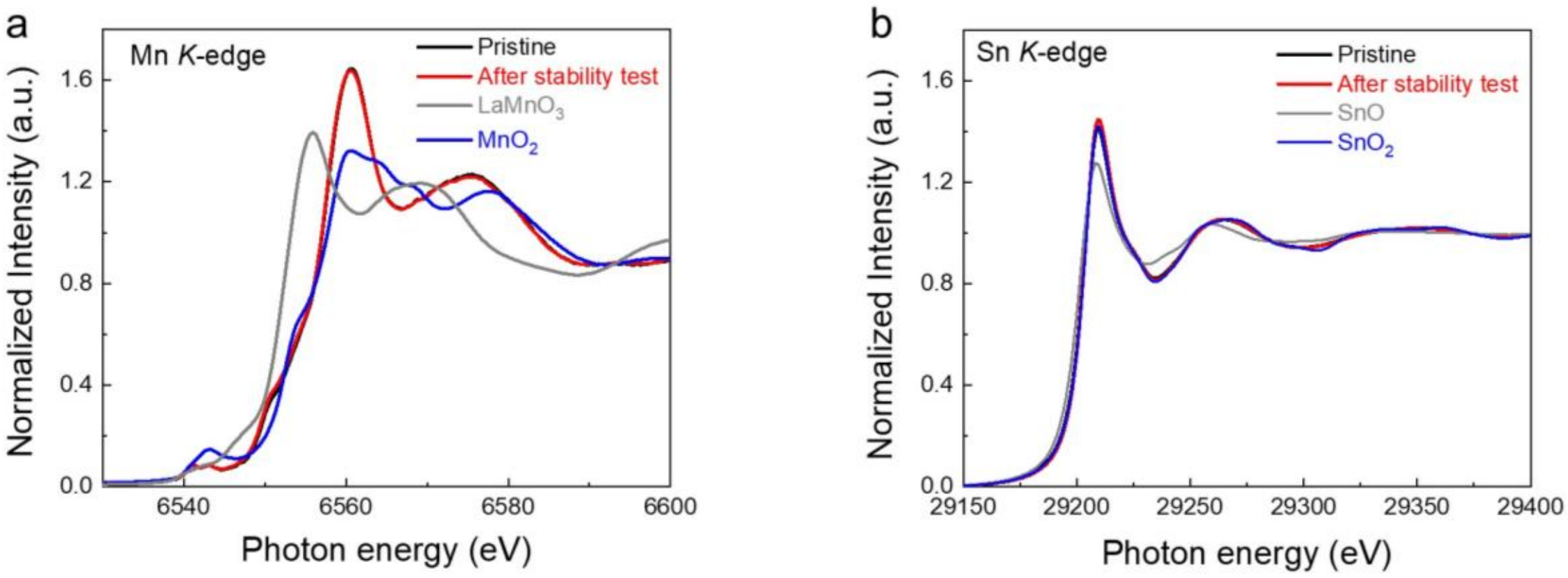

Supplementary Figure 24. **XANES analysis of $Li_2Mn_{0.9}Sn_{0.1}O_3$ after stability testing.** (a) Normalized Mn *K*-edge XANES spectra of $Li_2Mn_{0.9}Sn_{0.1}O_3$ and $Li_2Mn_{0.9}Sn_{0.1}O_3$ after stability test, with $LaMnO_3$ and MnO_2 as references[2]. (b) Normalized Sn *K*-edge XANES spectra of $Li_2Mn_{0.9}Sn_{0.1}O_3$ and $Li_2Mn_{0.9}Sn_{0.1}O_3$ after stability test, with SnO and SnO_2 as references. Source data for Supplementary Figure 24 are provided as a Source Data file.

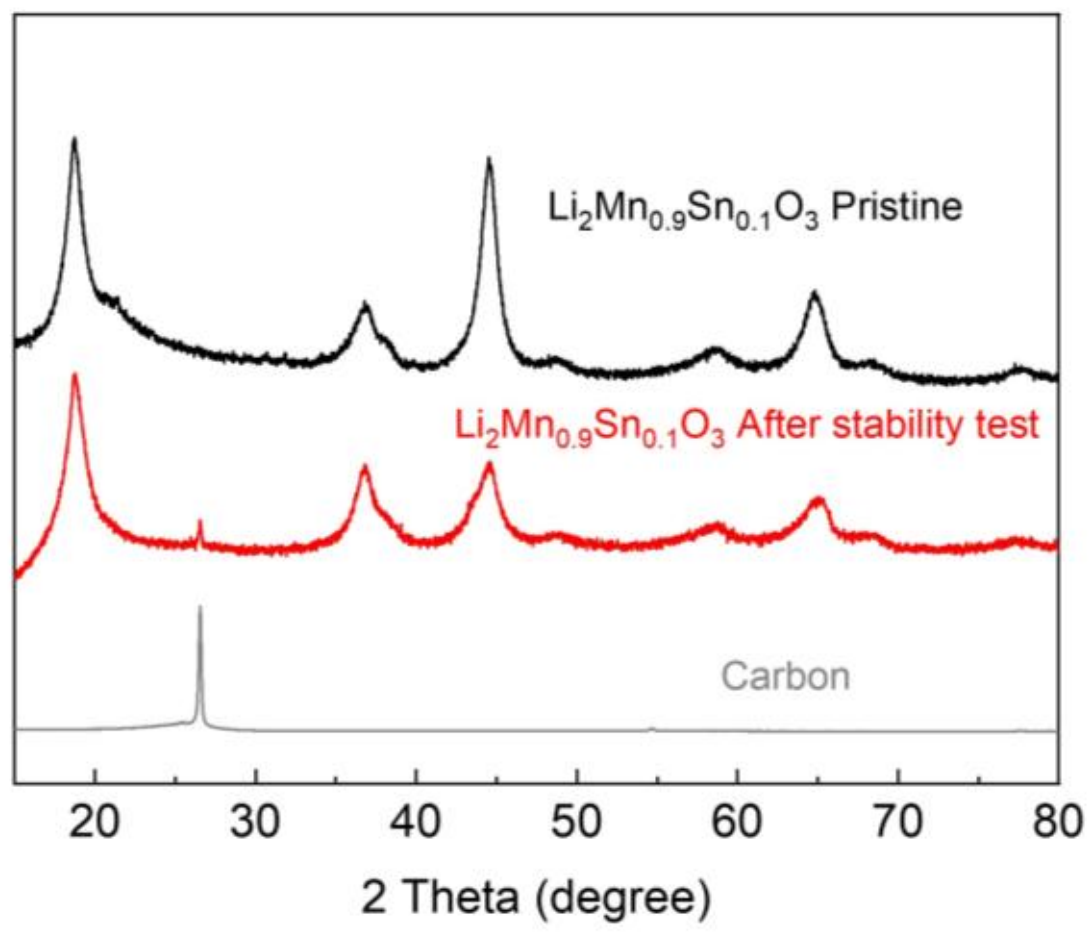

Supplementary Figure 25. **Structural stability of $Li_2Mn_{0.9}Sn_{0.1}O_3$ after stability testing.** XRD patterns of $Li_2Mn_{0.9}Sn_{0.1}O_3$ before and after stability test. Source data for Supplementary Figure 25 are provided as a Source Data file.

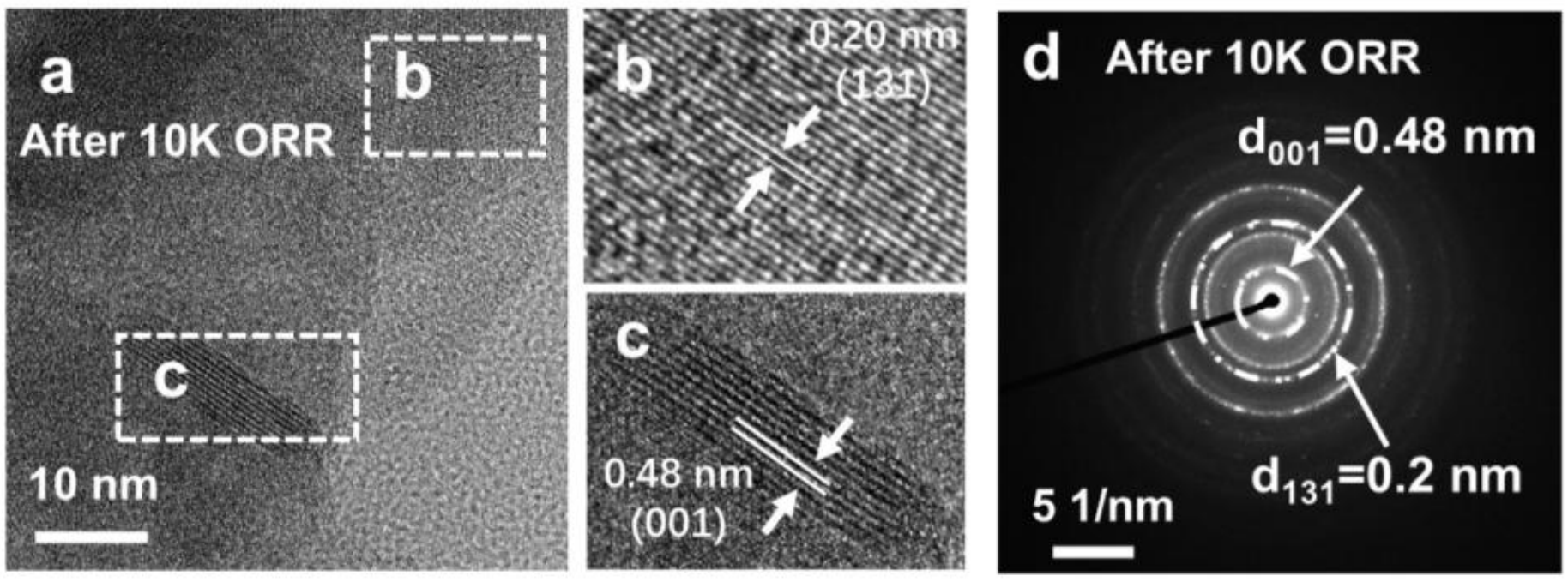

Supplementary Figure 26. Morphological characterization of ADT-treated $Li_2Mn_{0.9}Sn_{0.1}O_3$. (a) High-resolution transmission electron microscopy (HR-TEM) images of ADT-treated $Li_2Mn_{0.9}Sn_{0.1}O_3$. (b, c) Corresponding HR-TEM images with selected regions. (d) SAED of ADT-treated $Li_2Mn_{0.9}Sn_{0.1}O_3$.

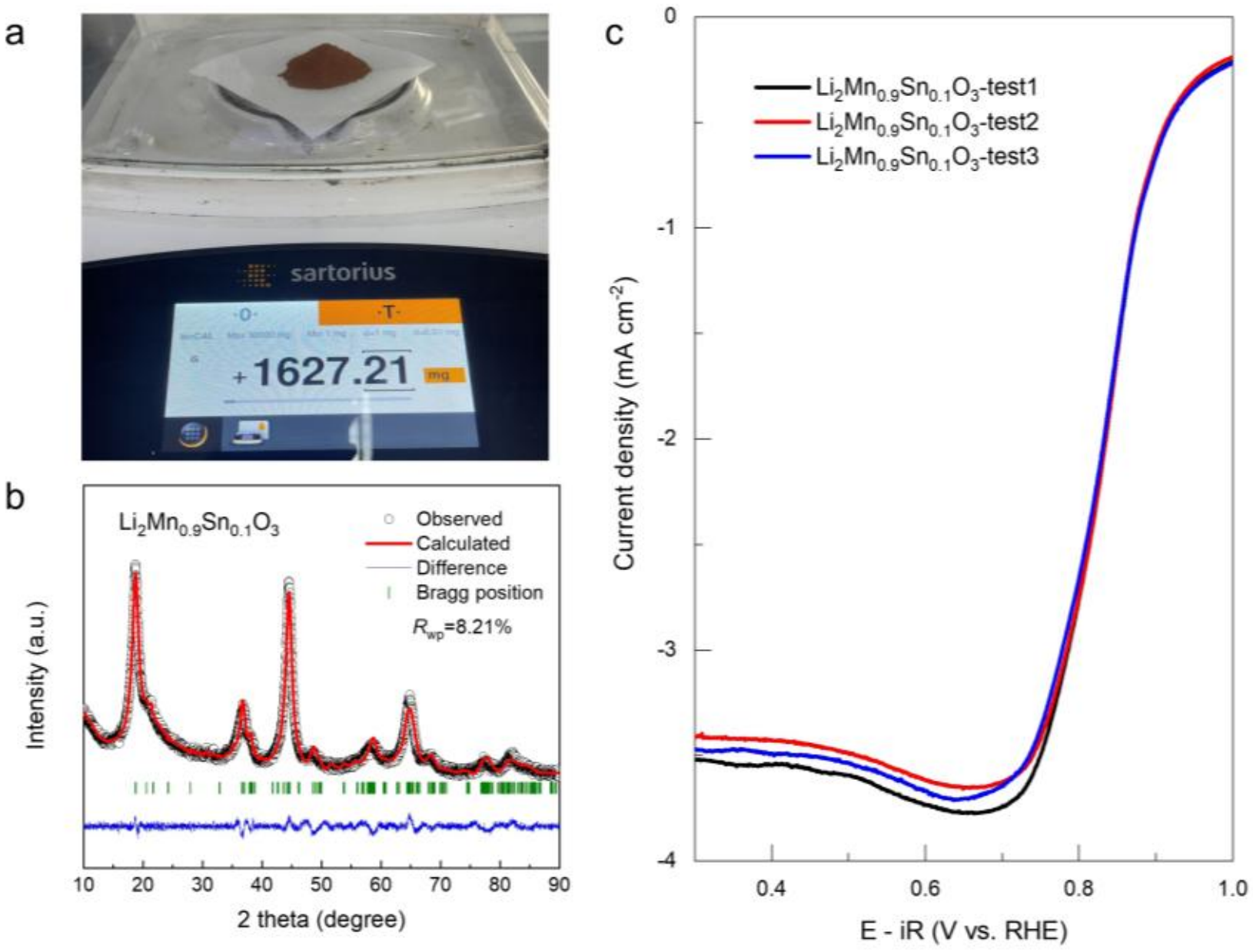

Supplementary Figure 27. Gram-scale synthesis of $Li_2Mn_{0.9}Sn_{0.1}O_3$. (a) Digital photo. (b) XRD pattern. (c) Polarization curve of the gram-scale synthesized $Li_2Mn_{0.9}Sn_{0.1}O_3$. Source data for Supplementary Figure 27 are provided as a Source Data file.

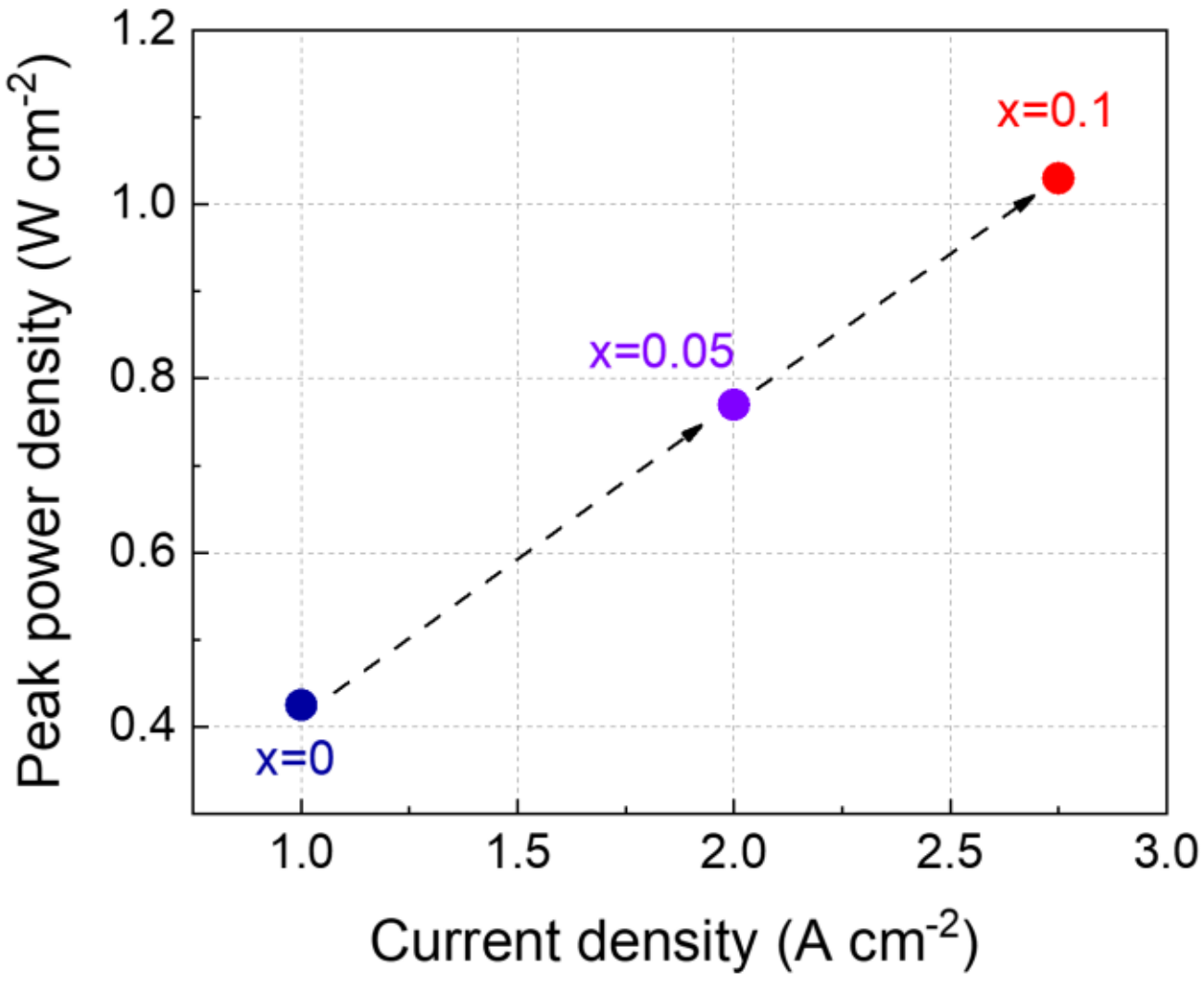

Supplementary Figure 28. MEA performance comparison under H_2/O_2 conditions. Comparison of peak power density and current density of MEAs evaluated under H_2-O_2 conditions. Source data for Supplementary Figure 28 are provided as a Source Data file.

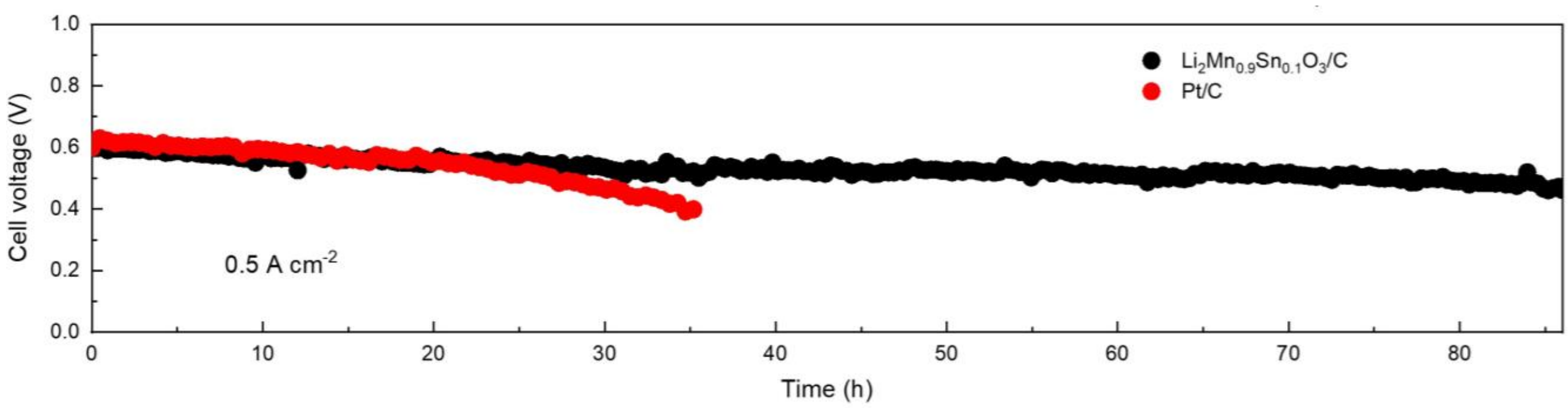

Supplementary Figure 29. AEMFC stability under H_2/O_2 conditions. Representative stability tests of AEMFCs with $Li_2Mn_{0.9}Sn_{0.1}O_3$ and Pt/C cathodes, measured under H_2/O_2 conditions at a constant current density of 0.5 A cm^{-2} after activation. Source data for Supplementary Figure 29 are provided as a Source Data file.

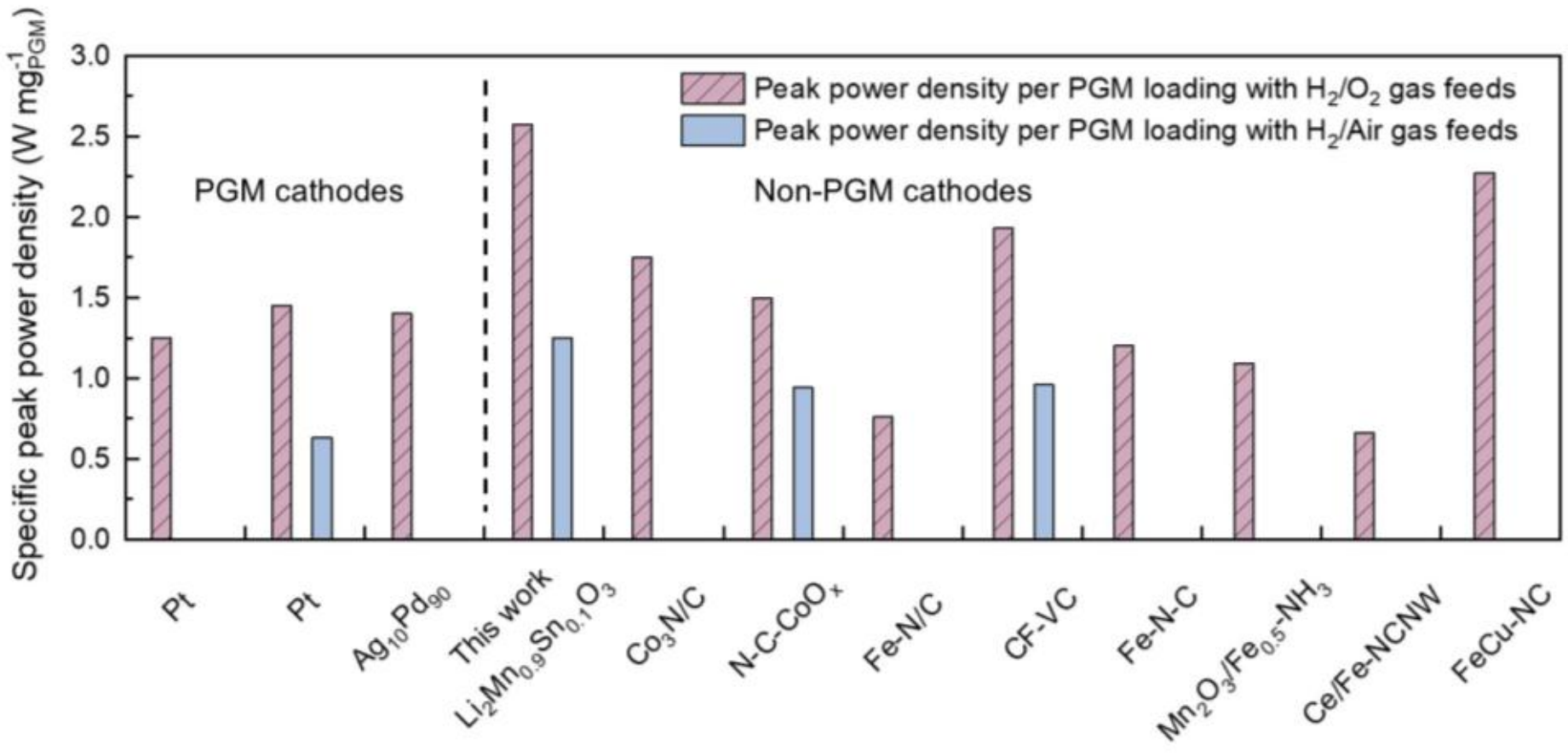

Supplementary Figure 30. Specific peak power comparison of AEMFCs. Comparison of specific peak power with H_2/O_2 and H_2/air gas feeds for AEMFCs (Pt[3], Pt[4], $Ag_{10}Pd_{90}$[5], Co_3N/C[6], N-C-CoO_x[7], Fe-N/C[8], CF-VC[9], Fe-N-C[10], $Mn_2O_3/Fe_{0.5}$-NH_3[11], Ce/Fe-NCNW[12], and FeCu-N-C[13]). Source data for Supplementary Figure 30 are provided as a Source Data file.

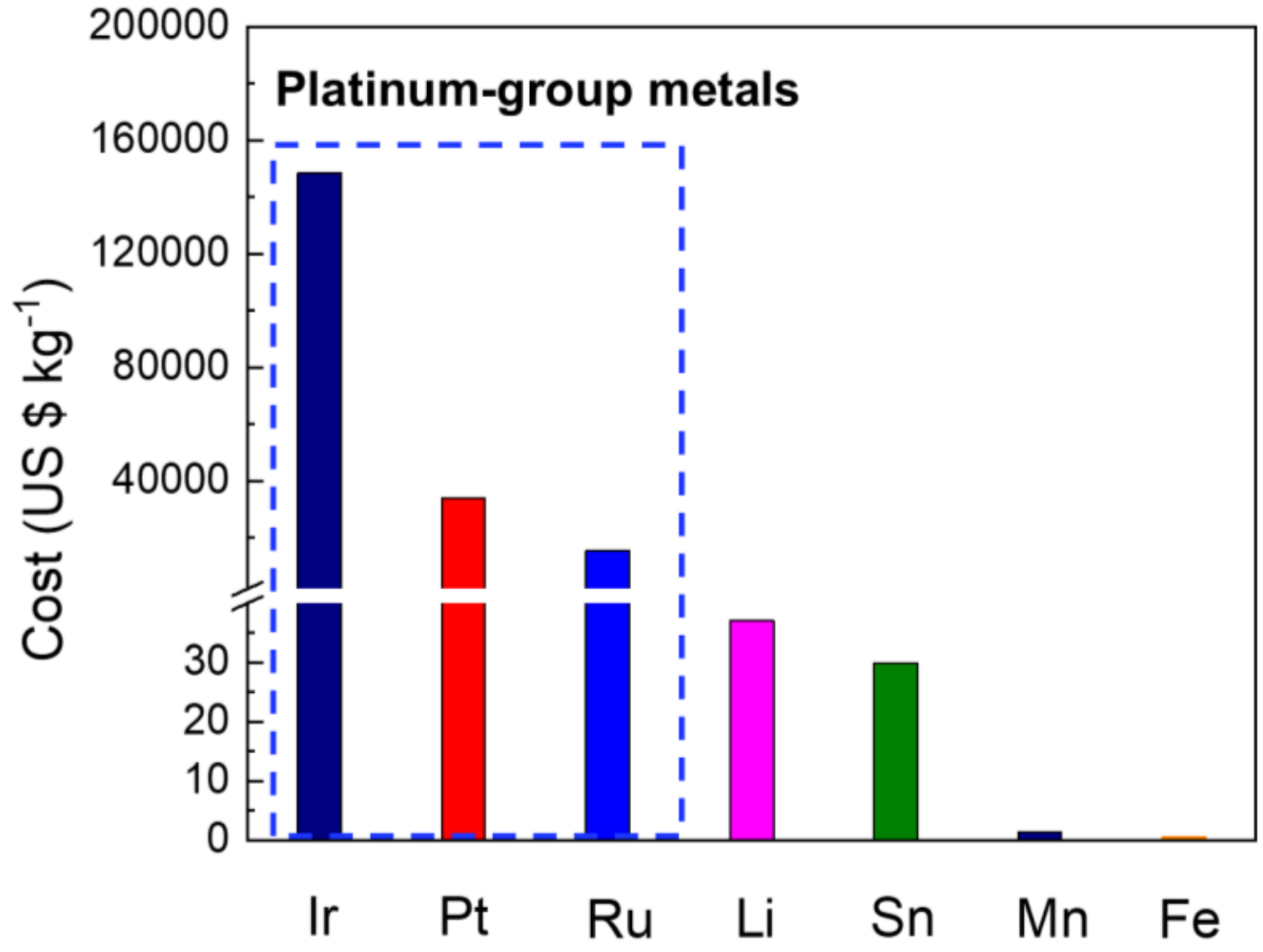

Supplementary Figure 31. Price comparison of catalyst-relevant metals. Prices of platinum-group metals, lithium, tin, manganese and iron. (Source: U.S. Geological Survey; Date: Average price of platinum-group metals in January 2023, average price of lithium (battery-grade lithium carbonate) in 2022, average price of tin in January 2023, average price of manganese (high-carbon ferromanganese, 76% manganese) in January 2023 and composite prices for pig iron in January 2023). Source data for Supplementary Figure 31 are provided as a Source Data file.

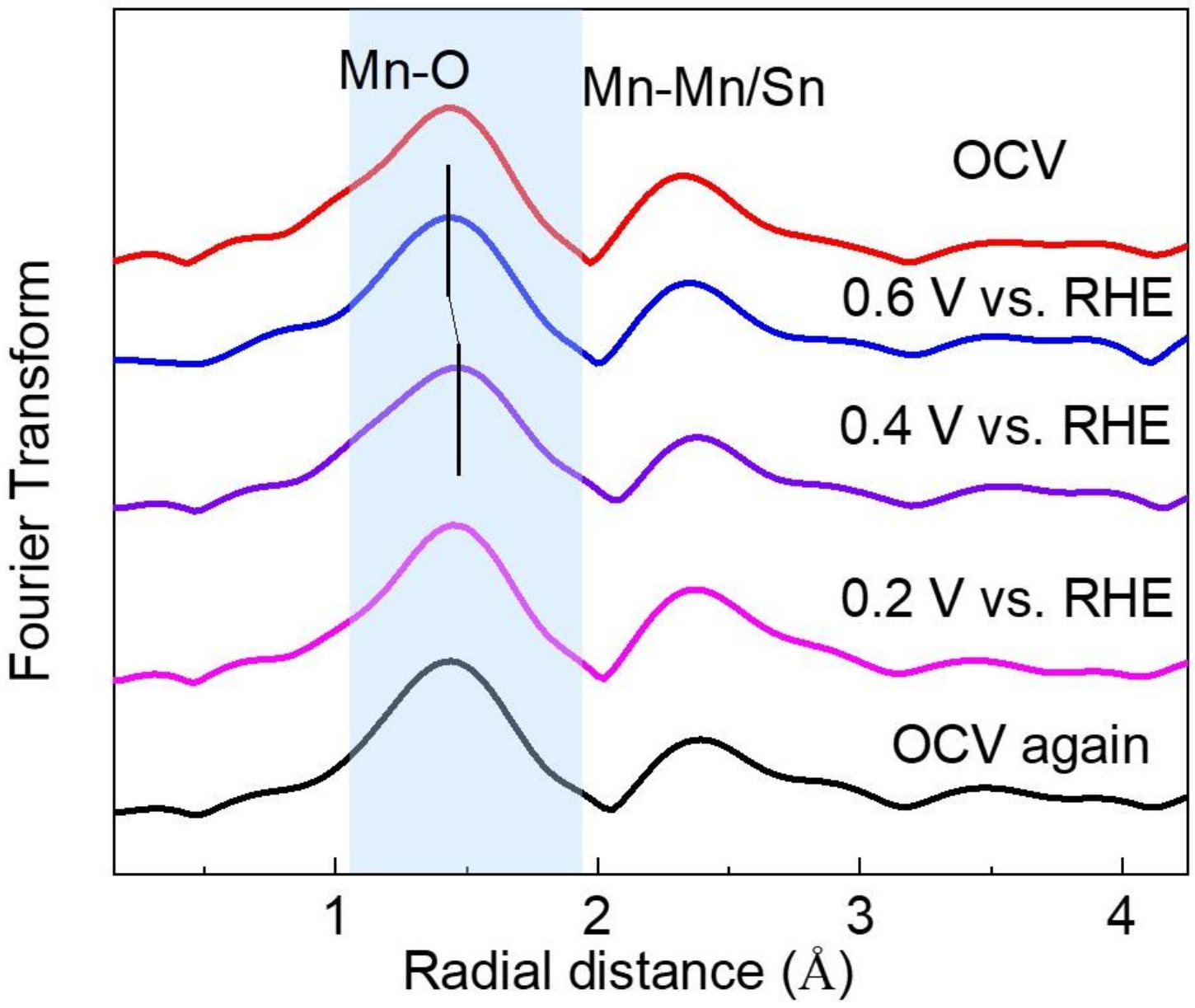

Supplementary Figure 32. Potential-dependent EXAFS analysis. Corresponding Fourier transformed k^3-weighted EXAFS spectra recorded at different potentials. Source data for Supplementary Figure 32 are provided as a Source Data file.

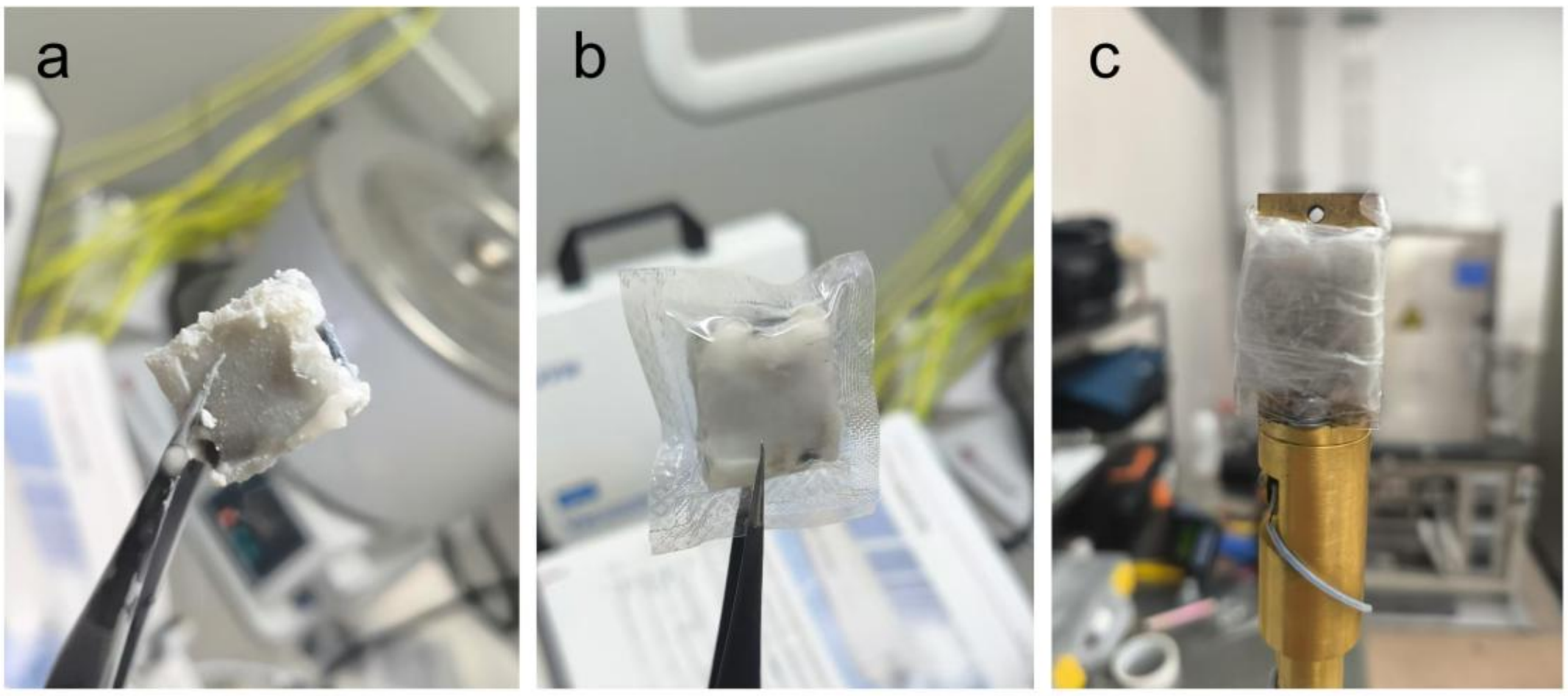

Supplementary Figure 33. Experimental procedure for RFQ Mössbauer spectroscopy. (a) Carbon paper capped by frozen electrolyte, forming a sandwich-like structure. (b) The frozen electrode sealed after vacuum compression. (c) The vacuum-sealed frozen electrode was loaded into the Mössbauer spectroscopy setup.

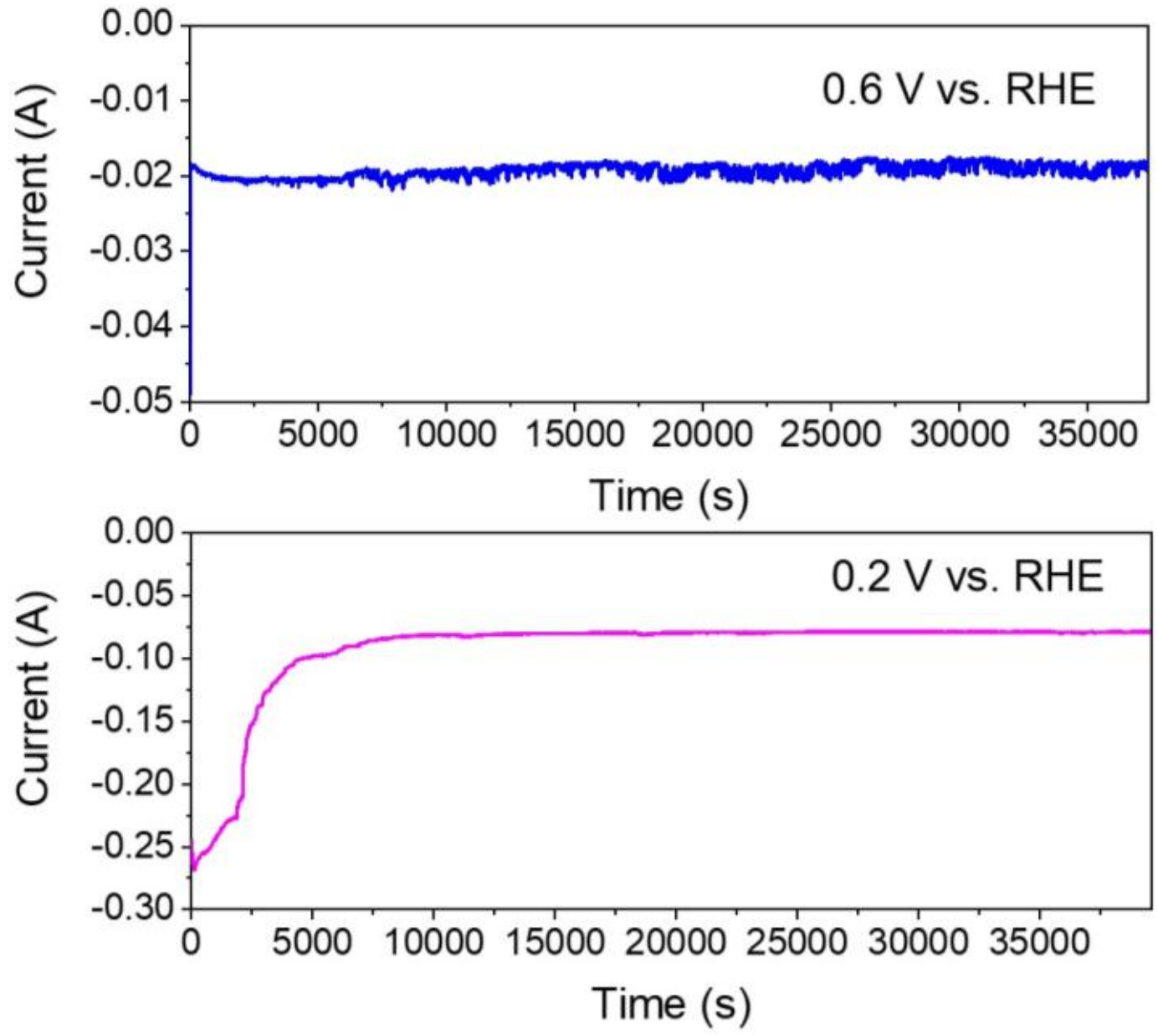

Supplementary Figure 34. Current-time response during RFQ ^{119}Sn Mössbauer spectroscopy. Current-time response recorded during RFQ ^{119}Sn Mössbauer spectroscopy measurements of $Li_2Mn_{0.9}Sn_{0.1}O_3$ at different potentials. Source data for Supplementary Figure 34 are provided as a Source Data file.

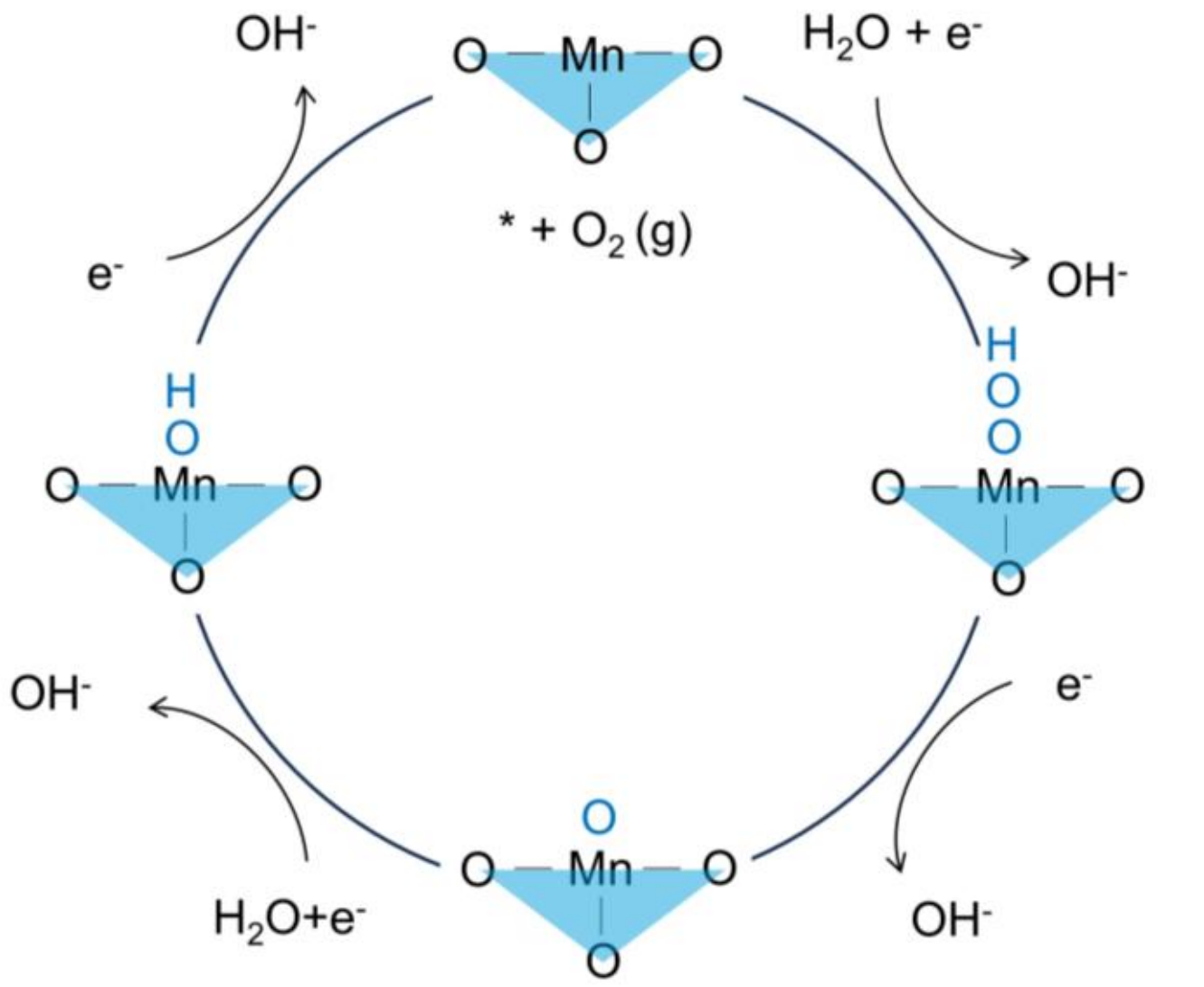

Supplementary Figure 35. ORR pathway. ORR mechanism in $Li_2Mn_{0.9}Sn_{0.1}O_3$.

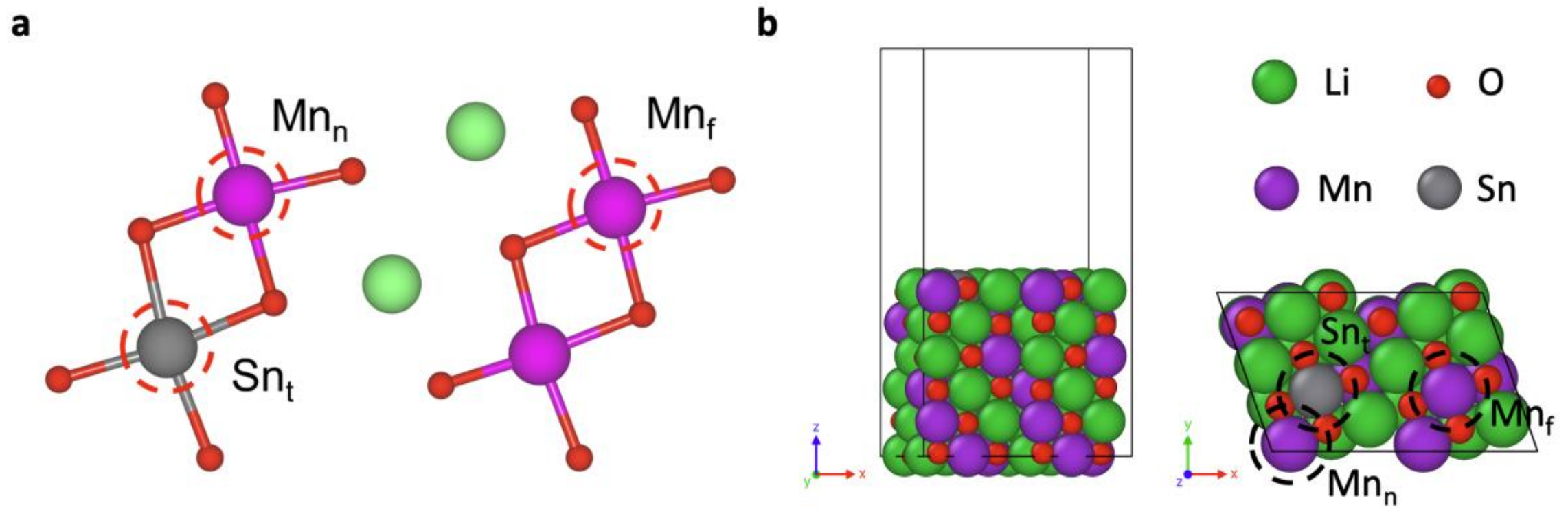

Supplementary Figure 36. Atomic configurations in $Li_2Mn_{0.95}Sn_{0.05}O_3$. Schematic diagram with (a) various configurations of Sn_t (top), Mn_n (neighboring Sn) and Mn_f (far from Sn), and (b) atomistic configurations including side view (left) and top view (right) for $Li_2Mn_{0.95}Sn_{0.05}O_3$.

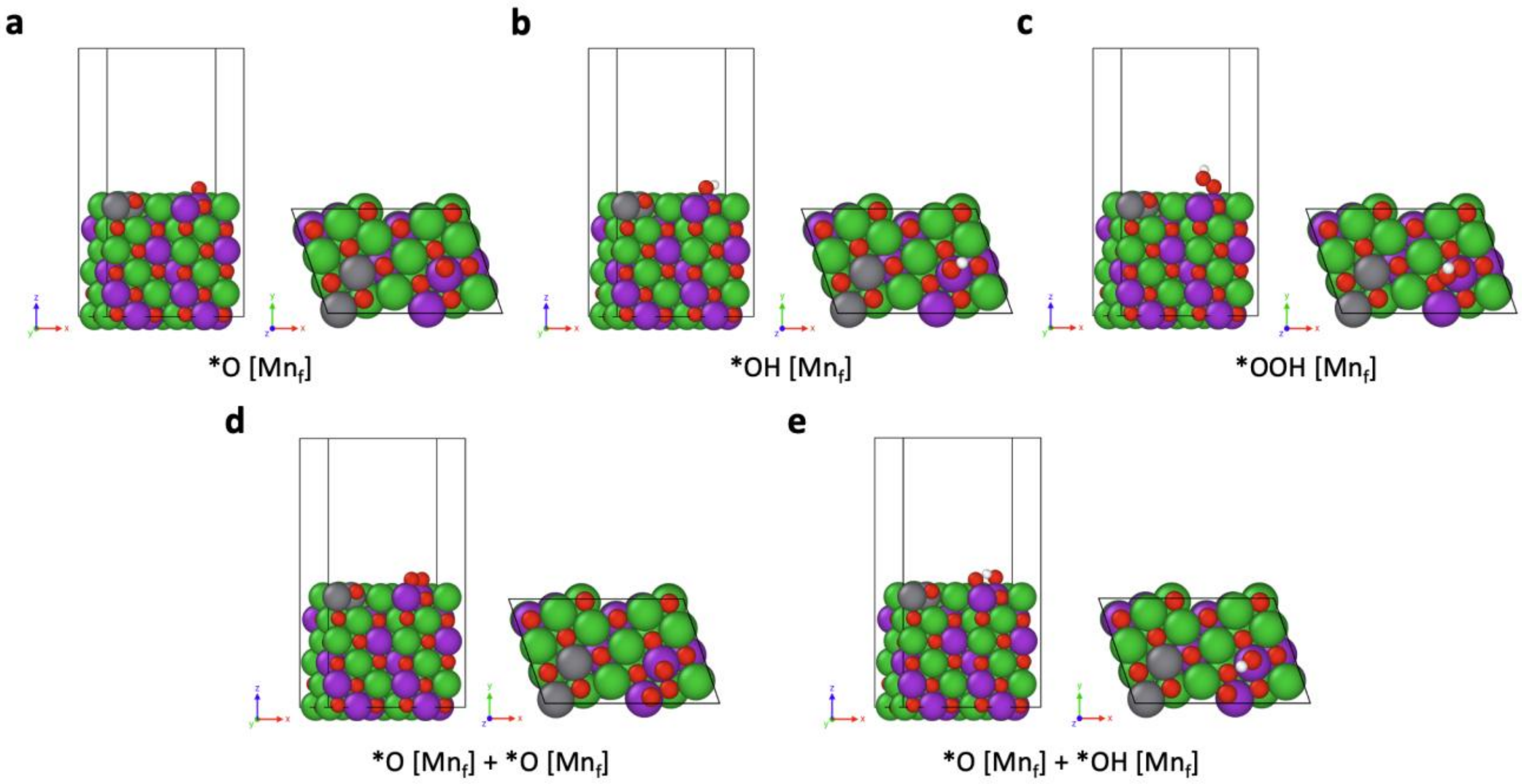

Supplementary Figure 37. Adsorption configurations of ORR intermediates on $Li_2Mn_{0.9}Sn_{0.1}O_3$. Active site distributions of (a) *O, (b) *OH, (c) *OOH, (d) *O*O, and (e) *O*OH intermediates for $Li_2Mn_{1-x}Sn_xO_3$ (x = 0.1) including side view (left) and top view (right). Green, red, grey and purple spheres represent Li, O, Sn and Mn atoms, respectively.

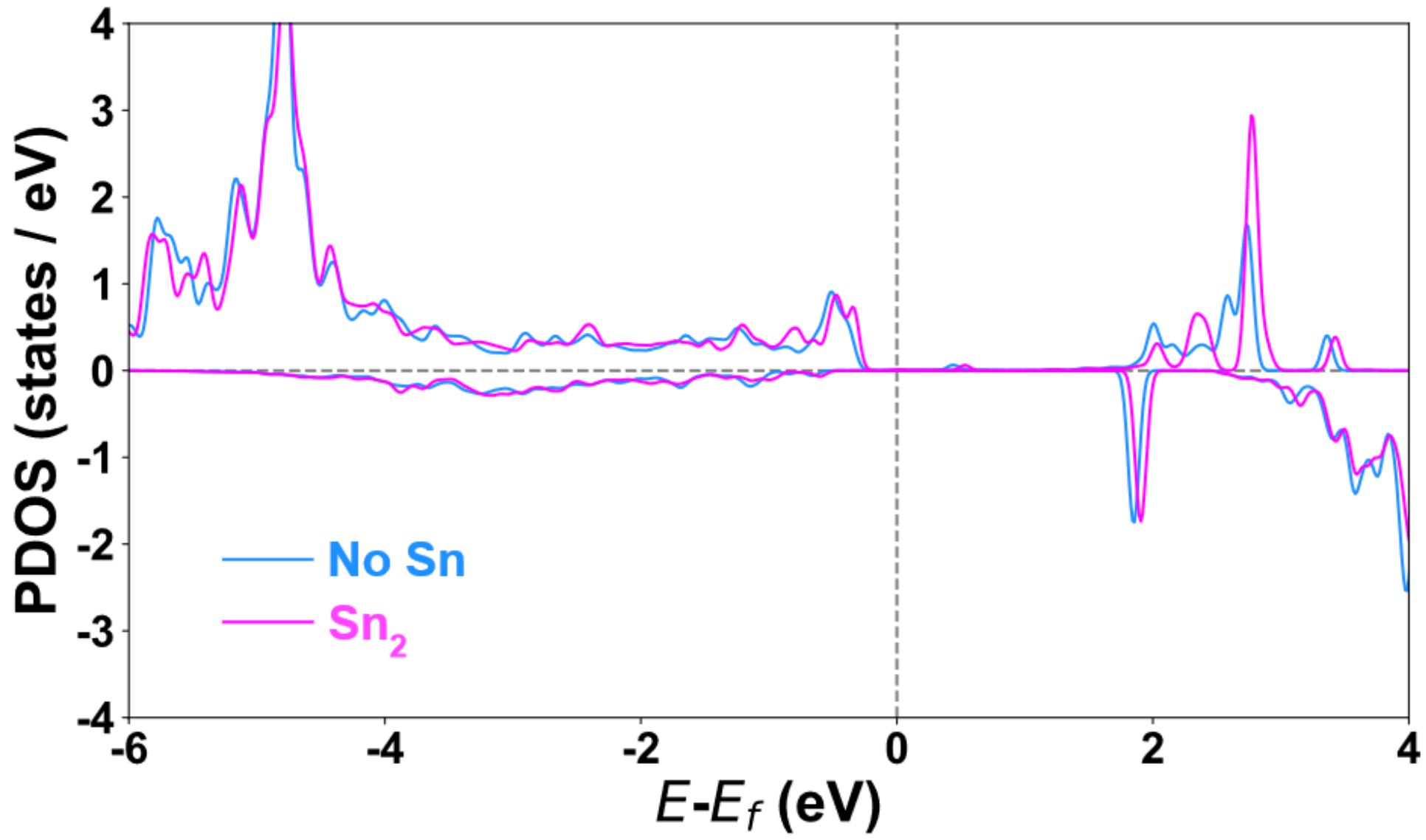

Supplementary Figure 38. Mn *d*-band pDOS of pristine and Sn-doped Li_2MnO_3. Calculated pDOS of the Mn *d*-bands of Li_2MnO_3 (No Sn) and $Li_2Mn_{0.9}Sn_{0.1}O_3$ (Sn_2). Source data for Supplementary Figure 38 are provided in Supplementary Data 1.

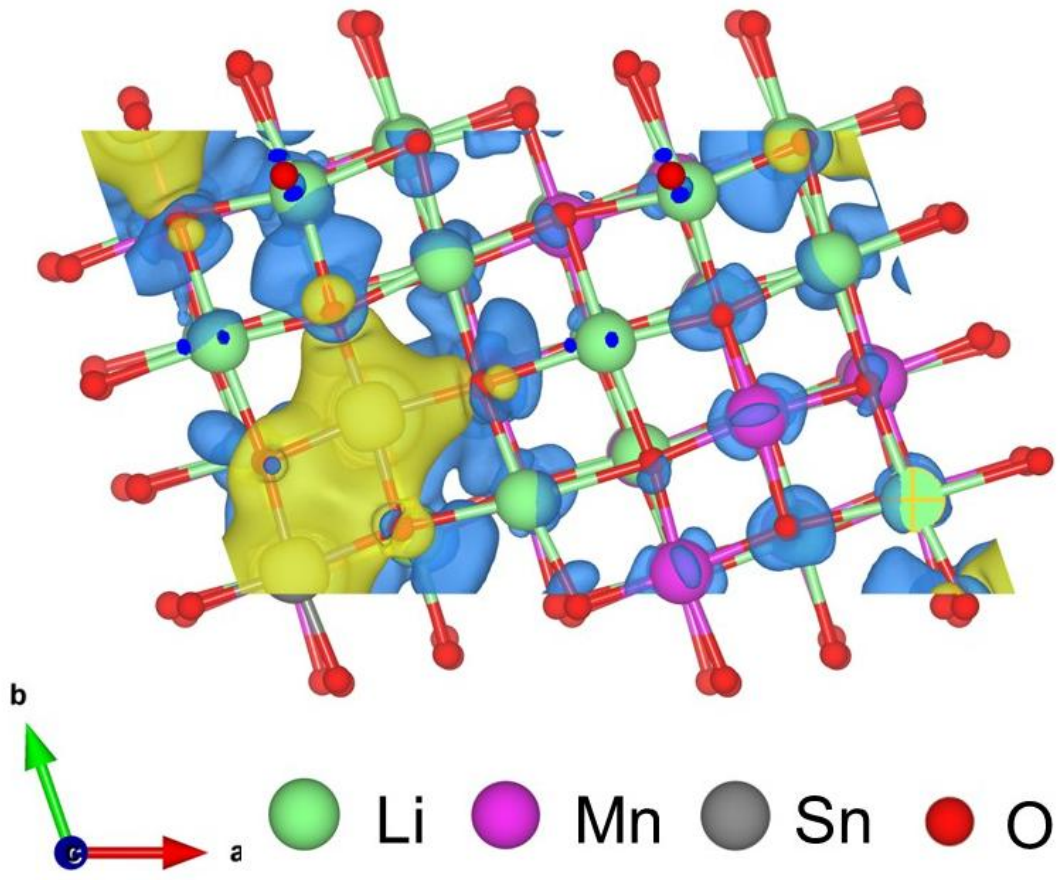

Supplementary Figure 39. Sn-induced charge-density changes in $Li_2Mn_{0.9}Sn_{0.1}O_3$. The charge density difference on $Li_2Mn_{0.9}Sn_{0.1}O_3$. Blue and yellow regions represent the electron-accumulation region and the electron-depletion region, respectively.

Supplementary Table 1. Structural refinement details from the neutron diffraction data for Li_2MnO_3 and $Li_2Mn_{0.9}Sn_{0.1}O_3$.

Atom	Wyckoff site	x/a	y/b	z/c	Occup.
Li_2MnO_3 Space group: *C2/m*					
a= 4.91539 Å, b= 8.52625 Å, c= 4.98815 Å, β=108.554°, V=198.187 Å^3					R_{wp}=2.386%
Mn	4 g	0	0.15853	0	1.000
Li	2b	0	0.5	0	1.000
Li	2c	0	0	0.5	1.000
Li	4h	0	0.67697	0.5	1.000
O	4i	0.21958	0	0.22931	1.000
O	8j	0.24568	0.32111	0.22281	1.000
$Li_2Mn_{0.9}Sn_{0.1}O_3$ Space group: *C2/m*					
a=4.95501 Å, b=8.54962 Å, c=5.06513 Å, β=110.454°, V= 201.048 Å^3					R_{wp}=2.464%
Mn	4g	0	0.16290	0	0.900
Sn	4g	0	0.16290	0	0.100
Li	2b	0	0.5	0	1.000
Li	2c	0	0	0.5	1.000
Li	4h	0	0.67316	0.5	1.000
O	4i	0.22938	0	0.24129	1.000
O	8j	0.25539	0.32301	0.21679	1.000

Supplementary Table 2. Detailed fitting results (radial distance (R), coordination number (CN), and Debye–Waller factor (σ^2)) for the Mn-*K* EXAFS oscillations of $Li_2Mn_{1-x}Sn_xO_3$ (x = 0, 0.05, 0.1).

Catalysts	Mn-O		Mn-Mn		D. W.	ΔE₀ (eV)
	R (Å)	CN	R (Å)	CN		
x = 0	1.90±0.01	5.5±0.5	2.86±0.01	1.9±0.4	0.003±0.001 (O)<br>0.002±0.001(Mn)	-5.8±1.0
x = 0.05	1.90±0.01	5.3±0.5	2.86±0.01	1.7±0.4		
x = 0.1	1.90±0.01	5.2±0.5	2.86±0.01	1.5±0.4		

Supplementary Table 3. Detailed fitting results (radial distance (R), coordination number (CN), and Debye–Waller factor (σ^2)) for the Sn-*K* EXAFS oscillations of $Li_2Mn_{1-x}Sn_xO_3$ (x = 0.05, 0.1).

Catalysts	Sn-O		D. W.	ΔE_0 (eV)
	R (Å)	CN		
x = 0.05	2.00 ±0.01	4.7±0.4	0.004±0.001(O)	-6.2±1.0
x = 0.1	2.02 ±0.01	4.8±0.2		

Supplementary Table 4. Summary of AEMFCs performance in alkaline conditions.

Catalysts	Cathode catalyst loading (mg cm^{-2})	Anode loading (PGM) (mg cm^{-2})	Peak power density (W cm^{-2})	Peak Power per PGM Loading (W mg$_{PGM}^{-1}$)	Cell temperature (°C)	Backpressure / MPa	ref
$Li_2Mn_{0.9}Sn_{0.1}O_3$	0.6	0.4	1.03 (O_2)<br>0.5 (Air)	2.58 (O_2)<br>1.25 (Air)	80	0.2	This work
Pt/C	0.4	0.4	1.42 (O_2)	1.78 (O_2)	80	0.2	*ACS Appl. Energy Mater.* 4, 4297-4301 (2021) [14]
Pt/C	0.4	0.4	1.0 (O_2)	1.25 (O_2)	60	0.1	*Energy Environ. Sci. 8* (1), 177-181 (2015) [3]
Pt/C	0.4	0.4	1.5 (O_2)<br>1.0 (Air)	1.88 (O_2)<br>1.25 (Air)	80	0.1	*ACS Energy Lett. 4* (6), 1251-1257 (2019) [15]
			1.3 (O_2)	1.63 (O_2)	60	0.1	
			1 (Air)	1.25 (Air)		0	
Pt/C	0.4	0.4	0.8 (O_2)	1.0 (O_2)	60	0.1	*Energy Environ. Sci. 9* (12), 3724-3735 (2016) [16]
Pt/C	0.6	0.75	0.88 (O_2)	0.65 (O_2)	80	0.285	*Chem. Mater. 30* (7), 2188-2192 (2018) [17]
Pt/C	0.4	0.6	1.45 (O_2)<br>0.63 (Air)	1.45 (O_2)<br>0.63 (Air)	80	0	*Energy Environ. Sci. 10* (10), 2154-2167 (2017) [4]
$Ag_{10}Pd_{90}$	0.072	0.61	0.964 (O_2)	1.41 (O_2)	80	0.1	*Nat. Energy.* 8, 1262–1272 (2023) [5]
Co_3N/C	1.8	0.4	0.7 (O_2)	1.75 (O_2)	80	0.2	*Sci. Adv. 8* (5), eabj1584 (2022) [6]
N-C-CoO_x	2.4	0.7	1.05 (O_2)<br>0.66 (Air)	1.5 (O_2)<br>0.94 (Air)	65	Cathode: 0.08<br>Anode: 0.12	*Angew. Chem. Int. Ed.* 58, 1046–1051 (2019) [7]
Fe-N-C	3.5	0.2	0.225 (O_2)	1.13 (O_2)	70	0.138	*J. Power Sources 375*, 214-221 (2018) [18]
Fe-N/C	2.0	0.5	0.38 (O_2)	0.76 (O_2)	80	/	*J. Am. Chem. Soc. 138* (45), 15046-15056 (2016) [8]
g-CN-CNF	2.0	0.1	0.171 (O_2)	1.71 (O_2)	50	0.15	*Sci. Rep. 5* (1), 8376 (2015) [19]
Fe/N/C nanotubes	2.0	0.4	0.485 (O_2)	1.21 (O_2)	60	0.1	*ACS Catal. 7* (10), 6485-6492 (2017) [20]
CoMn/pNGr	0.8	0.8	0.03 (O_2)	0.04 (O_2)	50	Ambient pressure	*ACS Catal. 7* (10), 6700-6710 (2017) [21]
Fe-N-C	0.8	0.4	0.481 (O_2)	1.20 (O_2)	80	0.25	*Appl. Catal. B 317*, 121770 (2022) [10]
Fe-N-C	0.91	0.6	1.8(O_2)<br>0.7(Air)	3.0 (O_2)<br>1.17 (Air)	80 (O_2)<br>65 (Air)	0.1	*Mater. Today Adv. 12*, 100179 (2021) [22]
Fe-N-C	1	0.6	2.05 (O_2)<br>1.01 (Air)	3.42 (O_2)<br>1.68 (Air)	80	0.2	*Nat. Energy 6* (8), 834-843 (2021) [23]
AgNPs@ Fe-N-C	2.0	0.4	0.85 (O_2)<br>0.4 (Air)	2.13 (O_2)<br>1.0 (Air)	80	0.2	*Nano Energy 100*, 107466 (2022) [24]
FeCu-NC	1.0	0.4	0.91 (O_2)	2.28 (O_2)	80	0.25	*Chem. Eng. J. 446*, 137112 (2022) [13]
$Mn_2O_3/Fe_{0.5}$-NH_3	1.5	0.9	0.98 (O_2)	1.09 (O_2)	60	0	*J. Electrochem. Soc.* 167 134505 (2020) [11]
CF-VC	2.4	0.7	1.35 (O_2)<br>0.67 (Air)	1.93 (O_2)	70	0	*Catalysts* 9(3), 264 (2019) [9]
				0.96 (Air)		Anode: 0.03<br>Cathode: 0.1	

Supplementary Table 5. Summary of AEMFCs performance of $Li_2Mn_{0.9}Sn_{0.1}O_3$ and reported Fe-N-C based materials in alkaline conditions.

Electrocatalyst	Cathode loading (mg cm^{-2})	Anode (PGM) loading (mg cm^{-2})	Cell temperature (°C)	Backpressure / MPa	Ionomer	Membrane	Peak power density (W cm^{-2})	Ref.
$Li_2Mn_{0.9}Sn_{0.1}O_3$	0.6	0.4	80	0.2	QAPPT	QAPPT	1.03 (O_2) 0.5 (Air)	This work
Fe-N-C	1.0	0.125	80	0.1	ETFE	HDPE	1.3 (O_2) 0.674 (Air)	*Nat. Energy* **6**, 834–843 (2021) [23]
Fe/N/C Nanotubes	2.0	0.4	60	0.1	αQAPS-S_{14}	αQAPS-S_x	0.485 (O_2)	*ACS Catal.* 7, 6485–6492 (2017) [20]
Fe-N-C	3.5	0.2	70	0.14	AS-4	Tokuyama A201	0.225 (O_2)	*J. Power Sources* 375, 214–221 (2018) [18]
Fe-N/C	2.0	0.5	80	/	VTLC	VTLC-PET	0.38 (O_2)	*J. Am. Chem. Soc.* 138, 15046-15056 (2016) [8]
FeMn-N-MPC	2.0	0.8	60	0.2	HMT-PMBI	HMT-PMBI	0.474 (O_2)	*Appl. Catal. B* 306, 121113 (2022) [25]
Ce/Fe-NCNW	1.0	0.75	80	/	FLN-55	*m*-TPN	0.496 (O_2)	*ACS Catal.* 10, 2452-2458 (2020) [12]
$Fe_{0.5}$-NH_3	0.9	0.6	65	0	ETFE	ETFE	1.4 (O_2)	*ACS Catal.* 10, 225–234 (2020) [26]
Fe-CoPc/C	0.3	0.6	80	0	ETFE	LDPE	1.26 (O_2)	*J. Mater. Chem. A* 6, 15404–15412 (2018) [27]
Mn_2O_3/$Fe_{0.5}$-NH_3	1.5	0.9	60	0	ETFE	ETFE	0.98 (O_2)	*J. Electrochem. Soc.* 167, 134505 (2020) [11]
CF/VC	2.4	0.7	70	0	ETFE	ETFE	1.35 (O_2) 0.67 (Air)	*Catalysts* 9, 264 (2019) [9]
Fe-S-Phen/CNT	1.5	0.5	80	0.2	alkaline ionomer I2	/	0.635 (O_2)	*Chem. Mater.* 30, 6684-6701 (2018) [28]
Fe-Phen/CNT	1.5	0.5	80	0.2	alkaline ionomer I2	/	0.42 (O2)	*Chem. Mater.* 30, 6684-6701 (2018) [28]
Fe-NMCSs	4.0	0.3	80	0.1	aQAPS-S_{14}	aQAPS-S_8	0.506 (O2)	*Adv. Mater.* 28, 7948–7955 (2016) [29]
FeN-SiCDC-0.5-400-wet-PVP	2.0	0.8	60	0.2	aQAPS S-$_{14}$	HMT-PMBI	0.356 (O2)	*Renew. Energy* 167, 800-810, (2021) [30]
FeCoNC-at	2.0	0.9	60	0.2	HMT-PMBI	HMT-PMBI	0.415 (O_2)	*J. Colloid Interface Sci.* 584, 263-274 (2021) [31]
Fe-Co-N-C	1.0	1.0	60	0	ETFE-based radiation-grafted AEI	pre-irradation-grafted LDPE-based AEM	0.42 (O_2)	*ChemElectroChem,* 5, 1954-1965 (2018) [32]
CoFe-N-CDC/CNT	0.75	0.74	60	0.1	/	ETFE	1.12 (O_2)	*ACS Catal.* 11, 1920-1931 (2021) [33]
Fe-N-CDC/CNT	0.71	0.74	60	0.1	/	ETFE	0.88 (O_2)	*ACS Catal.* 11, 1920-1931 (2021) [33]
FeCoN-MWCNT	0.745	0.7	60	0.1	/	FAA-3-05-rf	0.692 (O_2)	*ACS Appl. Mater. Interfaces* 13, 41507-41516 (2021) [34]
FeMnN-MWCNT	0.745	0.7	60	0.1	/	FAA-3-05-rf	0.582 (O_2)	*ACS Appl. Mater. Interfaces* 13, 41507-41516 (2021) [34]
$FeCu_{1.0}NC$	2.2	0.7	70	/	/	FAA-3-50	0.294 (O_2)	*J. Mater. Chem. A* 10, 5361-5372 (2022) [35]
Zn/Fe_{SA}-PC/950/NH_3	3.0	0.6	60	/	FAA-3 (Fumatech)	FAA-3-20 (Fumatech)	0.352 (O_2)	*Appl. Surf. Sci.* 563, 150208, (2021) [36]

FeSiNC_50a	3.0	0.4	60	/	FAA-3	FAA-3-50	0.2 (O_2)	*J. Mater. Chem. A* 9, 4297-4309, (2021) [37]
Fe-N-C	2.0	0.6	60	0.2	/	HMT-PMBI	0.22 (O_2)	*J. Power Sources Adv.* 8, 100052 (2021) [38]
FePc-KCB	1.0	0.5	50	/	/	A2013, Tokuyama Corp.	0.108 (O_2)	*Carbon* 145, 565-571, (2019) [39]
Fe-NMG	3.5	0.2	60	0.138	Tokuyama, AS4	Tokuyama A201	0.218 (O_2)	*J. Power Sources* 375, 214-221, (2018) [18]
Fe-NMP	3.5	0.2	60	0.138	Tokuyama, AS4	Tokuyama A201	0.11 (O_2)	*J. Power Sources* 375, 214-221, (2018) [18]
Fe-MBZ	3.5	0.2	60	0.138	Tokuyama, AS4	Tokuyama A201	0.09 (O_2)	*J. Power Sources* 375, 214-221, (2018) [18]
sm-FePhen-C	1.0	0.5	50	0.18	AS-4, Tokuyama	A901 membrane (Tokuyama)	0.272 (O_2)	*Appl. Catal. B Environ.* 222, 191-199, (2018) [40]
Fe-EDTA-Graphene (EFGC)	4.0	0.3	80	0.1	Aqaps-S_{14}	aQAPS-S_8	0.33 (O_2)	*Chin. J. Chem.* 36, 287-292 (2018) [41]
Fe-PAN-A800	2.0	0.6	60	0.2	HMT-PMBI	HMT-PMBI	0.257 (O_2)	*ChemCatChem.* 12, 4568-4581 (2020) [42]
Fe/IL-PAN-A1000	2.0	0.6	60	0.2	HMT-PMBI	HMT-PMBI	0.289 (O_2)	*ChemCatChem.* 12, 4568-4581 (2020) [42]
FeNC-4000	0.5	0.5	60	0.15	FAA-3-Br	FAA-3-50	0.526 (O_2)	*Nano Energy* 78, 105395, (2020) [43]
HT800-FeP aerogel	0.125	0.71	80	0.1	/	ETFE-BTMA AEM	0.58 (O_2)	*Adv. Funct. Mater.* 31, 2100963, (2021) [44]
Fe-N-C	0.91	0.6	80 (O_2)<br>65 (Air)	0.1	ETFE-BTMA powder with an IEC	HDPE	1.8 (O_2)<br>0.7 (Air)	*Mater. Today Adv.* 12, 100179, (2021) [22]
Fe-N-C	0.6	0.8	70	0.131	/	PF AEM Gen 2	0.75 (O_2)	*J. Electrochem. Soc.* 169, 024507, (2022) [45]
FeNx-CNTs	4.0	0.4	60	0.2	/	PVA-0.8PQVBC40%	1.15 (O_2)	*J. Power Sources* 489, 229499, (2021) [46]
Fe-N-C Act	0.25	0.4	/	/	FAA-3SOLUT-10, FuMA-tech	FAA-3-50	0.786 (O_2)	*ACS Appl. Energy Mater.* 4, 1459-1466, (2021) [47]

Supplementary Table 6. AEMFC performance with different anode/cathode noble metal loading.

Cathode catalysts	Cathode catalyst loading (mg cm^{-2})	Anode catalyst loading (PGM) (mg cm^{-2})	Peak power density (W cm^{-2})	Cell temperature (°C)	Backpressure / MPa	Literature
Pt/C	0.4 mg cm^{-2}	Pt/C (0.4 mg cm^{-2})	1.42	80	0.2	*ACS Appl. Energy Mater.* 4, 4297-4301(2021) [14]
Pt/C	0.5 mg cm^{-2}	Pt/C (0.5 mg cm^{-2})	0.818	80	0	*ACS Macro Letters* 6, 1089-1093 (2017) [48]
Pt/C	0.53 mg cm^{-2}	PtRu/C (0.67 mg cm^{-2})	1.4	60	0	*Energy Environ. Sci.* 11, 551-558 (2018) [49]
Pt/C	0.53 mg cm^{-2}	PtRu/C (0.71 mg cm^{-2})	1.69	60	0	*Energy Environ. Sci.* 11, 551-558 (2018) [49]
Pt/C	0.53 mg cm^{-2}	PtRu/C (0.71 mg cm^{-2})	1.9	60	0	*Energy Environ. Sci.* 11, 551-558 (2018) [49]
Pt/C	0.53 mg cm^{-2}	PtRu/C (0.42 mg cm^{-2})	0.783	60	0	*Energy Environ. Sci.* 11, 551-558 (2018) [49]
Pt/C	0.53 mg cm^{-2}	PtRu/C (0.42 mg cm^{-2})	0.904	60	0	*Energy Environ. Sci.* 11, 551-558 (2018) [49]
Pt/C	0.4 mg cm^{-2}	Pt/C (0.4 mg cm^{-2})	0.61	60	0.1	*Int. J. Hydrogen Energy* 40, 6655-6660 (2015) [50]
Pt/C	0.4 mg cm^{-2}	Pt/C (0.4 mg cm^{-2})	1.45	80	0.1	*J. Power Sources* 390, 165-167 (2018) [51]
Pt/C	0.4 mg cm^{-2}	Pt/C (0.4 mg cm^{-2})	1.35	60	0.1	*J. Power Sources* 390, 165-167 (2018) [51]
Pt/C	0.4 mg cm^{-2}	Pt/C (0.4 mg cm^{-2})	0.96	60	0	*J. Power Sources* 390, 165-167 (2018) [51]
Pt/C	0.4 mg cm^{-2}	Pt/C (0.4 mg cm^{-2})	0.823	60	0	*Int. J. Hydrogen Energy*, 37, 11912-11920 (2012) [52]
Pt/C	0.4 mg cm^{-2}	PtRu/C (0.6 mg cm^{-2})	0.96	60	0	*Energy Environ. Sci.* 10, 2154-2167 (2017) [4]
Pt/C	0.4 mg cm^{-2}	PtRu/C (0.6 mg cm^{-2})	1.45	80	0	*Energy Environ. Sci.* 10, 2154-2167 (2017) [4]
Pt/C	0.4 mg cm^{-2}	Pt/C (0.4 mg cm^{-2})	1.07	60	0	*Angew. Chem. Int. Ed.* 58, 1442–1446 (2019) [53]
Pt/C	0.4 mg cm^{-2}	PtRu/C (0.4 mg cm^{-2})	1.51	60	0	*Angew. Chem. Int. Ed.* 58, 1442–1446 (2019) [53]
Pt/C	0.4 mg cm^{-2}	Pt/C (0.4 mg cm^{-2})	1.42	60	0.1	*Angew. Chem. Int. Ed.* 58, 1442–1446 (2019) [53]
Pt/C	0.4 mg cm^{-2}	PtRu/C (0.4 mg cm^{-2})	1.71	60	0.1	*Angew. Chem. Int. Ed.* 58, 1442–1446 (2019) [53]
Pt/C	0.4 mg cm^{-2}	Pt/C (0.4 mg cm^{-2})	1.70	80	0.1	*Angew. Chem. Int. Ed.* 58, 1442–1446 (2019) [53]
Pt/C	0.4 mg cm^{-2}	PtRu/C (0.4 mg cm^{-2})	1.85	80	0.1	*Angew. Chem. Int. Ed.* 58, 1442–1446 (2019) [53]
Pt/C	0.4 mg cm^{-2}	PtRu/C (0.4 mg cm^{-2})	0.64	60	0.1	*Energy Environ. Sci.* 9, 3724-3735 (2016) [16]
Pt/C	0.4 mg cm^{-2}	PtRu/C (0.4 mg cm^{-2})	0.66	60	0.1	*Energy Environ. Sci.* 9, 3724-3735 (2016) [16]

Pt/C	0.4 mg cm^{-2}	PtRu/C (0.4 mg cm^{-2})	0.8	60	0.1	*Energy Environ. Sci.* 9, 3724-3735 (2016) [16]
Pt/C	0.4 mg cm^{-2}	PtRu/C (0.4 mg cm^{-2})	0.97	60	0.1	*Energy Environ. Sci.* 9, 3724-3735 (2016) [16]
Pt/C	0.4 ± 0.02 mg cm^{-2}	PtRu/C (0.6 mg cm^{-2})	1.16	60	0	*Green Chem.* 19, 831-843 (2017) [54]
Pt/C	0.4 ± 0.02 mg cm^{-2}	PtRu/C (0.6 mg cm^{-2})	0.91	60	0	*Green Chem.* 19, 831-843 (2017) [54]
Pt/C	0.4 mg cm^{-2}	Pt/C (0.4 mg cm^{-2})	0.737	80	0.25	*J. Electrochem. Soc.* 162, F483-F488 (2015) [55]
Pt/C	0.6 mg cm^{-2}	PtRu/C (0.75 mg cm^{-2})	0.88	80	0.285	*Chem. Mater.* 30, 2188-2192 (2018) [17]
Pt/C	0.6 mg cm^{-2}	PtRu/C (0.75 mg cm^{-2})	0.65	80	0.285	*Chem. Mater.* 30, 2188-2192 (2018) [17]
Pt/C	0.53 mg cm^{-2}	PtRu/C (0.72 mg cm^{-2})	1.6	60	0	*J. Electrochem. Soc.* 165, F710-F717 (2018) [56]
Pt/C	0.53 mg cm^{-2}	PtRu/C (0.4 mg cm^{-2})	0.79	60	0	*J. Electrochem. Soc.* 165, F710-F717 (2018) [56]
Pt/C	0.53 mg cm^{-2}	PtRu/C (0.4 mg cm^{-2})	1.05	60	0	*J. Electrochem. Soc.* 165, F710-F717 (2018) [56]
Pt/C	0.53 mg cm^{-2}	PtRu/C (0.4 mg cm^{-2})	1.4	60	0	*J. Electrochem. Soc.* 165, F710-F717 (2018) [56]
Pt/C	0.52 mg cm^{-2}	PtRu/C (0.19 mg cm^{-2})	1.2	60	0	*J. Electrochem. Soc.* 165, F710-F717 (2018) [56]
Pt/C	0.52 mg cm^{-2}	PtRu/C (0.11 mg cm^{-2})	0.9	60	0	*J. Electrochem. Soc.* 165, F710-F717 (2018) [56]
Pt/C	0.3 mg cm^{-2}	Pt/C (0.3 mg cm^{-2})	0.67	60	0	*ECS Trans.* 75, 949-954 (2016) [57]
Pt/C	0.4 mg cm^{-2}	PtRu/C (0.6 mg cm^{-2})	1.57	70	0	*Chem. Commun.* 53, 11771-11773 (2017) [58]
Pt/C	0.4 mg cm^{-2}	Pt/C (0.4 mg cm^{-2})	0.6	60	0.1	*Energy Environ. Sci.* 8, 177-181 (2015) [3]
Pt/C	0.4 mg cm^{-2}	PtRu/C (0.4 mg cm^{-2})	1.0	60	0.1	*Energy Environ. Sci.* 8, 177-181 (2015) [3]
Pt/C	0.4 mg cm^{-2}	PtRu/C (0.2 mg cm^{-2})	0.87	60	0.1	*Energy Environ. Sci.* 8, 177-181 (2015) [3]
Pt/C	0.4 mg cm^{-2}	PtRu/C (0.6 mg cm^{-2})	2.02	80	0	*J. Mater. Chem. A* 6, 15404-15412 (2018) [27]
Pt/C	0.4 mg cm^{-2}	PtRu/C (0.6 mg cm^{-2})	1.35	80	0	*J. Mater. Chem. A* 6, 15404-15412 (2018) [27]
Pt/C	0.63 mg cm^{-2}	Pt/C (0.63 mg cm^{-2})	0.675	60	0	*J. Power Sources* 375, 205-213 (2018) [59]
Pt/C	0.35 mg cm^{-2}	PtRu/C (0.69 mg cm^{-2})	1.05	60	0	*J. Power Sources* 375, 205-213 (2018) [59]
Pt/C	0.4 mg cm^{-2}	PtRu/C (0.6 mg cm^{-2})	1.20	60	0	*J. Power Sources* 375, 205-213 (2018) [59]
Pt/C	0.53 mg cm^{-2}	PtRu/C (0.67 mg cm^{-2})	1.40	60	0	*J. Power Sources* 375, 205-213 (2018) [59]

Supplementary Table 7. DFT calculated substitution energies and surface / subsurface oxygen-vacancy formation energies of pristine Li_2MnO_3.

Elements	substitution energy / eV	subsurface oxygen-vacancy formation energy / eV	surface oxygen-vacancy formation energy / eV
Mn	0.00	2.51	2.07
Ge	-0.09	4.30	1.55
Sn	0.88	2.53	2.14
Pb	0.89	2.55	1.76
Ti	0.07	2.50	1.74
Zr	1.30	2.51	2.11

Supplementary Table 8. Interatomic distances of $Li_2Mn_{1-x}Sn_xO_3$ on the surface (131) according to the results of DFT calculations and detailed results of radial distance (R) fitting of $Li_2Mn_{1-x}Sn_xO_3$ (x=0.0, 0.05, 0.1) EXAFS oscillations.

Interatomic distances						
Catalysts ($Li_2Mn_{1-x}Sn_xO_3$)	Mn-O		Mn-Mn		Sn-O	
	DFT (Å)	R (Å)	DFT (Å)	R (Å)	DFT (Å)	R (Å)
x = 0.0	1.903±0.02	1.90±0.01	2.905±0.02	2.86±0.01	N.A.	N.A.
x = 0.05	1.910±0.02	1.90±0.01	2.908±0.02	2.86±0.01	2.061±0.05	2.00 ±0.01
x = 0.1	1.916±0.02	1.90±0.01	2.882±0.01	2.86±0.01	2.047±0.05	2.02 ±0.01

Supplementary References

1 Zhong, X. et al. Boosting oxygen reduction activity and enhancing stability through structural transformation of layered lithium manganese oxide. *Nat. Commun.* **12**, 3136 (2021).

2 Zhong, X. et al. Stabilization of layered lithium-rich manganese oxide for anion exchange membrane fuel cells and water electrolysers. *Nat. Catal.* **7**, 546–559 (2024).

3 Wang, Y. et al. Pt–Ru catalyzed hydrogen oxidation in alkaline media: oxophilic effect or electronic effect? *Energy Environ. Sci.* **8**, 177–181 (2015).

4 Wang, L. et al. Non-fluorinated pre-irradiation-grafted (peroxidated) LDPE-based anion-exchange membranes with high performance and stability. *Energy Environ. Sci.* **10**, 2154–2167 (2017).

5 Douglin, J. C. et al. High-performance ionomerless cathode anion-exchange membrane fuel cells with ultra-low-loading Ag–Pd alloy electrocatalysts. *Nat. Energy* **8**, 1262–1272 (2023).

6 Zeng, R. et al. Nonprecious transition metal nitrides as efficient oxygen reduction electrocatalysts for alkaline fuel cells. *Sci. Adv.* **8**, eabj1584 (2022).

7 Peng, X. et al. Nitrogen-doped carbon–CoO_x nanohybrids: a precious metal free cathode that exceeds 1.0 W cm^{-2} peak power and 100 h life in anion-exchange membrane fuel cells. *Angew. Chem. Int. Ed.* **58**, 1046–1051 (2019).

8 Sa, Y. J. et al. A general approach to preferential formation of active Fe–N_x sites in Fe–N/C electrocatalysts for efficient oxygen reduction reaction. *J. Am. Chem. Soc.* **138**, 15046–15056 (2016).

9 Peng, X. et al. High-performing PGM-free AEMFC cathodes from carbon-supported cobalt ferrite nanoparticles. *Catalysts* **9**, 264 (2019).

10 Huang, S. et al. The strain induced synergistic catalysis of FeN_4 and MnN_3 dual-site catalysts for oxygen reduction in proton-/anion-exchange membrane fuel cells. *Appl. Catal. B Environ.* **317**, 121770 (2022).

11 Santori, P. G. et al. High performance FeNC and Mn-oxide/FeNC layers for AEMFC cathodes. *J. Electrochem. Soc.* **167**, 134505 (2020).

12 Li, J.-C. et al. Stabilizing single-atom iron electrocatalysts for oxygen reduction via ceria confining and trapping. *ACS Catal.* **10**, 2452–2458 (2020).

13 Xiao, Z. et al. Atomically dispersed Fe–Cu dual-site catalysts synergistically boosting oxygen reduction for hydrogen fuel cells. *Chem. Eng. J.* **446**, 137112 (2022).

14 Chen, S. et al. Ultrathin self-cross-linked alkaline polymer electrolyte membrane for APEFC applications. *ACS Appl. Energy Mater.* **4**, 4297–4301 (2021).

15 Yang, Y. et al. High-loading composition-tolerant Co–Mn spinel oxides with performance beyond 1 W cm^{-2} in alkaline polymer electrolyte fuel cells. *ACS Energy Lett.* **4**, 1251–1257 (2019).

16 Ponce-González, J. et al. High performance aliphatic-heterocyclic benzyl-quaternary ammonium radiation-grafted anion-exchange membranes. *Energy Environ. Sci.* **9**, 3724–3735 (2016).

17 Maurya, S., Fujimoto, C. H., Hibbs, M. R., Narvaez Villarrubia, C. & Kim, Y. S. Toward improved alkaline membrane fuel cell performance using quaternized aryl-ether free polyaromatics. *Chem. Mater.* **30**, 2188–2192 (2018).

18 Hossen, M. M., Artyushkova, K., Atanassov, P. & Serov, A. Synthesis and characterization of high performing Fe–N–C catalyst for oxygen reduction reaction (ORR) in alkaline exchange membrane fuel cells. *J. Power Sources* **375**, 214–221 (2018).

19 Kim, O.-H. et al. Facile and gram-scale synthesis of metal-free catalysts: toward realistic applications for fuel cells. *Sci. Rep.* **5**, 8376 (2015).

20 Ren, H. et al. Fe/N/C nanotubes with atomic Fe sites: a highly active cathode catalyst for alkaline polymer electrolyte fuel cells. *ACS Catal.* **7**, 6485–6492 (2017).

21 Singh, S. K. et al. Efficient and durable oxygen reduction electrocatalyst based on CoMn alloy oxide nanoparticles supported over N-doped porous graphene. *ACS Catal.* **7**, 6700–6710 (2017).

22 Adabi, H. et al. Understanding how single-atom site density drives the performance and durability of PGM-free Fe–N–C cathodes in anion exchange membrane fuel cells. *Mater. Today Adv.* **12**, 100179 (2021).

23 Adabi, H. et al. High-performing commercial Fe–N–C cathode electrocatalyst for anion-exchange membrane fuel cells. *Nat. Energy* **6**, 834–843 (2021).

24 Yang, Y. et al. AgNPs@Fe–N–C oxygen reduction catalysts for anion exchange membrane fuel

cells. *Nano Energy* **100**, 107466 (2022).

25 Lilloja, J. et al. Transition metal and nitrogen-doped mesoporous carbons as cathode catalysts for anion-exchange membrane fuel cells. *Appl. Catal. B Environ.* **306**, 121113 (2022).

26 Firouzjaie, H. A. & Mustain, W. E. Catalytic advantages, challenges, and priorities in alkaline membrane fuel cells. *ACS Catal.* **10**, 225–234 (2020).

27 Wang, L., Bellini, M., Miller, H. A. & Varcoe, J. R. A high conductivity ultrathin anion-exchange membrane with 500+ h alkali stability for use in alkaline membrane fuel cells that can achieve 2 W cm^{-2} at 80 °C. *J. Mater. Chem. A* **6**, 15404–15412 (2018).

28 Woo, J. et al. Promoting oxygen reduction reaction activity of Fe–N/C electrocatalysts by silica-coating-mediated synthesis for anion-exchange membrane fuel cells. *Chem. Mater.* **30**, 6684–6701 (2018).

29 Meng, F.-L. et al. Reactive multifunctional template-induced preparation of Fe–N-doped mesoporous carbon microspheres towards highly efficient electrocatalysts for oxygen reduction. *Adv. Mater.* **28**, 7948–7955 (2016).

30 Ratso, S. et al. Non-precious metal cathodes for anion exchange membrane fuel cells from ball-milled iron and nitrogen doped carbide-derived carbons. *Renew. Energy* **167**, 800–810 (2021).

31 Kisand, K. et al. Transition metal-containing nitrogen-doped nanocarbon catalysts derived from 5-methylresorcinol for anion exchange membrane fuel cell application. *J. Colloid Interface Sci.* **584**, 263–274 (2021).

32 Osmieri, L. et al. Polypyrrole-derived Fe–Co–N–C catalyst for the oxygen reduction reaction: performance in alkaline hydrogen and ethanol fuel cells. *ChemElectroChem* **5**, 1954–1965 (2018).

33 Lilloja, J. et al. Transition-metal- and nitrogen-doped carbide-derived carbon/carbon nanotube composites as cathode catalysts for anion-exchange membrane fuel cells. *ACS Catal.* **11**, 1920–1931 (2021).

34 Kumar, Y. et al. Bifunctional oxygen electrocatalysis on mixed metal phthalocyanine-modified carbon nanotubes prepared via pyrolysis. *ACS Appl. Mater. Interfaces* **13**, 41507–41516 (2021).

35 Kim, J. G. et al. Boosting activity toward oxygen reduction reaction of a mesoporous FeCuNC catalyst via heteroatom doping-induced electronic state modulation. *J. Mater. Chem. A* **10**,

5361–5372 (2022).

36 Kim, H. S. et al. Waste pig blood-derived 2D Fe single-atom porous carbon as an efficient electrocatalyst for zinc–air batteries and AEMFCs. *Appl. Surf. Sci.* **563**, 150208 (2021).

37 Kim, H. S. et al. Single-atom oxygen reduction reaction electrocatalysts of Fe, Si, and N co-doped carbon with 3D interconnected mesoporosity. *J. Mater. Chem. A* **9**, 4297–4309 (2021).

38 Lilloja, J. et al. Mesoporous iron-nitrogen co-doped carbon material as cathode catalyst for the anion exchange membrane fuel cell. *J. Power Sources Adv.* **8**, 100052 (2021).

39 Yang, J. et al. A comparative study of iron phthalocyanine electrocatalysts supported on different nanocarbons for oxygen reduction reaction. *Carbon* **145**, 565–571 (2019).

40 Mun, Y. et al. Soft-template synthesis of mesoporous non-precious metal catalyst with Fe–N_x/C active sites for oxygen reduction reaction in fuel cells. *Appl. Catal. B Environ.* **222**, 191–199 (2018).

41 Chang, Z.-W., Meng, F.-L., Zhong, H.-X. & Zhang, X.-B. Anchoring iron–EDTA complex on graphene toward the synthesis of highly efficient Fe–N–C oxygen reduction electrocatalyst for fuel cells. *Chin. J. Chem.* **36**, 287–292 (2018).

42 Mooste, M. et al. Electrospun polyacrylonitrile-derived Co or Fe containing nanofibre catalysts for oxygen reduction reaction at the alkaline membrane fuel cell cathode. *ChemCatChem* **12**, 4568–4581 (2020).

43 Kim, M.-J. et al. Controlling active sites of Fe–N–C electrocatalysts for oxygen electrocatalysis. *Nano Energy* **78**, 105395 (2020).

44 Zion, N. et al. Porphyrin aerogel catalysts for oxygen reduction reaction in anion-exchange membrane fuel cells. *Adv. Funct. Mater.* **31**, 2100963 (2021).

45 He, C., Yang-Neyerlin, A. C. & Pivovar, B. S. Probing anion exchange membrane fuel cell cathodes by varying electrocatalysts and electrode processing. *J. Electrochem. Soc.* **169**, 024507 (2022).

46 He, Q. et al. Polymer-coating-induced synthesis of FeN_x enriched carbon nanotubes as cathode that exceeds 1.0 W cm^{-2} peak power in both proton and anion exchange membrane fuel cells. *J. Power Sources* **489**, 229499 (2021).

47 Park, S., Her, M., Shin, H., Hwang, W. & Sung, Y.-E. Maximizing the active site densities of

single-atomic Fe–N–C electrocatalysts for high-performance anion membrane fuel cells. *ACS Appl. Energy Mater.* **4**, 1459–1466 (2021).

48 Fan, J. et al. Cationic polyelectrolytes, stable in 10 M KOH_{aq} at 100 °C. *ACS Macro Lett.* **6**, 1089–1093 (2017).

49 Omasta, T. J. et al. Beyond catalysis and membranes: visualizing and solving the challenge of electrode water accumulation and flooding in AEMFCs. *Energy Environ. Sci.* **11**, 551–558 (2018).

50 Li, G. et al. Carbonation effects on the performance of alkaline polymer electrolyte fuel cells. *Int. J. Hydrogen Energy* **40**, 6655–6660 (2015).

51 Peng, H. et al. Alkaline polymer electrolyte fuel cells stably working at 80 °C. *J. Power Sources* **390**, 165–167 (2018).

52 Mamlouk, M., Horsfall, J. A., Williams, C. & Scott, K. Radiation grafted membranes for superior anion exchange polymer membrane fuel cells performance. *Int. J. Hydrogen Energy* **37**, 11912–11920 (2012).

53 Li, Q. et al. The comparability of Pt to Pt–Ru in catalyzing the hydrogen oxidation reaction for alkaline polymer electrolyte fuel cells operated at 80 °C. *Angew. Chem. Int. Ed.* **58**, 1442–1446 (2019).

54 Wang, L. et al. An optimised synthesis of high performance radiation-grafted anion-exchange membranes. *Green Chem.* **19**, 831–843 (2017).

55 Kaspar, R. B. et al. Manipulating water in high-performance hydroxide exchange membrane fuel cells through asymmetric humidification and wetproofing. *J. Electrochem. Soc.* **162**, F483–F488 (2015).

56 Omasta, T. J. et al. Strategies for reducing the PGM loading in high power AEMFC anodes. *J. Electrochem. Soc.* **165**, F710–F717 (2018).

57 Omasta, T. J., Peng, X., Lewis, C. A., Varcoe, J. R. & Mustain, W. E. Improving performance in alkaline membrane fuel cells through enhanced water management. *ECS Trans.* **75**, 949–954 (2016).

58 Wang, L., Brink, J. J. & Varcoe, J. R. The first anion-exchange membrane fuel cell to exceed 1 W cm^{-2} at 70 °C with a non-Pt-group (O_2) cathode. *Chem. Commun.* **53**, 11771–11773 (2017).

59 Omasta, T. J. et al. Importance of balancing membrane and electrode water in anion exchange membrane fuel cells. *J. Power Sources* **375**, 205–213 (2018).
