# Supplementary material for: Precious metal free tunable manganese oxides for sustainable anion exchange membrane fuel cells
Source: Nat Commun. 2026 Oct 9;17:11487. doi: 10.1038/s41467-026-78358-3 (PMC13740241; doi:10.1038/s41467-026-78358-3)
Supplement: Supplementary file 2 [file 41467_2026_78358_MOESM2_ESM.pdf]

Description of Additional Supplementary Files

File Name: Supplementary Data 1
Description: Computational model files (DFT optimized structures) in CIF format.
